# NECA alleviates inflammatory responses in diabetic retinopathy through dendritic cell toll-like receptor signaling pathway

**DOI:** 10.3389/fimmu.2024.1415004

**Published:** 2024-06-04

**Authors:** Lanjiao Li, Jichun Chen, Zhenyan Wang, Yan Xu, Hao Yao, Wulong Lei, Xiyuan Zhou, Minming Zheng

**Affiliations:** The Second Affiliated Hospital of Chongqing Medical University, Chongqing Key Lab of Ophthalmology, Chongqing, China

**Keywords:** NECA, diabetic retinopathy, dendritic cells, toll-like receptor, Treg cell, Th17 cell

## Abstract

**Introduction:**

This study examined the impact of 5’-(N- ethylcarboxamido)adenosine (NECA) in the peripheral blood of healthy individuals, those with diabetes mellitus, diabetic retinopathy (DR), and C57BL/6 mice, both *in vivo* and *in vitro*.

**Methods:**

Enzyme-linked immunosorbent assay (ELISA) and flow cytometry (FCM) were used to evaluate the effects of NECA on dendritic cells (DCs) and mouse bone marrow-derived dendritic cells (BMDCs) and the effects of NECA-treated DCs on Treg and Th17 cells. The effect of NECA on the Toll-like receptor (TLR) pathway in DCs was evaluated using polymerase chain reaction (PCR) and western blotting (WB).

**Results:**

FCM and ELISA showed that NECA inhibited the expression of surface markers of DCs and BMDCs, increased anti-inflammatory cytokines and decreased proinflammatory cytokines. PCR and WB showed that NCEA decreased mRNA transcription and protein expression in the TLR-4-MyD88-NF-kβ pathway in DCs and BMDCs. The DR severity in streptozocin (STZ) induced diabetic mice was alleviated. NECA-treated DCs and BMDCs were co-cultivated with CD4+T cells, resulting in modulation of Treg and Th17 differentiation, along with cytokine secretion alterations.

**Conclusion:**

NECA could impair DCs’ ability to present antigens and mitigate the inflammatory response, thereby alleviating the severity of DR.

## 1 Introduction

As societal living standards enhance, there is a corresponding rise in diabetes incidence (1–3). DR, a significant complication of diabetes mellitus, is becoming increasingly prevalent. Data from the International Diabetes Federation indicates that in 2021, there were 537 million adults aged 20 to 79 years with diabetes. Projections suggest this number will escalate to 783 million by 2045 (4). It is anticipated that DR cases will surge to 160.5 million by the year 2045 (5). Although the underlying mechanisms of DR are complex, numerous studies have indicated a strong correlation between the pathogenesis of DR and inflammatory processes (6, 7). Genome-wide association studies found that inflammatory genes are closely related to DR (8).

DCs stand as vital antigen-presenting cells within the body. These cells excel in capturing, processing, and presenting antigens, effectively activating and proliferating naive T cells. They hold a central role in initiating, modulating, and sustaining specific immune responses. Toll-like receptors (TLRs) are a class of transmembrane proteins mainly expressed in antigen-presenting cells like DCs and macrophages (9). TLRs expressed on DC can bind to their corresponding ligands and cause DC activation (10, 11), which are vital in the host immune system’s defense against pathogens.

Over the past few years, more and more studies have highlighted the significance of gut bacteria and the compounds they produce in maintaining immune balance and controlling inflammation. In our preceding research, we noted a distinctive fecal adenosine composition in DR patients compared to healthy individuals (12). Adenosine is a metabolic intermediate involved in the adenosine triphosphate (ATP) catabolic pathway. Moreover, adenosine is an endogenous nucleoside widely distributed in mammals that regulates various important physiological functions by binding to its receptors (13–15). Under physiological and non-stress conditions, extracellular adenosine concentration is maintained at a low level due to rapid metabolism and uptake, and its level rises substantially in response to increasing metabolic demand, hypoxia, tissue damage, and inflammation to regulate the immune response (16). A variety of immune cells are known to express adenosine receptors, showing diverse responses to adenosine and its agonists (17, 18). Research by Deaglio et al. revealed that T regulatory cells (Tregs) transform extracellular ATP into adenosine through the CD39/CD73 enzymatic pathway. This process activates adenosine-2A (A2A) receptors on T effector cells, resulting in the inhibition of their function and the suppression of pro-inflammatory mediator release (16, 19). With the activation of TLRs on macrophages, nuclear factor kappa B (NF-κB) and hypoxia-inducible factor-1-mediated expression of A2A and A2B receptors increased, making these cells most sensitive to extracellular adenosine. Upon activation, these receptors induce a shift in macrophages, transitioning them from the production of pro-inflammatory agents such as TNF, interleukin (IL)-12, and CXC motif chemokine ligands 1 and 2, towards anti-inflammatory factors, particularly IL-10, and promoting a change to the M2 phenotype (20–23).

While the role of adenosine and TLRs in DC has been extensively explored, their study in the context of DR patients remains limited. Elucidating the interplay between DR and inflammatory mediators could pave the way for innovative approaches in the diagnosis, prevention, and management of DR. Although adenosine acts as a local regulator with cellular protective functions, extracellular adenosine is usually rapidly taken up by neighboring cells and rapidly disappears. Hence, our study focused on examining the impact of NECA, a potent and non-selective adenosine receptor agonist, on the DCs in individuals with DR, particularly investigating its interactions with the TLRs pathway. In this study, we aimed to develop new therapeutic approaches for DR.

## 2 Materials and methods

### 2.1 Patients

From August 2021 to August 2022, a study was undertaken at the Second Affiliated Hospital of Chongqing Medical University, Chongqing, China, involving 60 subjects. This group included 20 patients with DR (12 males, 8 females), 20 patients with DM (11 females, 9 males), and 20 individuals as healthy controls (10 females, 10 males), with a close age match across groups (average ages: 60.34 ± 7.09, 59.13 ± 7.68, 60.43 ± 5.98 years, respectively). Patients with DM were diagnosed following the 2018 ADA guidelines, with DR exclusion verified through fundus microscopy and optical coherence tomography. For DR inclusion, criteria entailed diagnosis following the 2018 ADA guidelines and via prior slit-lamp biomicroscopy, direct fundus, and fluorescein angiography, excluding other ocular or systemic diseases with ocular impacts. Participants were excluded for history of autoimmune diseases (like arthritis, lupus, dermatomyositis, ankylosing spondylitis, uveitis), type 1 diabetes or unidentified diabetes etiology, high blood pressure, malignant tumors, or prior organ transplants. Controls were selected for their lack of systemic diseases. The study, adhering to the Declaration of Helsinki principles, received approval from the Ethics Committee of the Second Affiliated Hospital of Chongqing Medical University [2019(012)], with informed consent from all participants.

### 2.2 Preparation of NECA and Cell Counting Kit-8 assay

**Figure 1:**
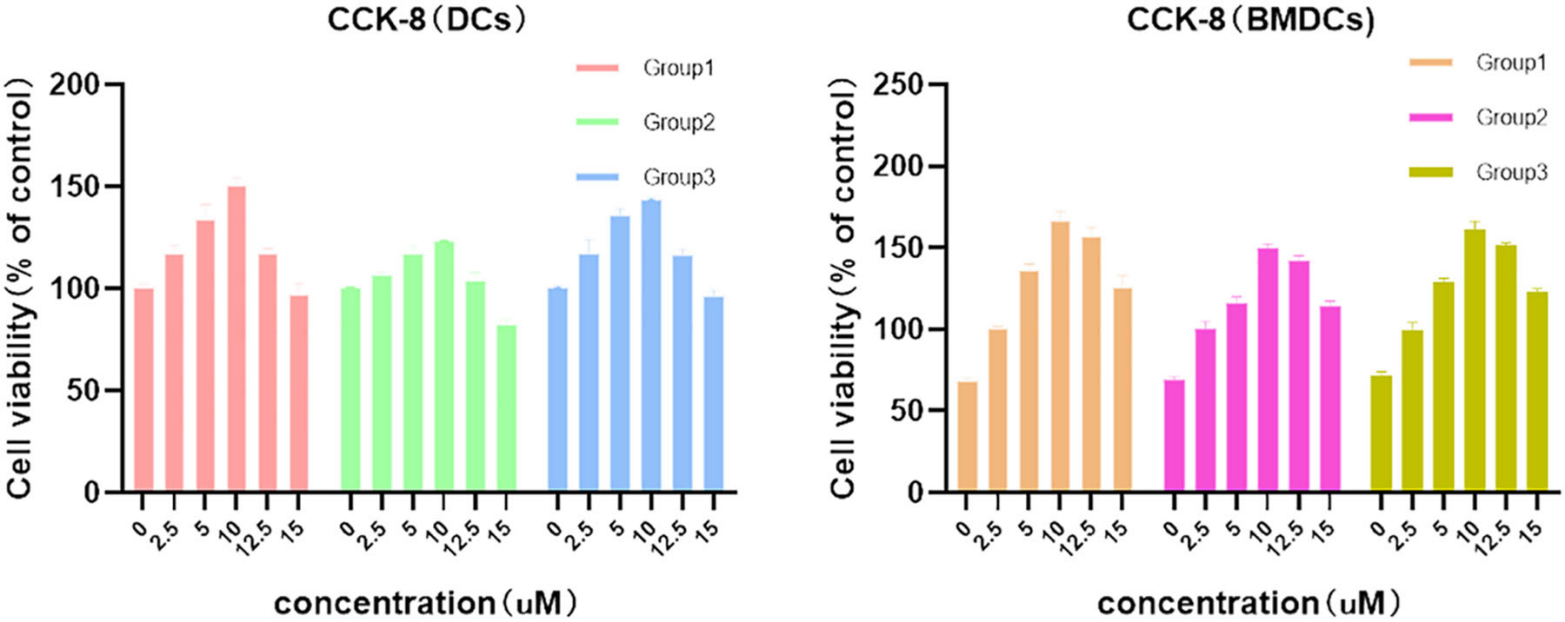
Effects of different concentrations of NECA on DCs and BDMCs cell viability.

NECA, acquired from MedChemExpress (USA), was prepared as a 1 mM stock solution in DMSO for use. In order to determine the NECA concentration in subsequent experiments, different concentrations of NECA were added to DCs and BMDCs for 48 h and incubated with CCK-8 (Beyotime, China) for 4 h, so as to observe the effects of different concentrations of NECA on cell viability. The measurement of optical density at 450 nm was carried out utilizing an ELISA reader from Bio-Rad Laboratories, located in Hercules, CA, USA. Finally, the dose of NECA used for our experiments *in vitro* was 10 µM in DC and BMDC, which was based on CCK-8 assay (**Figure 1**) and previous studies by others.

### 2.3 Cell isolation and culture

The generation of mouse BMDCs was carried out in accordance with the method outlined in a previously established protocol (24). In short, after the C57BL/6 mice were killed by cervical dislocation, their femurs were removed from the ultra-clean experimental table, sterilized, and repeatedly rinsed with sterilized PBS. The bone end was cut off with scissors, the cells were flushed with a 1 mL syringe needle, and the bone marrow suspension was collected in the culture dish. Post-centrifugation, cells were resuspended in RPMI 1640 with additives and treated with GM-CSF and IL-4 for DC maturation. Following incubation at 37°C and 5% CO2, LPS and NECA or vehicle were added on day 10. Cells and medium were harvested after 24 hours for analysis.

The culture of human peripheral blood DC was carried out according to the previous protocol (25–27). Upon receiving informed consent, peripheral blood was collected from patients with DR and DM, as well as from healthy volunteers, into tubes containing EDTA-K2. These blood samples were then diluted with an equivalent volume of sterile PBS in a contamination-free environment and transferred to transparent glass tubes filled with a human lymphocyte separation medium. The samples underwent gradient centrifugation to isolate a layer of white turbid cells, identified as human PBMCs, from the interface of the separation medium and plasma. Following centrifugation, the supernatant was removed, and the PBMCs were isolated. To extract CD14+ monocytes from these PBMCs, human CD14 microbeads (Miltenyi Biotec, Bergisch Gladbach, Germany) were employed. After sterilization and centrifugation, the supernatant was discarded, and the cells were resuspended in RPMI 1640 medium, enhanced with 10% fetal bovine serum and 1% double antibiotic (penicillin/streptomycin). To promote the development and maturity of DCs, GM-CSF (human, 100 ng/mL; AcroBiosystems, Newark, NJ, USA) and IL-4 (human, 50 ng/mL; AcroBiosystems) were added. On the 7th day, 1 μg/mL LPS (Sigma, St. Louis, MO, USA) along with either vehicle or NECA was introduced, and the culture was continued for another 24 hours for subsequent collection of cells and culture medium for further analysis.

Splenocyte collection of mice was performed in line with the procedure outlined in a prior study (28). This established method was also applied for the separation of human peripheral blood and mouse CD4+ T cells of splenic cell suspension using specific CD4 microbeads from Miltenyi Biotec, Bergisch Gladbach, Germany. Following this, DCs or BMDCs treated with NECA (or a vehicle) were co-cultured with either human or mouse CD4+ T cells at a ratio of 1:10 for a duration of five days.

### 2.4 Establishment of diabetic retinopathy mouse model

Chongqing Medical University’s Experimental Animal Center provided 4–5-week-old female C57BL/6 mice for establishing a DR mouse model. These mice underwent a period of acclimatization for one week, followed by a 16-hour fast prior to the induction of the model. Body weights were recorded during the fasting period, and blood glucose levels were measured from the caudal vein. A single intraperitoneal injection of streptozotocin (STZ) at a dosage of 60 mg/kg was administered. Blood glucose levels were then monitored at intervals of 72, 96, and 120 hours. The establishment of type 2 diabetes mellitus was confirmed when blood sugar levels exceeded 16.7 mmol/L on three consecutive measurements, accompanied by symptoms such as polydipsia, polyuria, and weight loss. In this study, the experimental protocol involved administering NECA at a dose of 60 mg/kg by gavage per day to the designated mice in the experimental group, as referenced in (29). This treatment continued for a period of 12 weeks. In contrast, the control group received a daily intake of a saline solution in a volume equal to that of the NECA dosage. The implementation of these animal experiments was carried out in strict compliance with the ethical guidelines sanctioned by the Animal Experiment Committee at Chongqing Medical University.Then, BMDCs were obtained, cultured and differentiated according to the above method (2.3), LPS was added on day 10. Cells and medium were harvested after 24 hours for analysis.

### 2.5 Flow cytometry

In the NECA and control groups, BMDCs or dendritic cells (DCs) were harvested for analysis. These cells underwent staining with several markers: CD40 (fluorescein isothiocyanate [FITC]-conjugated, from Biolegend), CD80 (phycoerythrin [PE]-conjugated, sourced from eBioscience), CD86 (FITC-conjugated from eBioscience, PE-cy7-conjugated from Biolegend), and major histocompatibility complex class II (MHCII) molecules (PE-conjugated, eBioscience). Additionally, human-specific markers were used, including CD40 (FITC-conjugated, Biolegend), CD80 (PE-conjugated, Biolegend), CD86 (PE-cy7-conjugated, Biolegend), and human leukocyte antigen-DR (HLA-DR; PE-cy5.5-conjugated, Biolegend). After a 30-minute incubation at 4°C, the cells were washed, centrifuged, resuspended, and then subjected to flow cytometric analysis.

CD4^+^ T cells, after being co-cultured with DCs or BMDCs, were subjected to a staining process. This process involved the use of several antibodies: for human cells, anti-CD4, anti-CD25, anti-FoxP3, and anti-IL-17 antibodies, all sourced from BioLegend (with anti-CD4 and anti-CD25 labeled with allophycocyanin [APC]) and from eBioscience (with anti-FoxP3 and anti-IL-17 labeled with PE-cy7 and PE, respectively). Similarly, for mouse cells, antibodies against CD4 and CD25 (APC-conjugated) and against FoxP3 and IL-17 (PE-cy7 and PE-conjugated, respectively), all obtained from BioLegend and eBioscience, were used.

### 2.7 Enzyme-linked immunosorbent assay

The levels of IL-6, IL-10, TNF-α, IL-17, and TGF-β in the supernatant of DCs, BMDCs, and co-culture with CD4 were measured by human and mouse multi-set enzymic immunosorbent assay kit (R&D Systems). The levels of IL-12 p70 was measured using mouse IL-12 p70 multi-set enzymic immunosorbent assay kit (R&D Systems) and human IL-12 p70 high-sensitivity ELISA kit (eBioscience).

### 2.8 Hematoxylin and eosin staining

The removed whole eyeballs were embedded after modeling 3 months in paraffin and dehydrated to make 4–6 μm sections. These sections were subsequently stained with H&E using a standard protocol.

### 2.9 Western blotting assay

Following the extraction of proteins from DCs or BMDCs according to established protocols, these proteins were separated using SDS-PAGE and then transferred onto a PVDF membrane. This membrane underwent a blocking process for 2 hours, followed by an overnight incubation at 4°C with the primary antibody. Subsequently, it was subjected to another blocking stage for 1 hour before being incubated with the secondary antibody at ambient temperature. The final step involved the visualization of the membrane through the application of a chemiluminescent reagent.

### 2.10 Real-time qPCR

RNA extraction was carried out using TRIzol reagent from Invitrogen (Carlsbad, CA, USA). The reverse transcription process utilized RT Master Mix for qPCRII and SYBR Green qPCR Master Mix provided by MedChemExpress (USA). Subsequent RT-qPCR analyses were conducted on an ABI Prism 7500 system, a product of Applied Biosystems (CA, USA). For statistical interpretation of the data, the 2^-△△^Ct method was employed. Details of the primer sequences used are listed in **Table 1**.

**Table 1:** Primers used for RT-PCR.

Gene	Forward primer	Reverse primer
Human-TLR4	5’-CCTCCCACTCCAGGTAAGT-3’	5’-GCAGTTTCTGAGCAGTCGT-3’
Human-MYD88	5’-GTGTCCGCACGTTCAAG-3’	5’-CGGTCTCCTCCACATCC-3’
Human-NF-κB	5’-CTGTCCCCATTCTCATCCT-3’	5’-GCCCTTTTCGACTACGC-3’
human-β-actin	5’-GGGCACCGTCTTCTAATTC-3’	5’-GCCTACCATCCTTTGCTG-3’
mice-TLR4	5’-AAGGTGAAAGCAGAAATGTGT-3’	5’-GGGGAGGAAGAAAGGTCTAA-3’
mice-MYD88	5’-CACGAGCCCTTCTTTTCTT-3’	5’-GGGGCATTTCACTGCTT-3’
mice-NF-κB	5’-GTCTCCTCCGCCTTCTG-3’	5’-GGGGTATGCACCGTAACA-3’
mice-β-actin	5’-ATGCCACAGGATTCCATACC-3’	5’-GTGCTATGTTGCTCTAGACTTCG-3’

### 2.11 Statistical analysis

GraphPad Prism 8.0 enabled our data evaluation. Shapiro-Wilk assessed normality. For paired data, the T-test assessed significance between two groups when the pairing difference was normally distributed, otherwise Wilcoxon was used. Significance was noted with mean ± SEM, P < 0.05.

## 3 Results

### 3.1 Effect of NECA on DCs function *in vitro*

**Figure 2:**
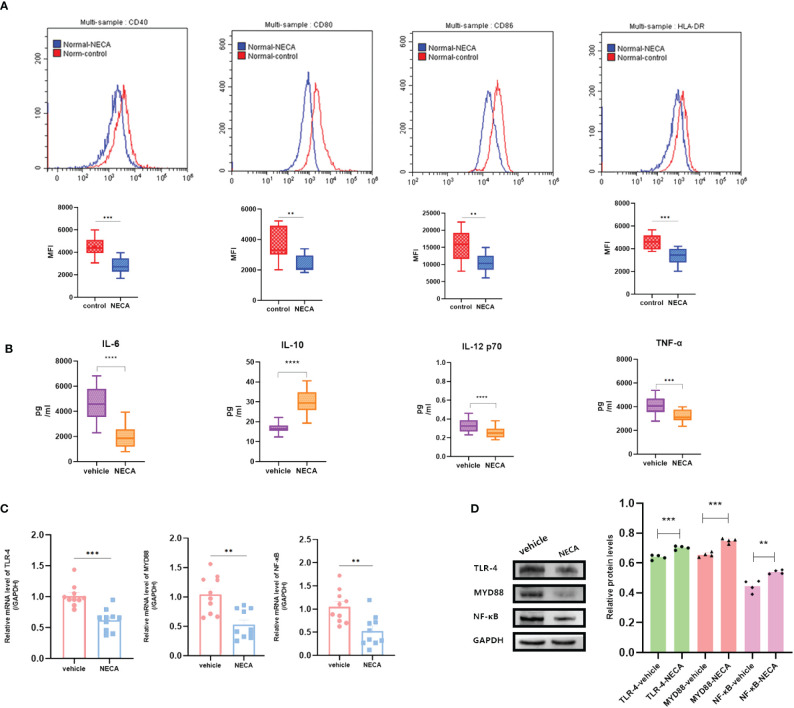
NECA influenced the presence of DC surface markers, release of inflammatory cytokines and mRNA/protein expression in TLR pathway in healthy individuals. **(A)** Histograms were overlapped to compare NECA treated DCs (n = 10) with controls (n = 10) from the representative experiment, and the variation in MFI for surface markers of CD40, CD80, CD86, HLA-DR. **(B)** IL-6, IL-10, IL-12/p70 and TNF-2 were measured by ELISA in NECA-treated DCs (n = 10) vs. vehicles (n = 10). **(C, D)** MRNA relative transcription level and protein expression level of TLR-4, MYD88, and NF-88 in DCs of NECA treated (n = 10) vs. vehicles (n = 10) by PCR and WB. Statistical analysis involved the utilization of the paired samples t-test. (**P < 0.01, ***P < 0.001, ****P < 0.0001).

**Figure 3:**
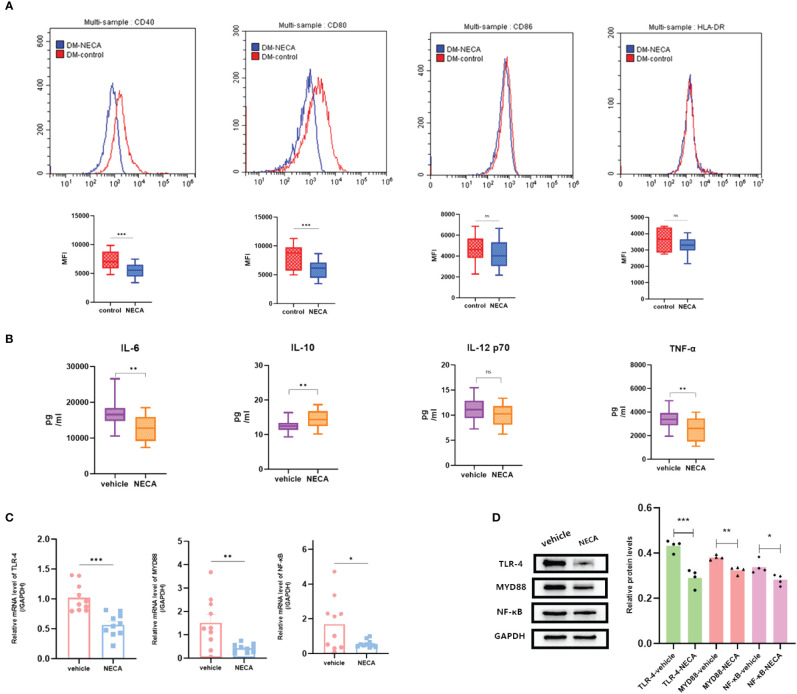
NECA influenced the presence of DC surface markers, release of inflammatory cytokines and mRNA/protein expression in TLR pathway in patients with DM. **(A)** Histograms were overlapped to compare NECA treated DCs (n = 10) with controls (n = 10) from the representative experiment, and the variation in MFI for surface markers of CD40, CD80, CD86, HLA-DR. **(B)** IL-6, IL-10, IL-12/p70 and TNF-2 were measured by ELISA in NECA-treated DCs (n = 10) vs. vehicles (n = 10). **(C, D)** MRNA relative transcription level and protein expression level of TLR-4, MYD88, and NF-88 in DCs of NECA treated (n = 10) vs. vehicles (n = 10) by PCR and WB. Statistical analysis involved the utilization of the paired samples t-test. (ns: not significant, *P < 0.05, **P < 0.01, ***P < 0.001).

**Figure 4:**
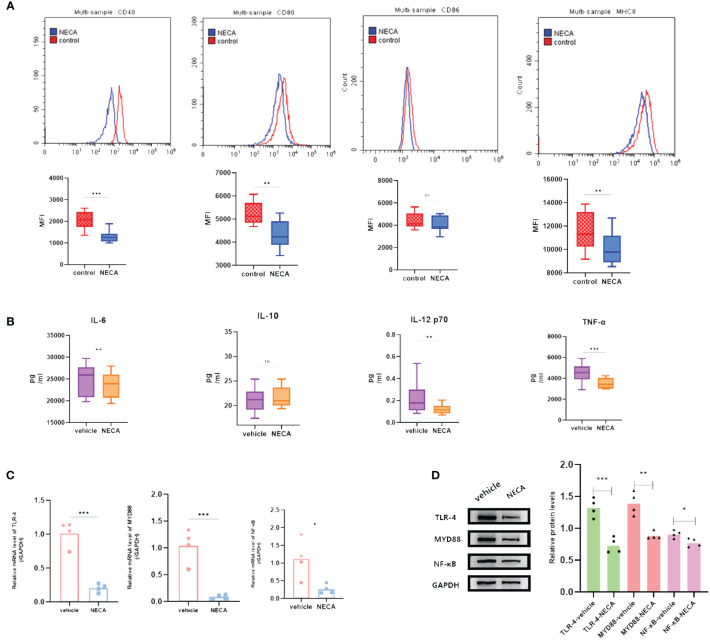
NECA influenced the presence of BMDC surface markers, release of inflammatory cytokines and mRNA/protein expression in TLR pathway in mice. **(A)** Histograms were overlapped to compare NECA treated BMDCs (n = 4) with controls (n = 4) from the representative experiment, and the variation in MFI for surface markers of CD40, CD80, CD86, MHCII. **(B)** IL-6, IL-10, IL-12/p70 and TNF-2 were measured by ELISA in NECA-treated BMDCs (n = 4) vs. vehicles (n = 4). **(C, D)** MRNA relative transcription level and protein expression level of TLR-4, MYD88, and NF-88 in BMDCs of NECA-treated (n = 4) vs. vehicles (n = 4) by PCR and WB. Statistical analysis involved the utilization of the paired samples t-test and Wilcoxon. (ns: not significant, *P < 0.05, **P < 0.01, ***P < 0.001).

**Figure 5:**
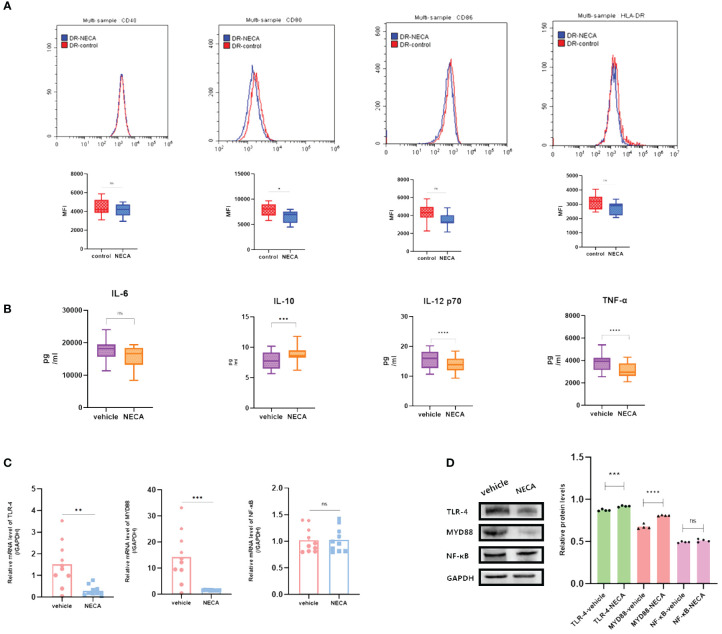
NECA influenced the presence of DC surface markers, release of inflammatory cytokines and mRNA/protein expression in TLR pathway in patients with DR. **(A)** Histograms were overlapped to compare NECA treated DCs (n = 10) with controls (n = 10) from the representative experiment, and the variation in MFI for surface markers of CD40, CD80, CD86, HLA-DR. **(B)** IL-6, IL-10, IL-12/p70 and TNF-2 were measured by ELISA in NECA-treated DCs (n = 10) vs. vehicles (n = 10). **(C, D)** MRNA relative transcription level and protein expression level of TLR-4, MYD88, and NF-88 in DCs of NECA-treated (n = 10) vs. vehicles (n = 10) by PCR and WB. Statistical analysis involved the utilization of the paired samples t-test and Wilcoxon test. (ns: not significant, *P < 0.05, **P < 0.01, ***P < 0.001,****P < 0.0001).

**Figure 6:**
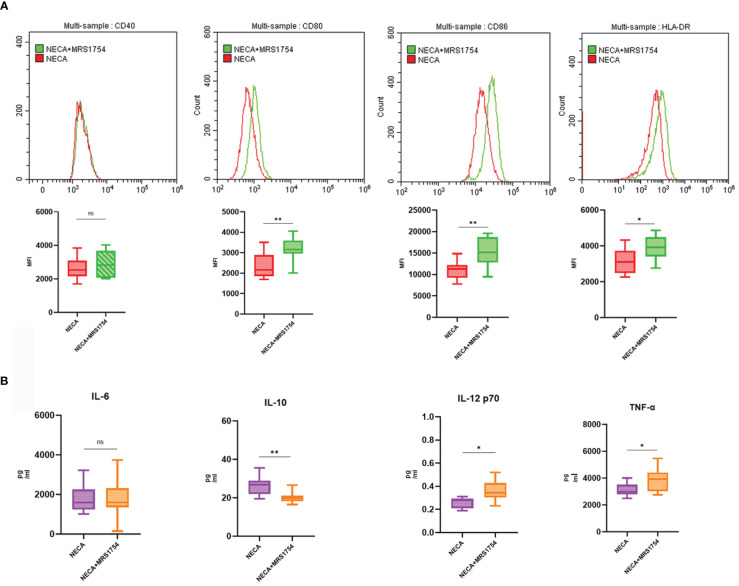
MRS1754 inhibited the effect of NECA on DC surface markers and release of inflammatory cytokines in healthy individuals. **(A)** Histograms were overlapped to compare NECA treated DCs (n = 10) with NECA+MRS1754-treated DCs (n = 10) from the representative experiment, and the variation in MFI for surface markers of CD40, CD80, CD86, HLA-DR. **(B)** IL-6, IL-10, IL-12/p70 and TNF-2 were measured by ELISA in NECA-treated DCs (n = 8) vs. NECA+MRS1754-treated DCs (n = 8). Statistical analysis involved the utilization of the paired samples t-test and Wilcoxon test. (ns: not significant, *P < 0.05, **P < 0.01).

DCs are vital for immune responses (30, 31). Previous studies have shown that adenosine and its receptor agonist NECA can reduce inflammation in diabetic rats (32). A multitude of studies have indicated the involvement of the immune system in DR progression (33). Hence, to investigate the impact of NECA on DCs in a laboratory setting, we examined both BMDCs and DCs from patients with DM and DR, as well as individuals without any health conditions. FCM was used to assess surface markers, which based on previous studies [27.28.29], such as CD80, CD86, CD40, HLA-DR, or MHC-II, on DCs and BMDCs. NECA was observed to significantly inhibit the presence of CD40, CD80, CD86, and HLA-DR in DCs from healthy subjects (**Figure 2A**). It inhibited the expression of surface markers CD40, CD80, and MHC-II in patients with DM DCs (**Figure 3A**) and BMDCs (**Figure 4A**) but only suppressed the presence of surface marker CD80 in patients with DR DCs (**Figure 5A**). However, after adding A2B receptor antagonist MRS1754 (10 µM),the inhibitory effect of NECA on the expression of CD80, CD86, and HLA-DR (**Figure 6A**), which are surface markers of DCs in healthy individuals, was reduced.

In order to study the impact of NECA on the release of cytokines by DCs and BMDCs, the levels of IL-6, IL-10, IL-12 p70, and TNF-α were assessed using ELISA. NECA increased the IL-10 secretion in healthy individuals (**Figure 2B**), patients with DM (**Figure 3B**) and DR (**Figure 5B**) while inhibiting the secretion of IL-6, IL-12 p70, and TNF-α in healthy individuals (**Figure 2B**). Furthermore, NECA suppressed TNF-α secretion in DCs of mice (**Figure 4B**), patients with DM (**Figure 3B**) and DR (**Figure 5B**), but only suppressed IL-12 p70 secretion in mice DCs (**Figure 4B**) and IL-6 secretion in DCs of patients with DM (**Figure 3B**). The introduction of MRS1754 led to an increase in IL-12 p70 and TNF-α production and a decrease in IL-10 levels in the DCs supernatant from healthy individuals (**Figure 6B**).

Our results showed that NECA had a statistically significant effect on the BMDCs and DCs of healthy individuals, patients with DM and DR.

### 3.2 Effect of NECA on TLRs pathway in DCs and BMDCs *in vitro*

A number of studies have reported that TLRs on the surface of DC may influence inflammation through the adaptor protein MyD88 (11, 34, 35). Therefore, we studied the effect of NECA on the TLR pathway of DCs in healthy individuals, patients with DM and DR, and mice. In this study, NECA inhibited the relative mRNA transcription level of TLR4 and MYD88 and the expression of TLR4 and MYD88 proteins in DCs of healthy individuals (**Figures 2C, D**), patients with DM (**Figures 3C, D**) and DR (**Figures 5C, D**), and BMDCs (**Figures 4C, D**). NECA inhibited the relative mRNA transcription level of NF-κB and expression of NF-κB protein in DCs of healthy individuals (**Figures 2C, D**), patients with DM (**Figures 3C, D**), and BMDCs (**Figures 4C, D**). All the differences were statistically significant.

### 3.3 Effect of NECA on DCs function *in vivo*

**Figure 7:**
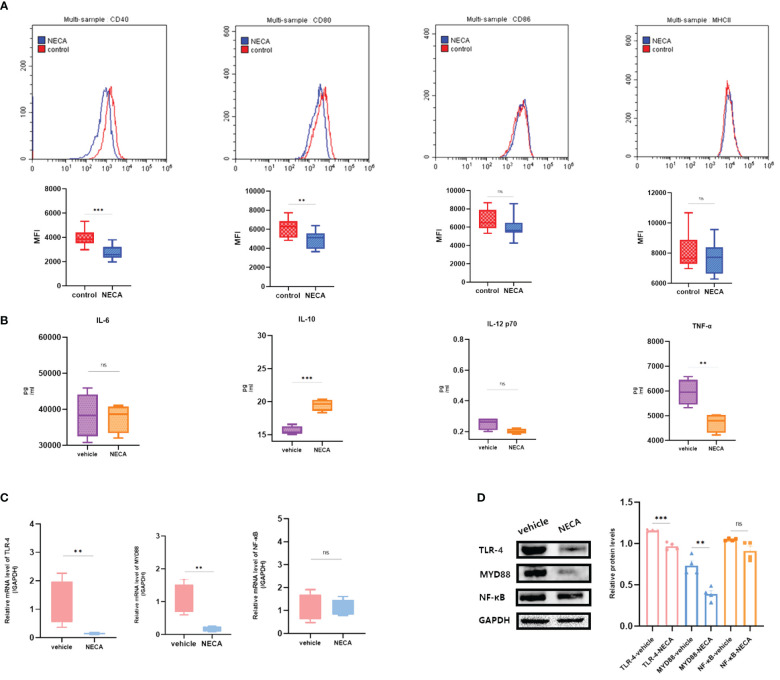
Effect of NECA on BMDCs function *in vivo* in mice. **(A)** Histograms were overlapped to compare NECA treated BMDCs (n = 4) with controls (n = 4) from the representative experiment, and the variation in MFI for surface markers of CD40, CD80, CD86, MHCII in EDR mice. **(B)** IL-6, IL-10, IL-12/p70 and TNF-2 were measured by ELISA in NECA-treated BMDCs (n = 4) vs. vehicles (n = 4). **(C, D)** MRNA relative transcription level and protein expression level of TLR-4, MYD88, and NF-88 in BMDCs of NECA-treated (n = 4) vs. vehicles (n = 4) by PCR and WB. Statistical analysis involved the utilization of the paired samples t-test. (ns: not significant, **P < 0.01, ***P < 0.001, ****P < 0.0001).

**Figure 8:**
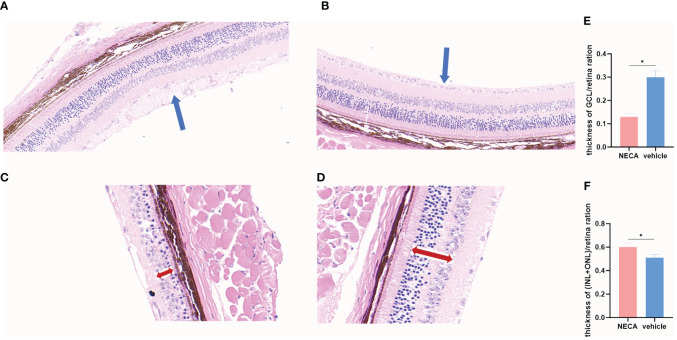
Changes of retina by HE staining in EDR mice. **(A)** Changes of GCL in saline-treated EDR mice. **(B)** Changes of GCL NECA-treated EDR mice. **(C)** Changes of INL and ONL in saline-treated EDR mice. **(D)** Changes of INL and ONL in NECA-treated EDR mice. **(E)** Thickness of GCL/retina ration in NECA-treated EDR mice (n=4) vs. saline-treated EDR mice (n=4). **(F)** Thickness of (INL+ONL)/retina ration in NECA-treated EDR mice (n=4) vs. saline-treated EDR mice (n=4). (The blue arrow points to the ganglion cells layer,the red double-headed arrow points to the inner and outer nuclear layers). Statistical analysis involved the utilization of the paired samples t-test. (*P < 0.05).

*In vivo* experiments demonstrated that NECA reduced the expression of CD40 and CD80 surface markers on DCs in STZ-induced EDR mice (**Figure 7A**). Moreover, NECA decreased TNF-α secretion while increasing IL-10 secretion in these mice (**Figure 7B**). Additionally, NECA downregulated both mRNA transcription and protein expression levels of TLR4 and MYD88 in the DCs of EDR mice (**Figures 7C, D**). The ganglion cells layer (GCL) displayed obvious vacuolar degeneration (**Figure 8A**), and cell density and thickness in the inner nuclear layers (INL) and outer nuclear layers (ONL) was reduced in EDR mice (**Figure 8C**). After NECA treatment, the structure of each layer of retinal tissues was regular in EDR mice without ganglion vacuolar degeneration (**Figure 8B**), without inner and outer nuclear layers (**Figure 8D**) were reduced. The differences between thickness of GCL/retina ration in NECA-treated EDR mice and saline-treated EDR mice were statistically significant (**Figure 8E**). The same statistical differences existed between thickness of (INL+OPL)/retina ration in NECA-treated EDR mice and saline-treated EDR mice (**Figure 8F**). Our results showed that NECA alleviated changes of diabetic retinopathy in mice in HE staining.

### 3.4 Effect of Th17 and Treg cells differentiation co-cultured with NECA-treated DCs and BMDCs *in vitro*

Thl7 and Treg cells are vital in inflammatory response (36–39). DCs can significantly influence T-cell differentiation (40, 41). Previous studies have revealed that Treg/Th17 cells affect the inflammatory response of diabetic patients (42). This research aimed to determine if DCs and BMDCs stimulated by NECA could influence the differentiation of Th17 and Treg cells and the secretion of related cytokines.

**Figure 9:**
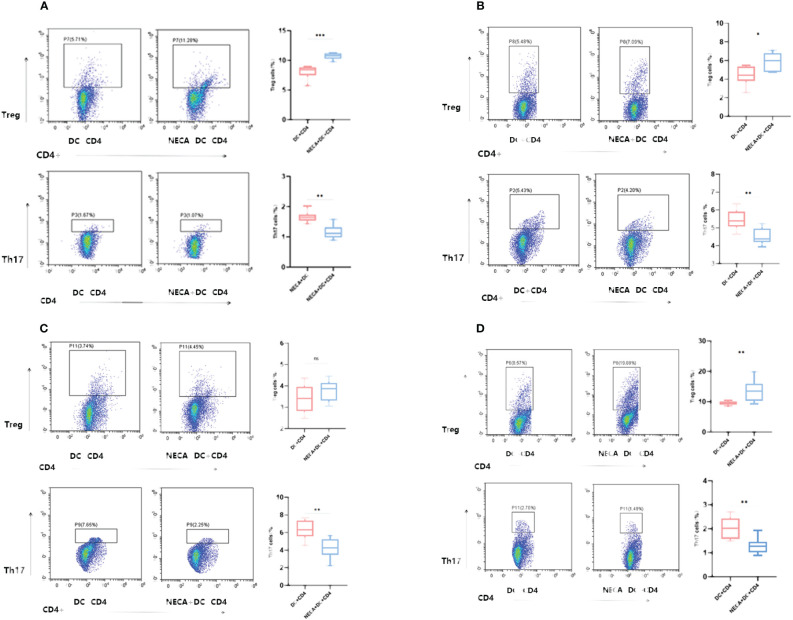
NECA-treated DCs/BMDCs promoted the differentiation of Treg cells and inhibited the differentiation of Th17 cells. **(A–D)** Proportion of Treg and Th17 cells in CD4 after co-culture of NECA-treated DC and CD4 cells (n=8) vs. co-culture of NECA-untreated DC and CD4 cells (n=8) detected by FCM in healthy individuals/DM patients/DR patients/mice. The statistical analysis utilized the paired samples t-test. (ns: not significant, *P < 0.05, **P < 0.01, ***P < 0.001).

**Figure 10:**
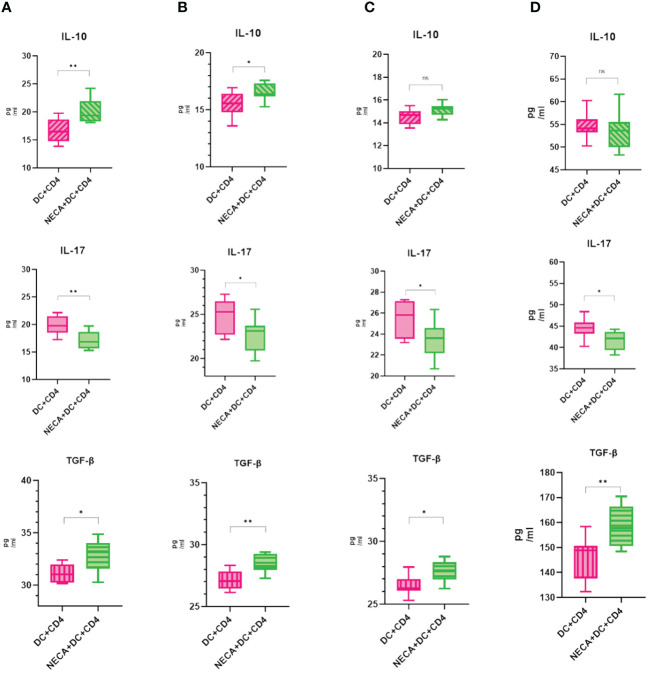
NECA-treated DC affects cytokines in the Treg and Th17 cells. **(A–D)** ELISA analyzed the secretion of IL-10, IL-17, TGF-β after co-culture of NECA-treated DC and CD4 cells (n=8) vs. co-culture of NECA-untreated DC and CD4 cells (n=8) in healthy individuals/DM patients/DR patients/mice. The statistical analysis involved the application of the paired samples t-test. (*P < 0.05, **P < 0.01).

For this purpose, NECA-activated BMDCs and DCs, sourced from both healthy individuals and patients with DM and DR, were co-cultured with CD4+ T cells isolated from naïve mice and humans. The findings revealed that BMDCs and DCs treated with NECA in both healthy subjects and patients suffering from DM and DR, suppressed Th17 cell differentiation. Conversely, these NECA-treated cells promoted the differentiation of Treg cells in mice, healthy subjects, and patients with DM, as identified by FCM analysis, illustrated in **Figure 9**. ELISA showed that NECA-treated DCs inhibited the IL-17 secretion, whereas stimulated the TGF-β and IL-10 secretion in healthy individuals and patients with DM. It inhibited the IL-17 secretion but increased the TGF-β secretion in patients with DR and mice (**Figure 10**).

## 4 Discussion

DR is a microvascular complication of diabetes. DR pathogenesis is complex. Inflammation is the main cause of all diabetic complications, including DR (43). The continuous rise in blood sugar in diabetic patients induces low-grade inflammation, resulting in leukocyte stasis and retinal microvascular changes (44). This can lead to retinal inflammation, oxidative stress, and rupture of the blood-retinal barrier. Chronic retinal inflammation causes capillary degeneration, non-perfusion, vascular permeability, and leakage, while vascular injury initiation causes retinal neovascularization, proliferative diabetic retinopathy, and vision loss (45). In our previous study, we found that people with DR had less adenosine in their stools than healthy individuals (22). Previous research has shown that adenosine regulates immunity. Adenosine analogs have protective effects in many inflammatory disease models. Adenosine deficiency contributes to chronic inflammation in several autoimmune diseases (16). We aimed to understand whether the decrease in adenosine in the gut of patients with DR affects the occurrence and development of the disease.

NECA is a non-selective adenosine receptor agonist. In this study, we showed that *in vitro*, NECA decreased the expression of surface markers CD40 and CD80 in mouse BMDCs and DCs of patients with DM and healthy individuals and the expression of MHCII in BMDCs and HLA-DR in DCs of healthy individuals. Only CD80 expression was inhibited in DCs of patients with DR. NECA increased the cytokine IL-10 secretion in DCs of patients with DM and DR and healthy individuals. However, NECA inhibited the secretion of DCs cytokines IL-6, IL-12 p70, and TNF-α in healthy individuals. Moreover, NECA inhibited the secretion of IL-12 p70 and TNF-α from BMDCs while inhibiting the secretion of IL-12 p70 and TNF-α in patients with DM and DR, respectively. Novitskiy has shown that adenosine stimulates intracellular cAMP accumulation through Gs-coupled adenosine receptors A2A and A2B and regulates the differentiation and function of mouse and human macrophages and DCs (46). Alessandra Franco et al. found that tmDCs in children secrete the anti-inflammatory factor IL-10 when stimulated by Fc and overexpress the adenosine A2AR, which, when activated by NECA, inhibits the release of pro-inflammatory cytokines by most immune cells (47). Jeffrey M. Wilson described that NECA inhibits TNF-α and IL-12 in DCs and BMDCs in a concentration-dependent manner but increases IL-10 secretion, which is regulated by the A2BR (48). Liang Dongchun reported that BMDC differentiated into CD11c^+^Gr-1^+^, which enhanced the stimulation of Th17 in the medium containing NECA, and γδ-T cells are necessary for this enhanced reaction process. Many studies are consistent with our findings (49).

Four types of adenosine receptors (A1R, A2AR, A2BR, and A3R) exert negative or positive effects by binding to different receptors. Activation of A2AR suppresses the response (50), while activation of A2BR enhances it (47). However, many studies have suggested that A2BR is involved in the immune response to inflammation and plays an anti-inflammatory role (51). For example, during hypoxia and peritonitis, mucosal inflammation is reduced by A2BR signaling (52), while drug blocking of A2BR enhances septicemia-induced inflammation and angioplastication-induced vascular disease (53). In a mouse gut IR model, A2BR knockout mice had more severe IR damage than wild-type mice (54). In animal models of IR injury, adenosine protects intestinal microcirculation through A2BR signaling (55). NECA is one of the most potent A(2B) adenosine receptor agonists. Németh Zoltán H found that NECA could prevent the development of diabetes in STZ and cyclolinamide-induced diabetic mouse models, strongly suppressed expression of the pro-inflammatory cytokines TNF-α, macrophage inflammatory protein 1, IL-12, and IFN-γ. At the same time, A2B receptor antagonists could reverse the effect of NECA (56). Different studies suggest that the anti-inflammatory effect may be caused by A2AR (57). However, most studies believe that the effect is mainly through A2BR and A3 stimulation, which may be related to NECA concentrations. Dal Ben Diego et al. reported that different concentrations of NECA derivatives could inhibit the activity of adenylyl cyclase, thereby affecting its stimulating effect on A3R (58). Our results showed that NECA inhibited the maturation of DCs and BMDCs, increased the production of anti-inflammatory factors, decreased pro-inflammatory factors, and played a role in inhibiting inflammation. Furthermore, we added MRS1754, an antagonist of the A2BR, to the DC of healthy individuals, and the effect of NECA on DC changed. Hence, We concluded that NECA inhibited inflammation not by activating A2AR, but perhaps by A2BR or the other types of adenosine receptors (A1R or A3R).A2BR may play an anti-inflammatory role.

The data reported herein also suggests that different populations of DC and BMDC have different responses to NECA. This phenomenon may reflect the existence of unique DC subtypes or indicate that DC from different sources was in different stages of maturity before NECA treatment, subtypes on the different expression of adenosine receptors in DC, thus causing different responses. The expression of adenosine receptors on the DC surface of patients with DR is also a direction that we will continue to study in the future. However, our findings suggest that NECA can inhibit inflammation *in vitro* and *in vivo*. Many previous studies have investigated the role of NECA in diabetes and the complications caused by diabetes. Studies have been conducted on heart disease, diabetes, and kidney disease (59), but few studies have been conducted on NECA in DR. Our previous studies found adenosine changes through intestinal metabolites in patients with DR. In this study, intragastric administration of NECA reduced the intraocular retinal lesions, inhibited DC maturation and the release of inflammatory cytokines through the TLR-MYD88-NF-κB pathway in animals. This suggested that NECA may reduce inflammation and slow down the development of DR *in vivo*. Adenosine regulates immune homeostasis. The decrease in adenosine in the feces of DR patients may destroy the regulation of adenosine on immunity, increase the immune response, and accelerate the disease progression of DR. However, our findings are only a preliminary study, and there is no direct evidence yet on how NECA’s effect on BMDCs can reduce retinopathy through intraocular responses. For the insufficiency of the experiment, we need further exploring in our subsequent experiments.

Moreover, we found that mRNA expression related to the TLR-MYD88-NF-κB pathway in DC was also altered by PCR and WB detection. It was previously reported that A2a mainly activates the downstream signaling pathway through the PKA pathway and may directly or indirectly cause changes in nuclear factors through cAMP phosphorylation (60). Additionally, this might be attributed to alterations in potassium channel activity, as evidenced by 86Rb+ efflux experiments, or due to the suppression of calcium channels, indicated by 45Ca2+ uptake experiments. TLRs are sentinel cell transmembrane pattern recognition receptors expressed in human and other mammalian white blood cells. The involvement of TLRs leads to the initiation of signaling cascades that ultimately lead to host immune responses. Activation of the TLR signaling pathway can cause the onset of immune diseases (61). The findings from our study indicated that NECA significantly reduced both the mRNA transcription levels and the protein expression of TLR4 and MYD88 in BMDCs and DCs derived from healthy individuals, as well as in those from patients with DM. In addition, similar inhibitory effects of NECA on the mRNA transcription and protein expression of TLR4 and MYD88 were observed in DCs from DR patients. In summary, our study revealed that NECA reduces the mRNA of the TLR-MYD88-NF-κB pathway, affecting downstream cAMP changes after adenosine receptor binding, suggesting that this pathway may also affect the inflammatory effect of DC.

Thl7 and Treg cells are important in promoting or inhibiting the inflammatory response. Breaking the pro-inflammatory balance between Thl7 and inhibitory Treg cells causes many inflammatory and autoimmune diseases (36). An imbalance between these two cell types can precipitate immune disorders, including ulcerative colitis (UC) (37) and type 2 diabetes mellitus (38). Treg cells are integral in suppressing effector T cells, fostering immune tolerance, and averting autoimmune conditions. Conversely, Th17 cells are implicated in various autoimmune diseases. Under normal physiological conditions, these cell types mutually inhibit each other, sustaining a dynamic equilibrium that is vital for immune defense and stability (39). Th17 cells emerge from DCs within an environment rich in pro-inflammatory cytokines. Enhancing specific DC subsets and curtailing the production of pro-inflammatory cytokines has been shown to promote Treg cell proliferation and impede Th17 cell differentiation (40). Furthermore, hindering the maturation of dendritic cells and the production of IL-6 can significantly influence T-cell differentiation (41). In our study, we examined CD4+ T cell differentiation after NECA processing *in vitro*, which promotes Treg cell differentiation and TGF-β production in the analysis of the inhibitory factors of the Treg cells while inhibiting T17 cell differentiation and inflammatory factor production. Further research is needed to determine if NECA directly impacts T-cell differentiation. In general, our study found that NECA can inhibit inflammation by affecting DC.

In conclusion, there are few studies on the correlation between intestinal fecal metabolites and DR patients and none on the correlation between adenosine and gut DR. Our research indicated that the adenosine receptor ligand may treat DR.

## Data availability statement

The original contributions presented in the study are included in the article/**Supplementary Material**. Further inquiries can be directed to the corresponding authors.

## Ethics statement

The studies involving humans were approved by Second Affiliated Hospital of Chongqing Medical University. The studies were conducted in accordance with the local legislation and institutional requirements. The participants provided their written informed consent to participate in this study. The animal study was approved by Second Affiliated Hospital of Chongqing Medical University. The study was conducted in accordance with the local legislation and institutional requirements.

## Author contributions

LL: Writing – review & editing, Writing – original draft, Visualization, Validation, Software, Resources, Project administration, Methodology, Investigation, Formal analysis, Data curation, Conceptualization. JC: Writing – original draft, Software, Methodology, Formal analysis. ZW: Writing – review & editing, Software, Resources, Methodology, Investigation, Data curation. YX: Writing – review & editing, Supervision, Resources, Data curation. HY: Writing – review & editing, Software, Methodology, Formal analysis. WL: Writing – review & editing, Software, Resources. XZ: Writing – review & editing, Writing – original draft, Visualization, Validation, Supervision, Resources, Project administration, Methodology, Investigation, Funding acquisition, Formal analysis, Data curation, Conceptualization. MZ: Writing – review & editing, Writing – original draft, Visualization, Validation, Supervision, Software, Resources, Project administration, Methodology, Investigation, Funding acquisition, Formal analysis, Data curation, Conceptualization.
