# Supplementary figures and images for: NECA alleviates inflammatory responses in diabetic retinopathy through dendritic cell toll-like receptor signaling pathway
Source: Front Immunol. 2024 Jun 4;15:1415004. doi: 10.3389/fimmu.2024.1415004 (PMC11182989; doi:10.3389/fimmu.2024.1415004)

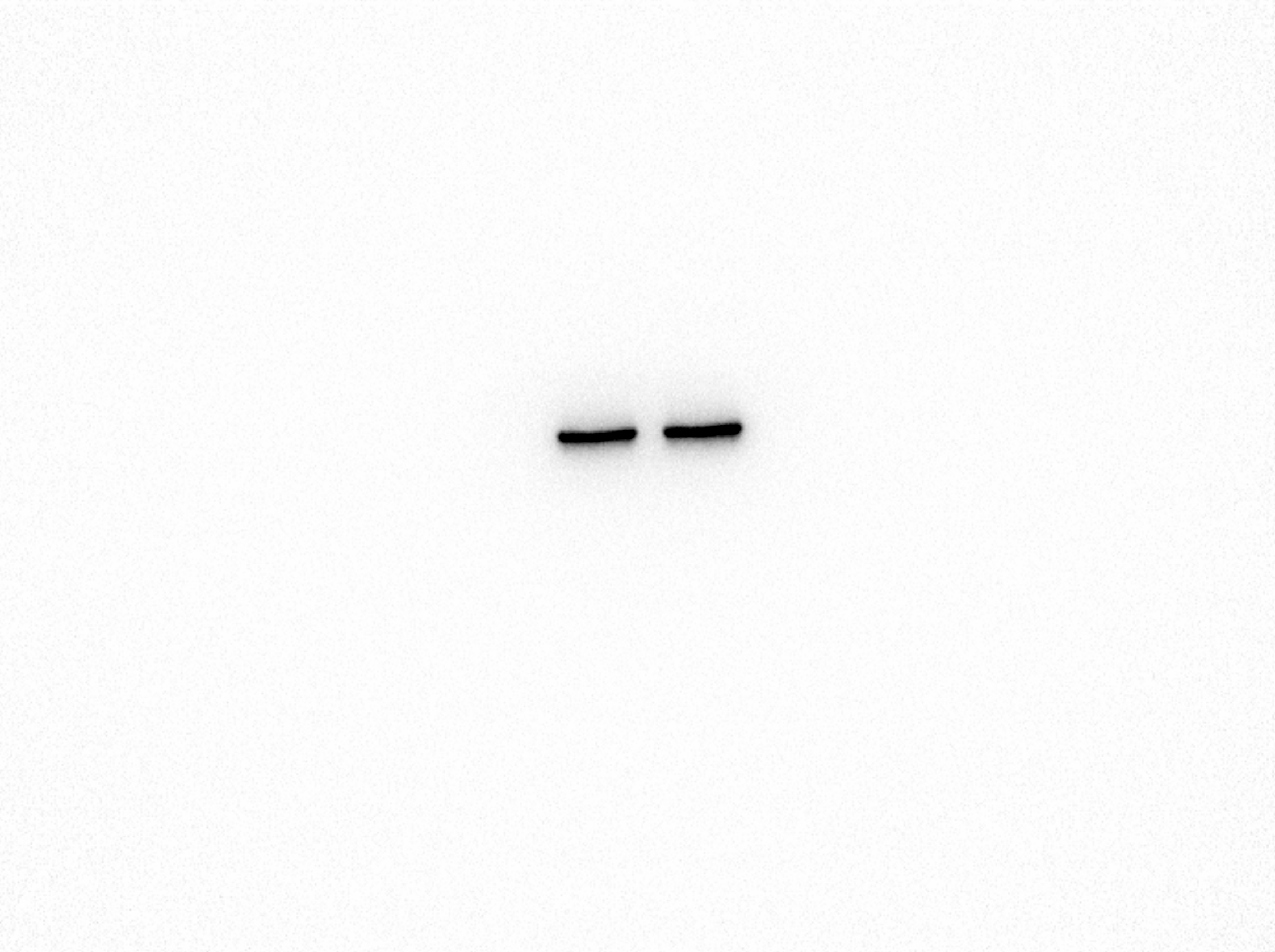

Supplement: Supplementary file 7 [file DataSheet_7.zip › GAPDH/Fig.2.2.tif]

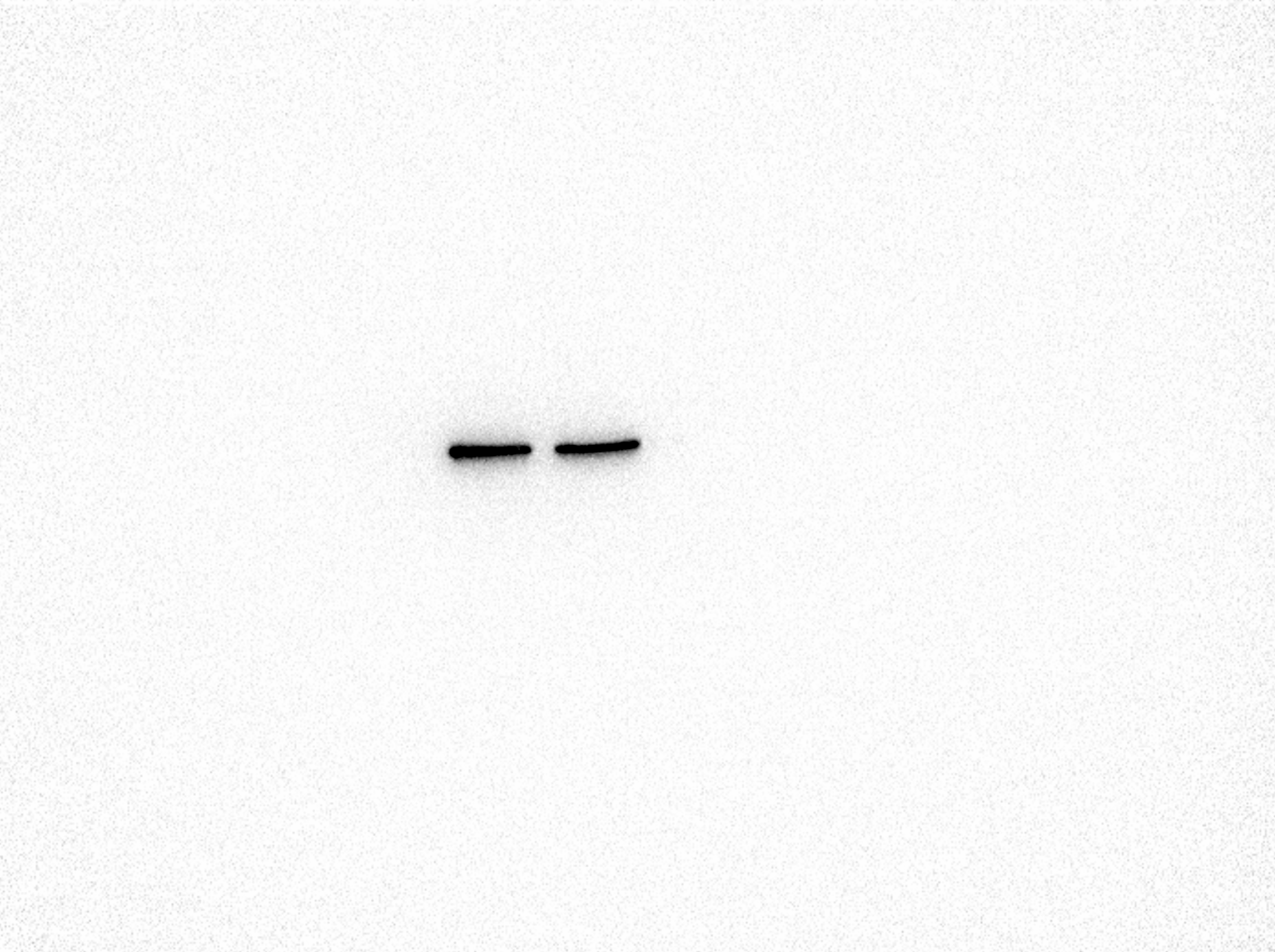

Supplement: Supplementary file 7 [file DataSheet_7.zip › GAPDH/Fig.2.3.tif]

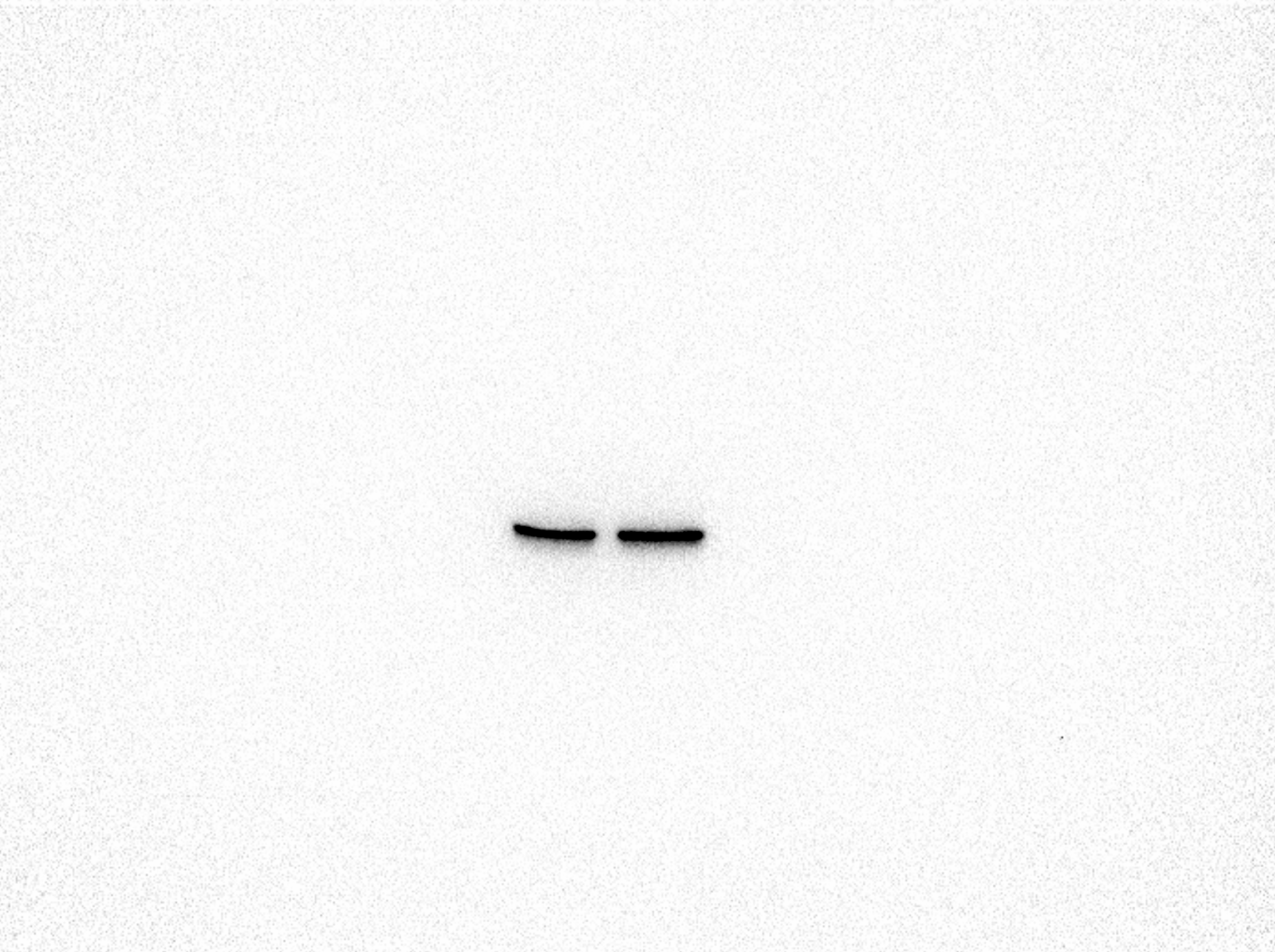

Supplement: Supplementary file 7 [file DataSheet_7.zip › GAPDH/Fig.2.4.tif]

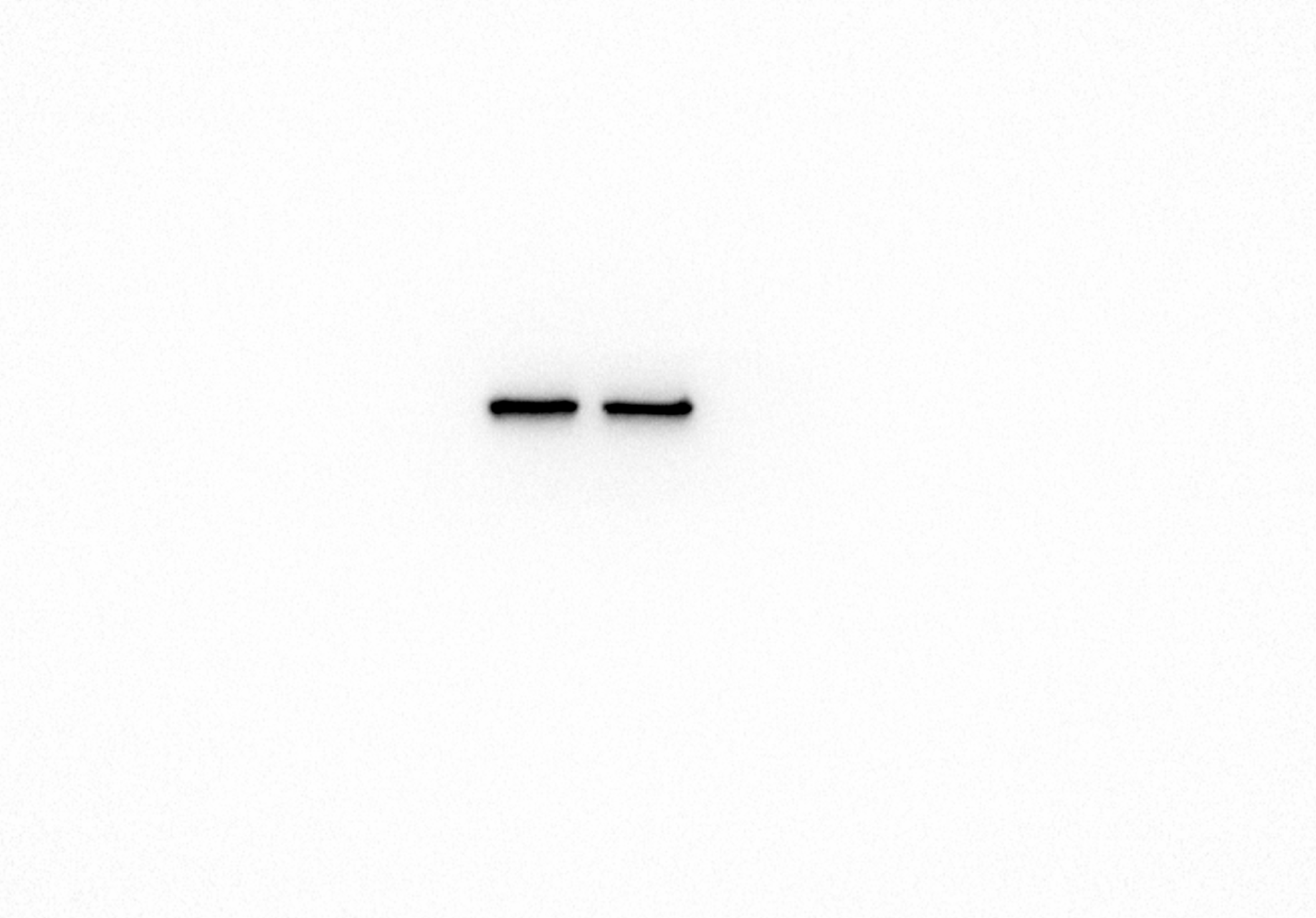

Supplement: Supplementary file 7 [file DataSheet_7.zip › GAPDH/Fig.3.1.tif]

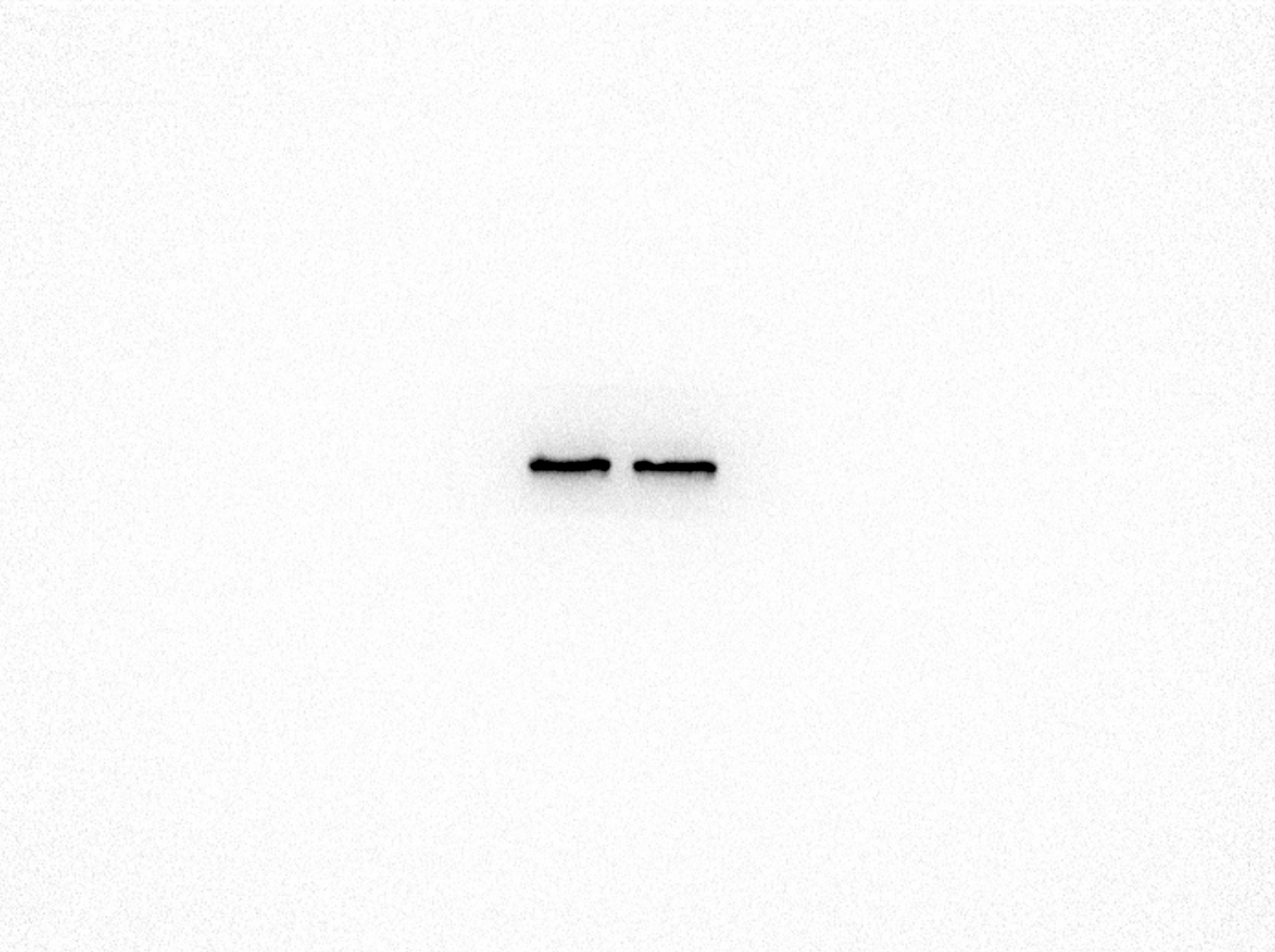

Supplement: Supplementary file 7 [file DataSheet_7.zip › GAPDH/Fig.3.2.tif]

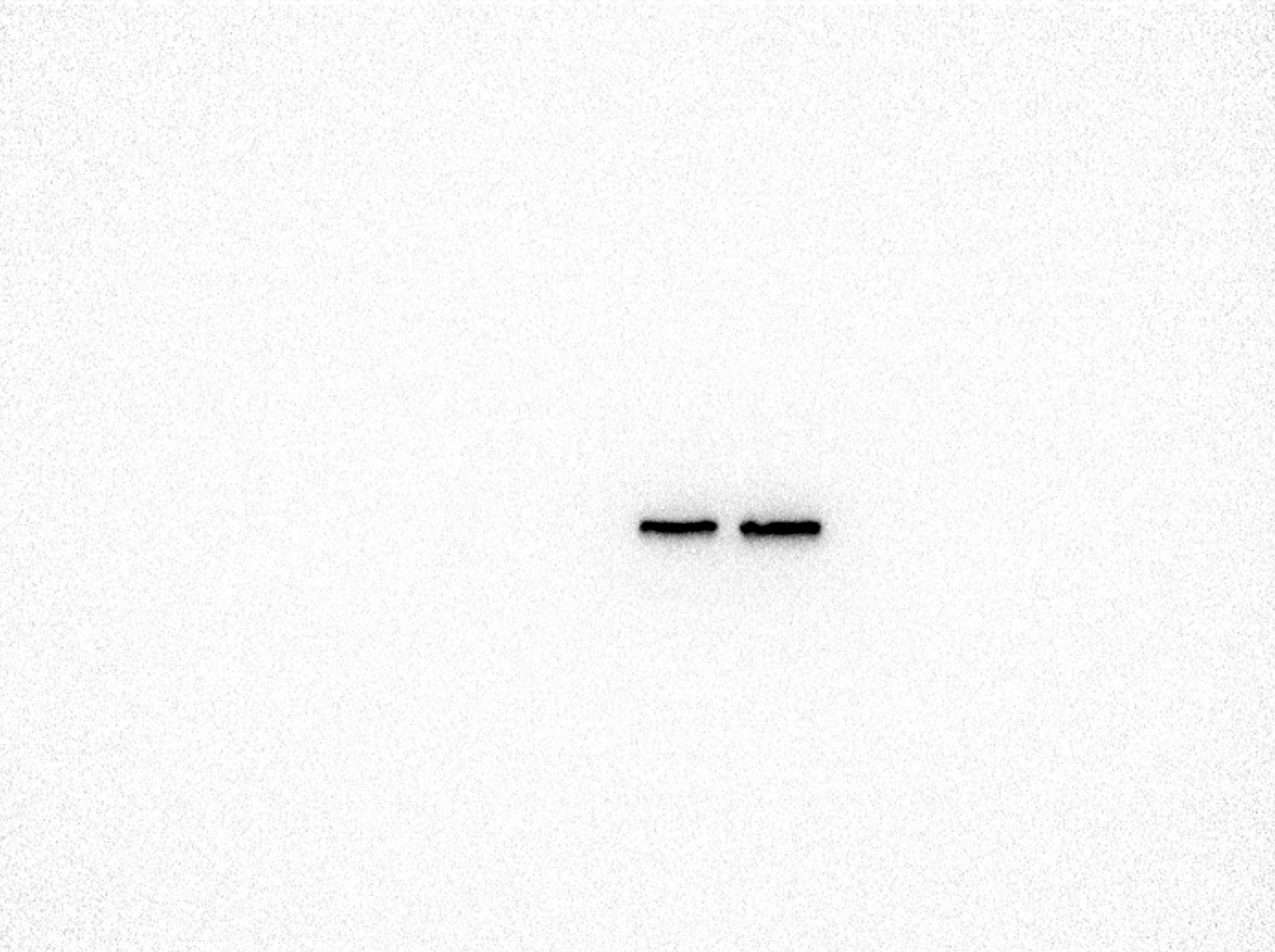

Supplement: Supplementary file 7 [file DataSheet_7.zip › GAPDH/Fig.4.2.tif]

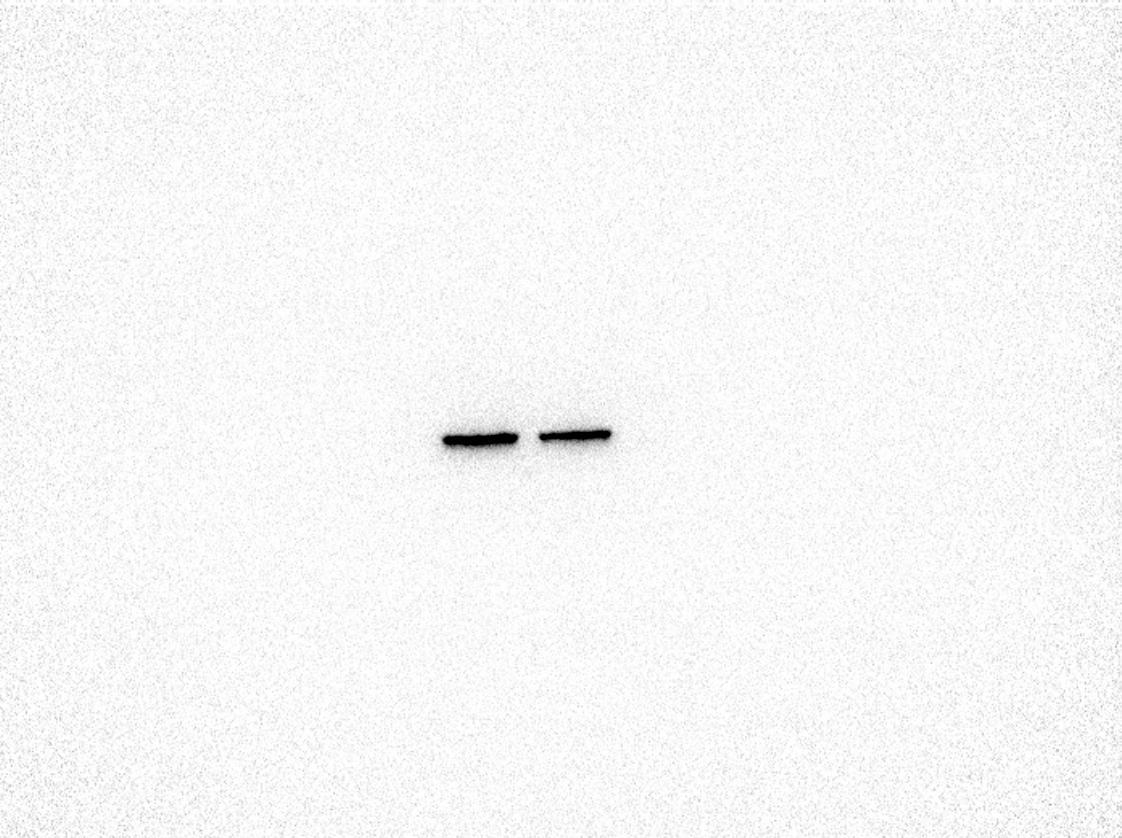

Supplement: Supplementary file 7 [file DataSheet_7.zip › GAPDH/Fig.4.3.tif]

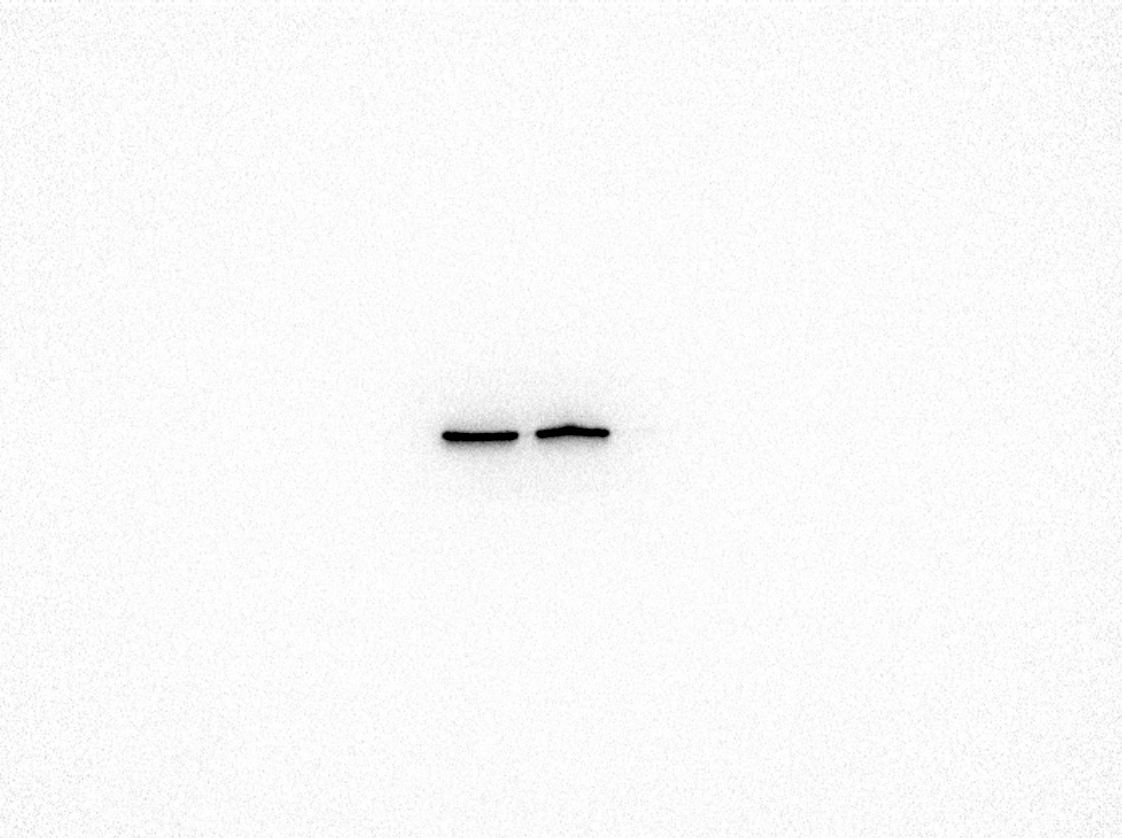

Supplement: Supplementary file 7 [file DataSheet_7.zip › GAPDH/Fig.4.4.tif]

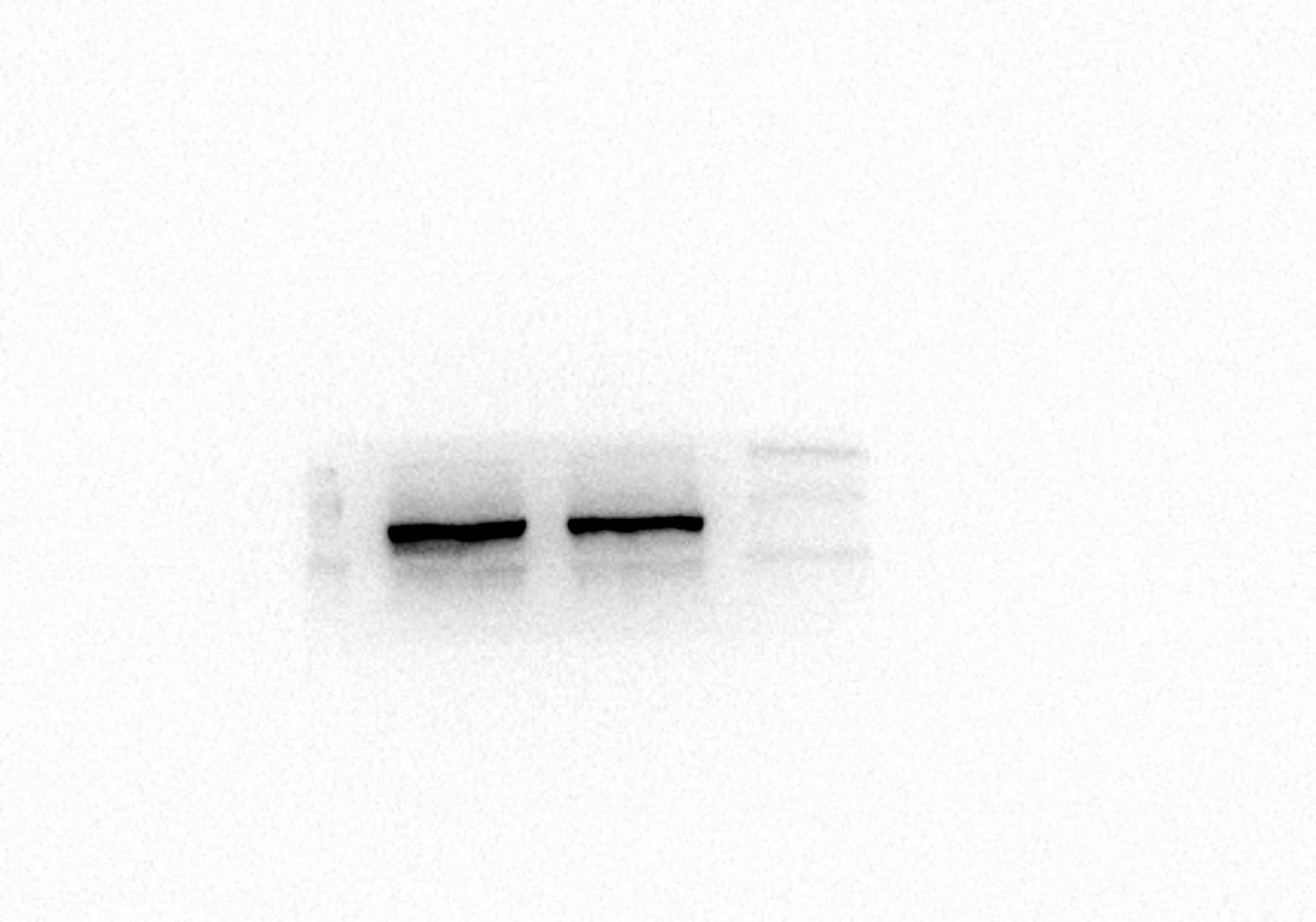

Supplement: Supplementary file 7 [file DataSheet_7.zip › GAPDH/Fig.5.1.Tif]

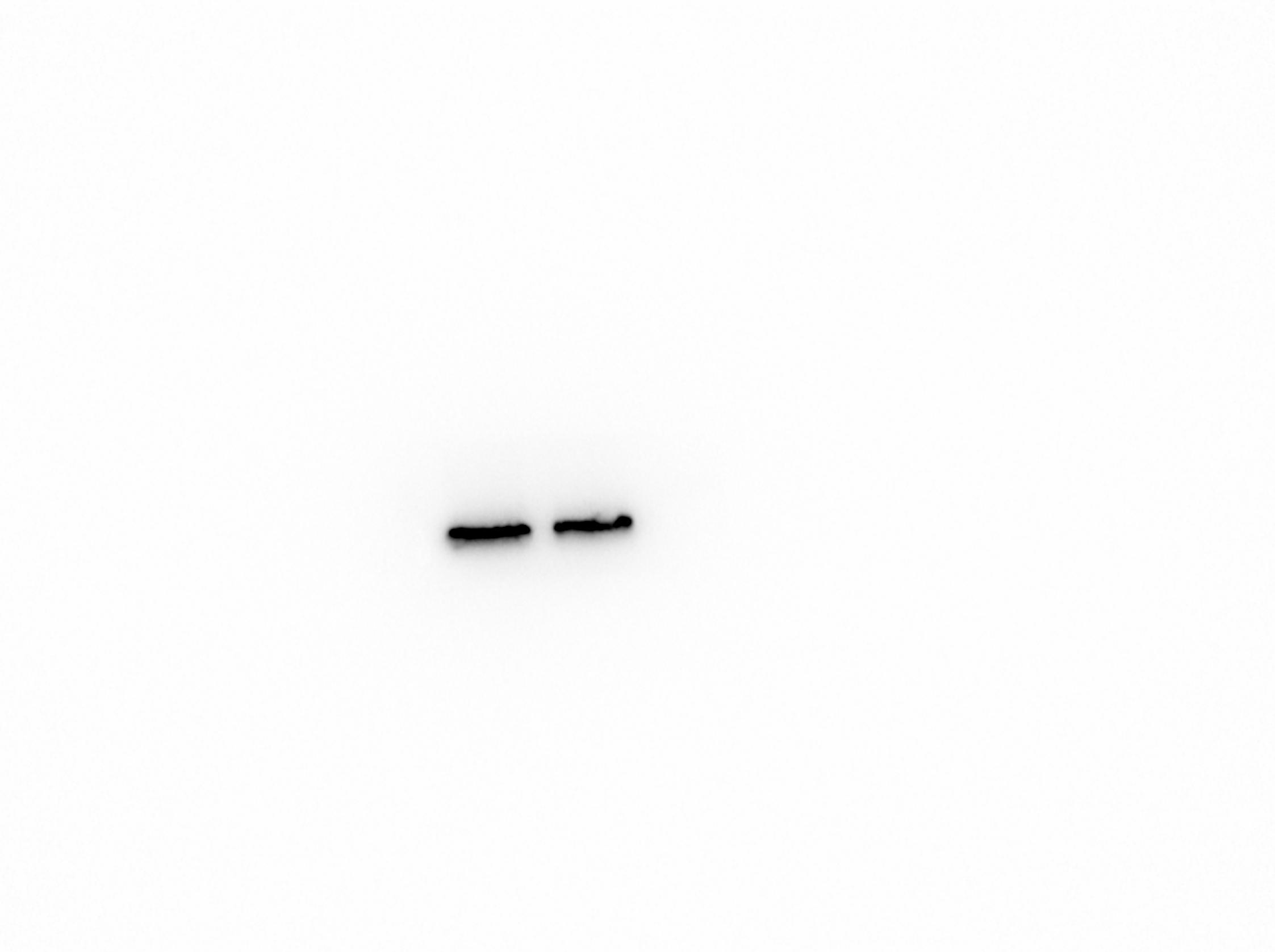

Supplement: Supplementary file 7 [file DataSheet_7.zip › GAPDH/Fig.5.2.tif]

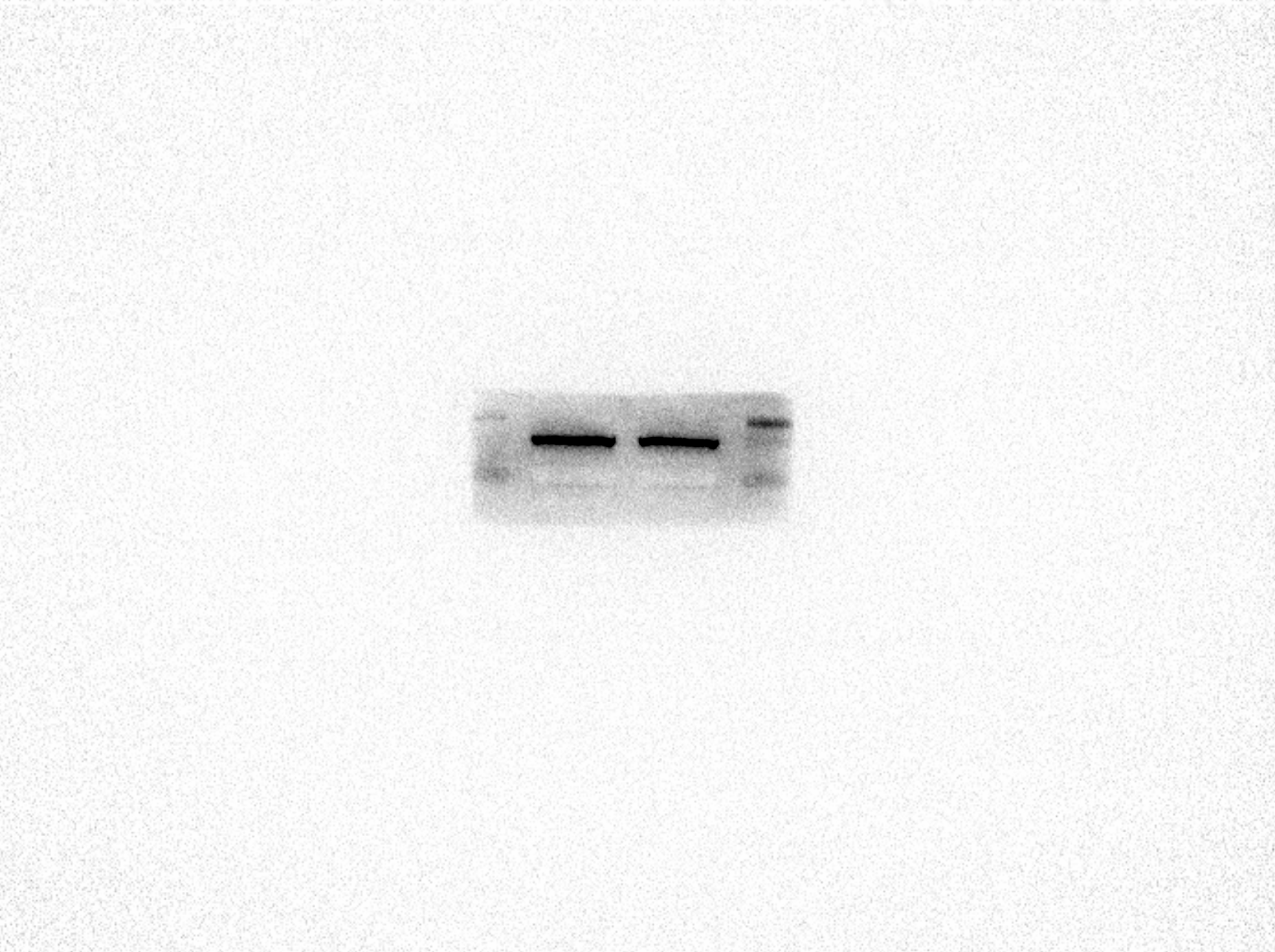

Supplement: Supplementary file 7 [file DataSheet_7.zip › GAPDH/Fig.5.3.tif]

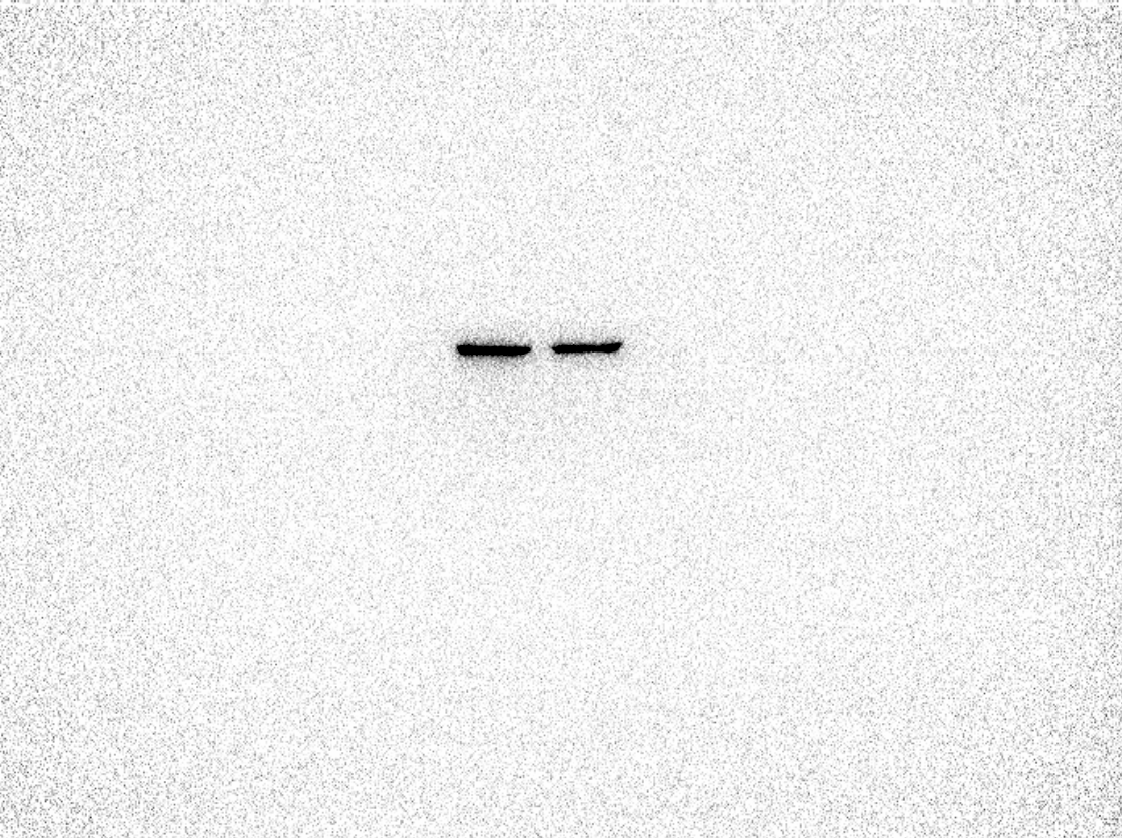

Supplement: Supplementary file 7 [file DataSheet_7.zip › GAPDH/Fig.7.1.tif]

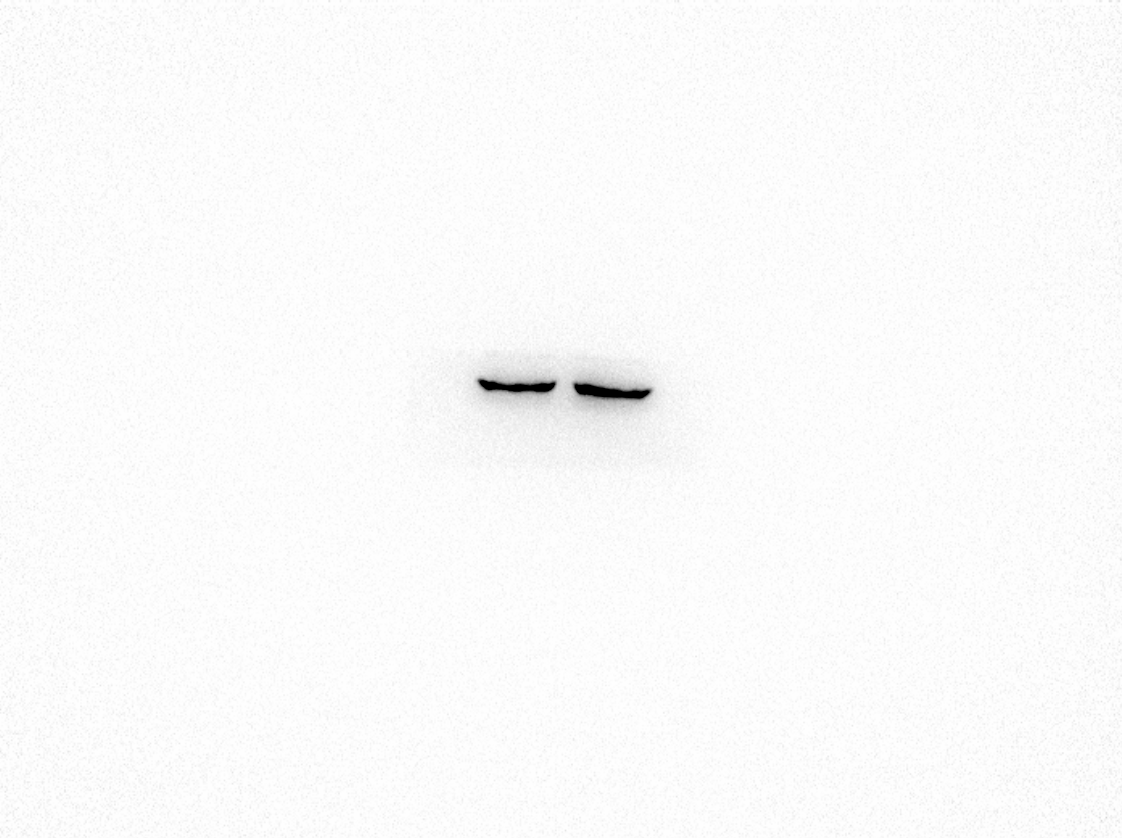

Supplement: Supplementary file 7 [file DataSheet_7.zip › GAPDH/Fig.7.2.tif]

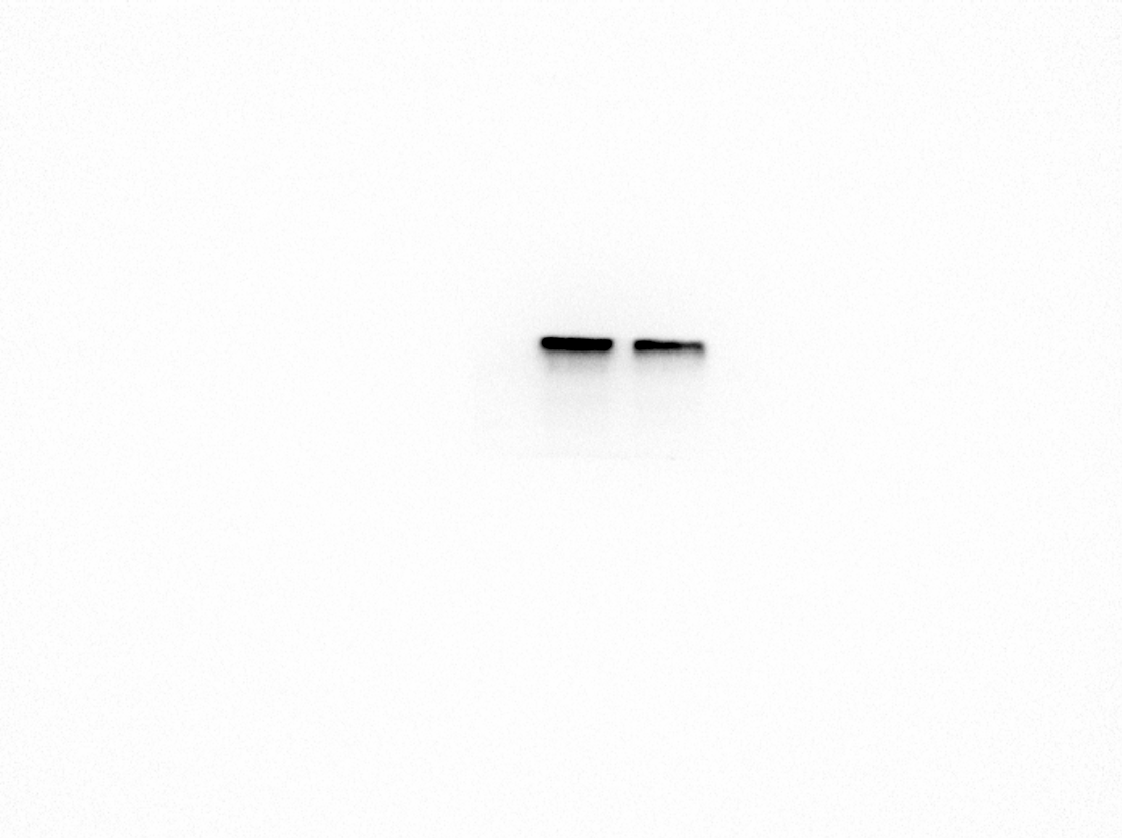

Supplement: Supplementary file 8 [file DataSheet_8.zip › MYD88/Fig.2.4.tif]

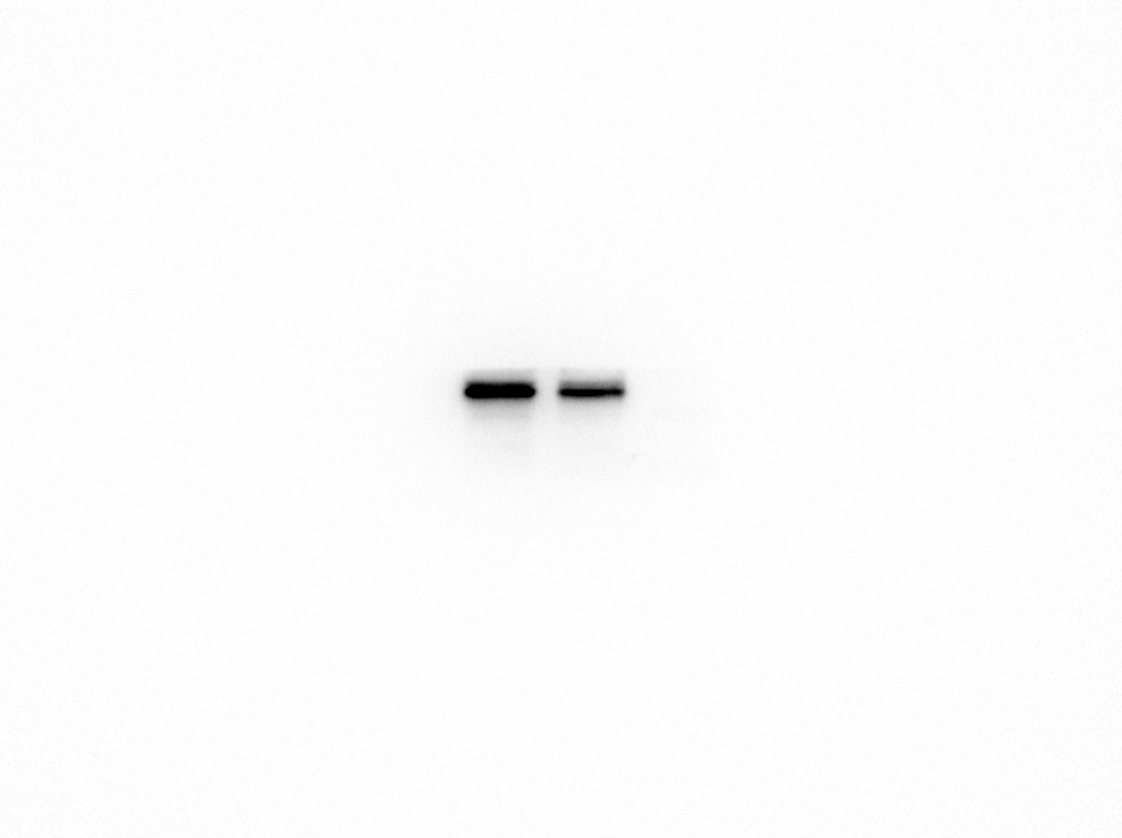

Supplement: Supplementary file 8 [file DataSheet_8.zip › MYD88/Fig.3.1.tif]

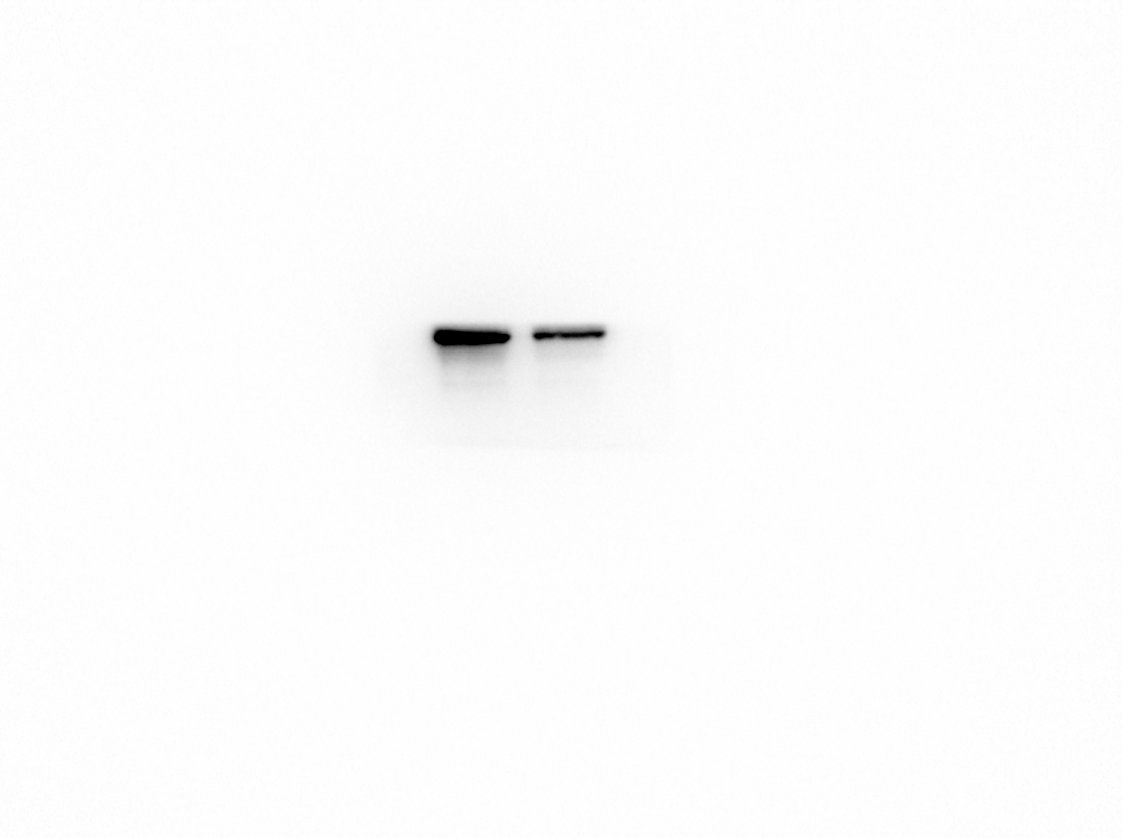

Supplement: Supplementary file 8 [file DataSheet_8.zip › MYD88/Fig.3.2.tif]

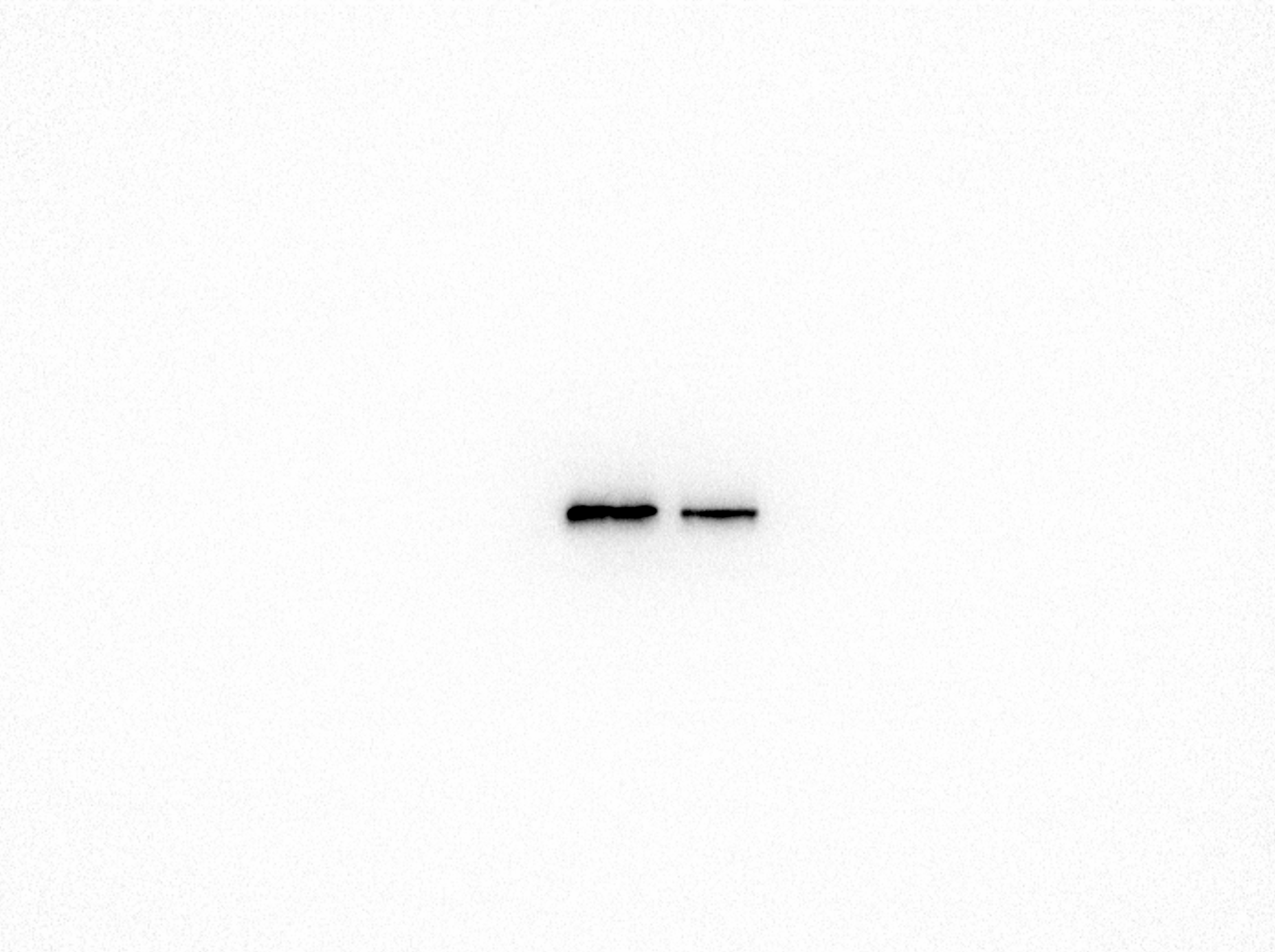

Supplement: Supplementary file 8 [file DataSheet_8.zip › MYD88/Fig.3.3.tif]

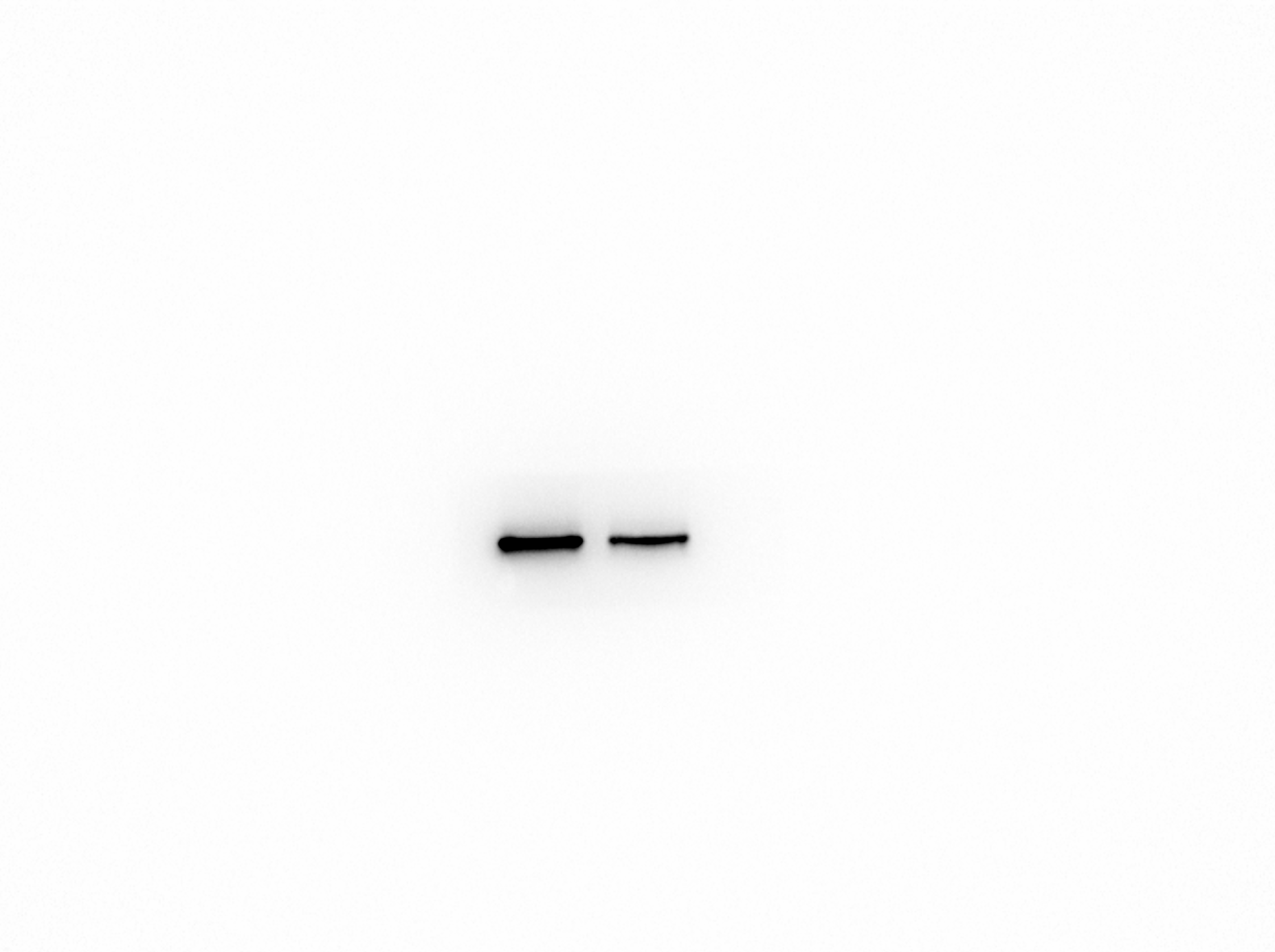

Supplement: Supplementary file 8 [file DataSheet_8.zip › MYD88/Fig.3.4.tif]

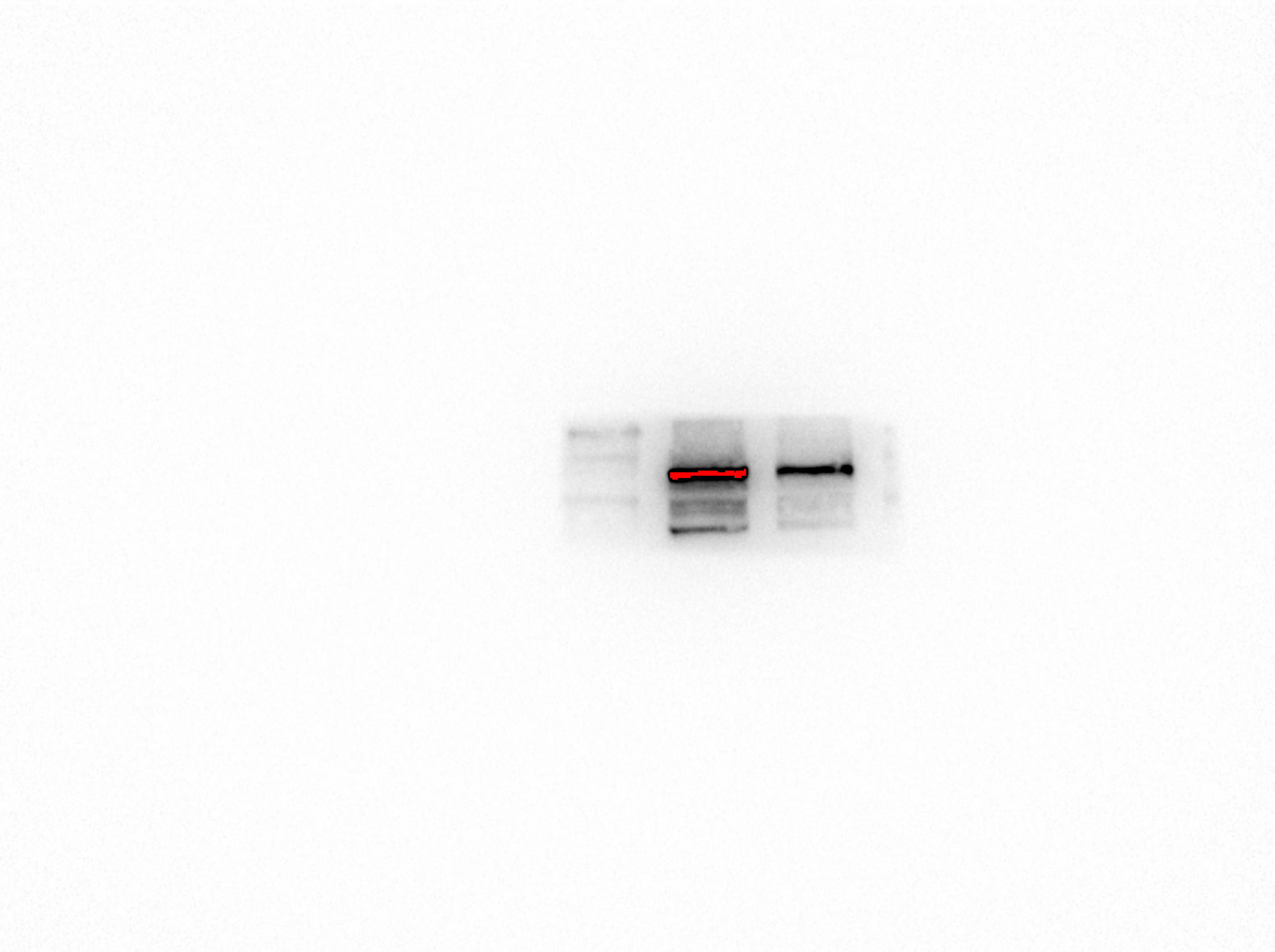

Supplement: Supplementary file 8 [file DataSheet_8.zip › MYD88/Fig.5.1.tif]

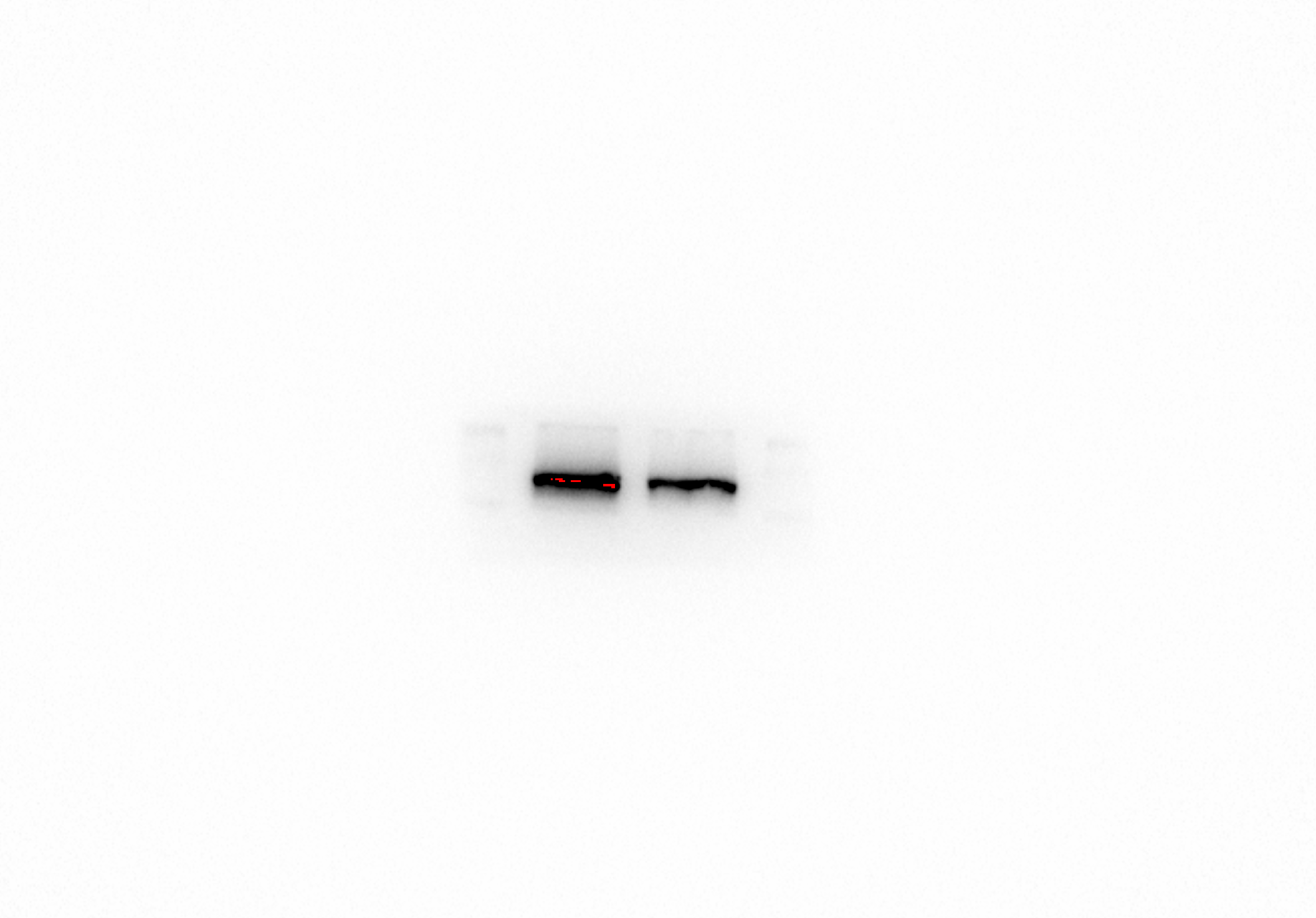

Supplement: Supplementary file 8 [file DataSheet_8.zip › MYD88/Fig.5.2.tif]

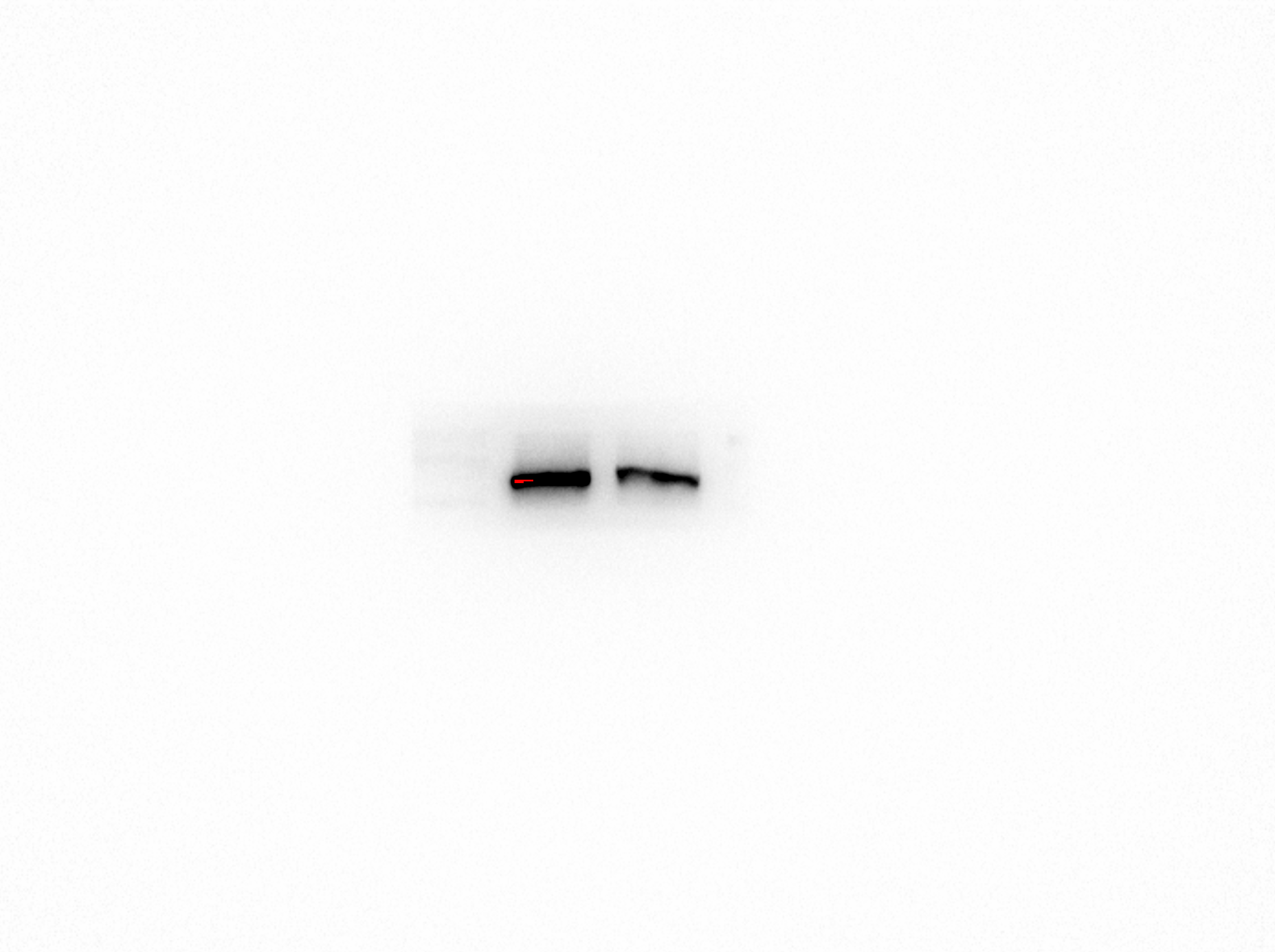

Supplement: Supplementary file 8 [file DataSheet_8.zip › MYD88/Fig.5.3.tif]

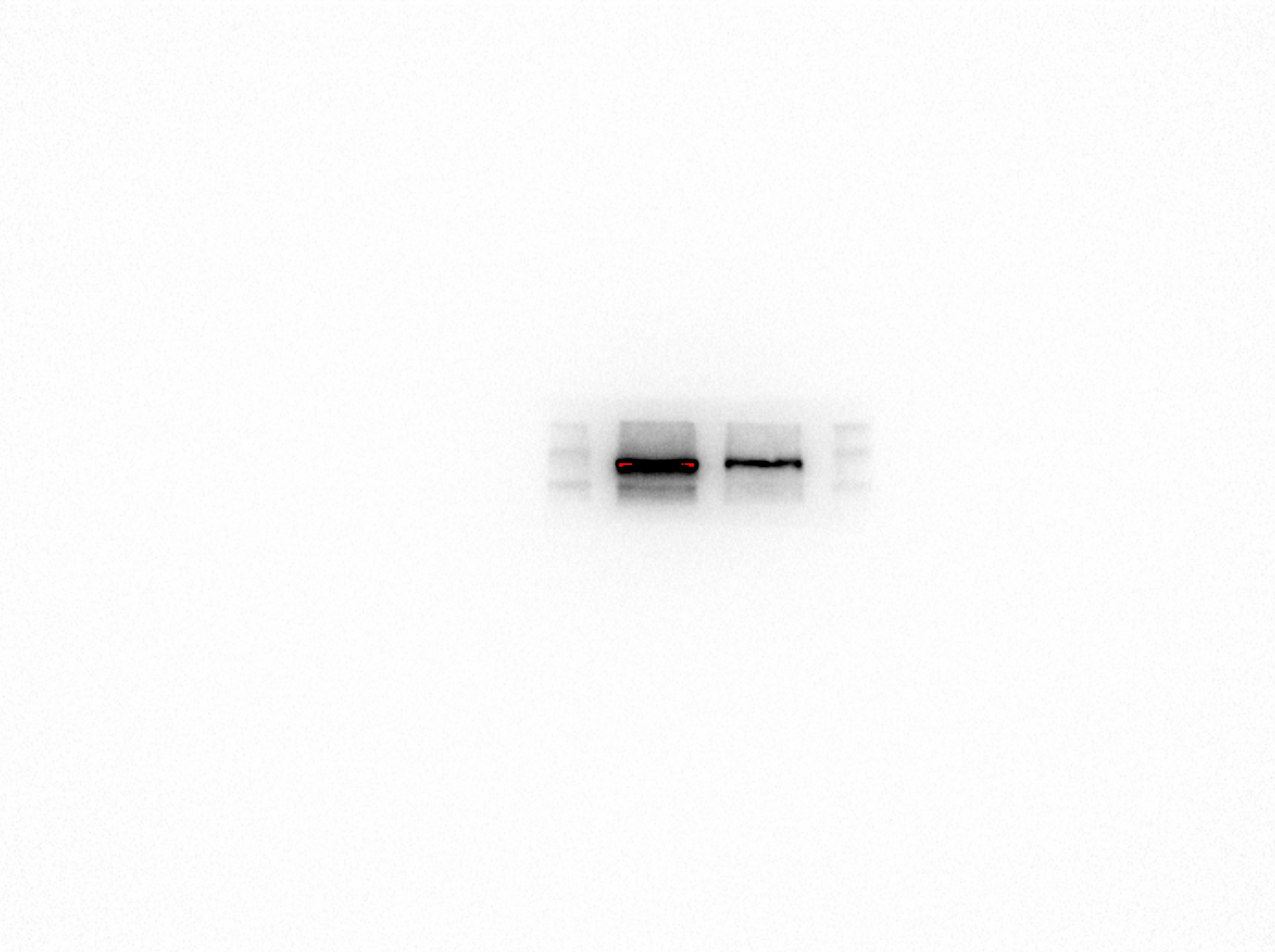

Supplement: Supplementary file 8 [file DataSheet_8.zip › MYD88/Fig.5.4.tif]

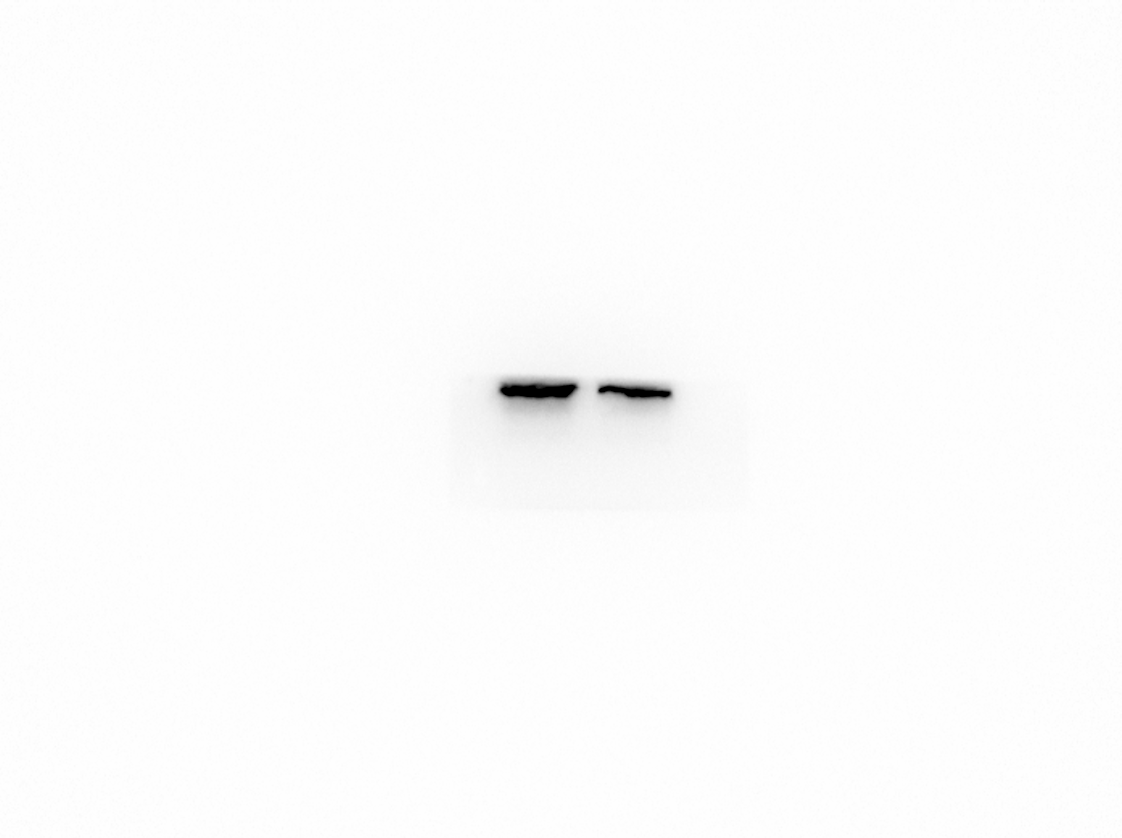

Supplement: Supplementary file 8 [file DataSheet_8.zip › MYD88/Fig.7.2.tif]

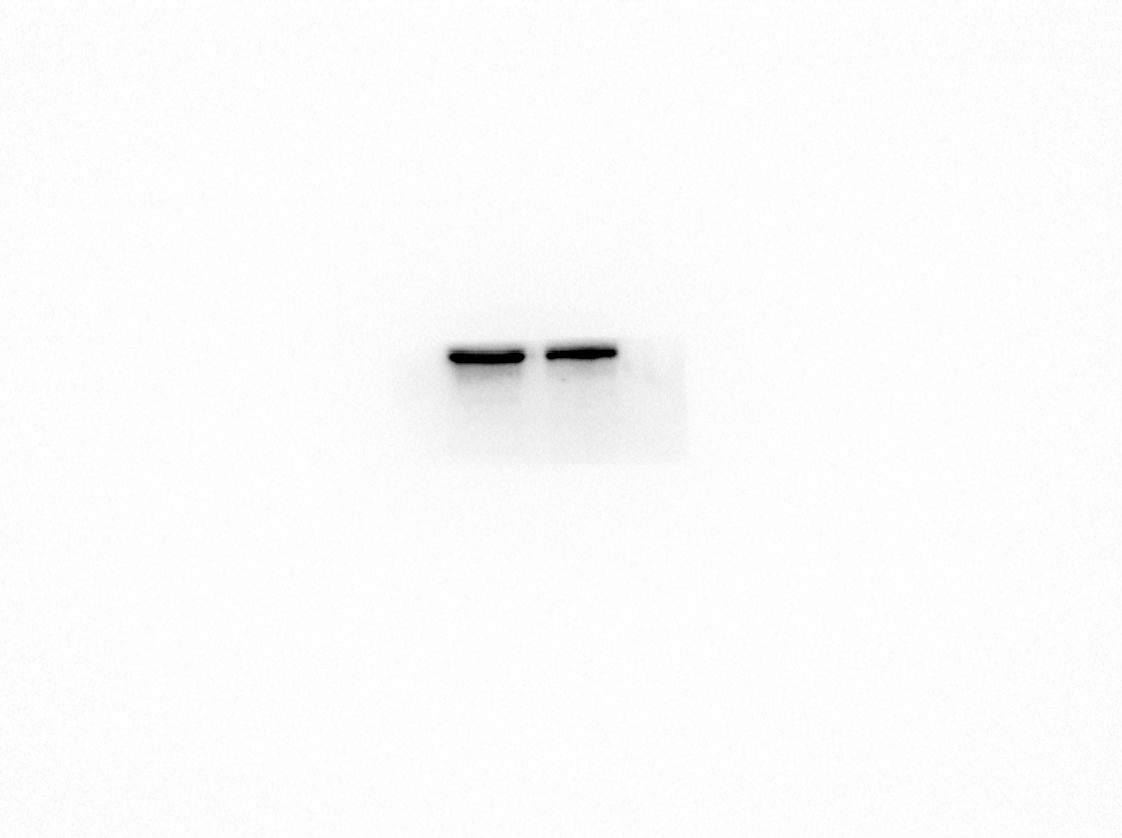

Supplement: Supplementary file 9 [file DataSheet_9.zip › NF-κB/Fig.3.1.tif]

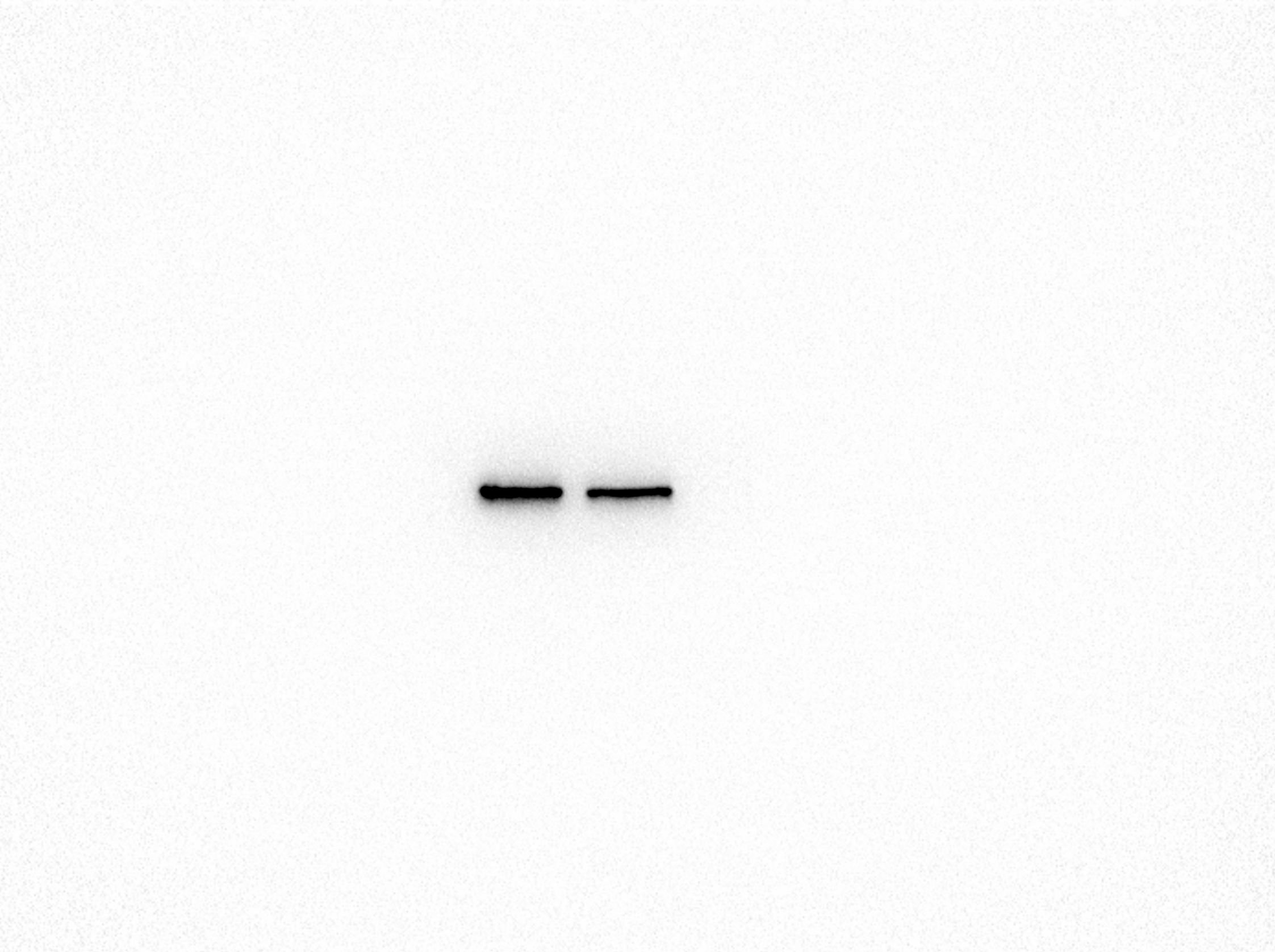

Supplement: Supplementary file 9 [file DataSheet_9.zip › NF-κB/Fig.3.2.tif]

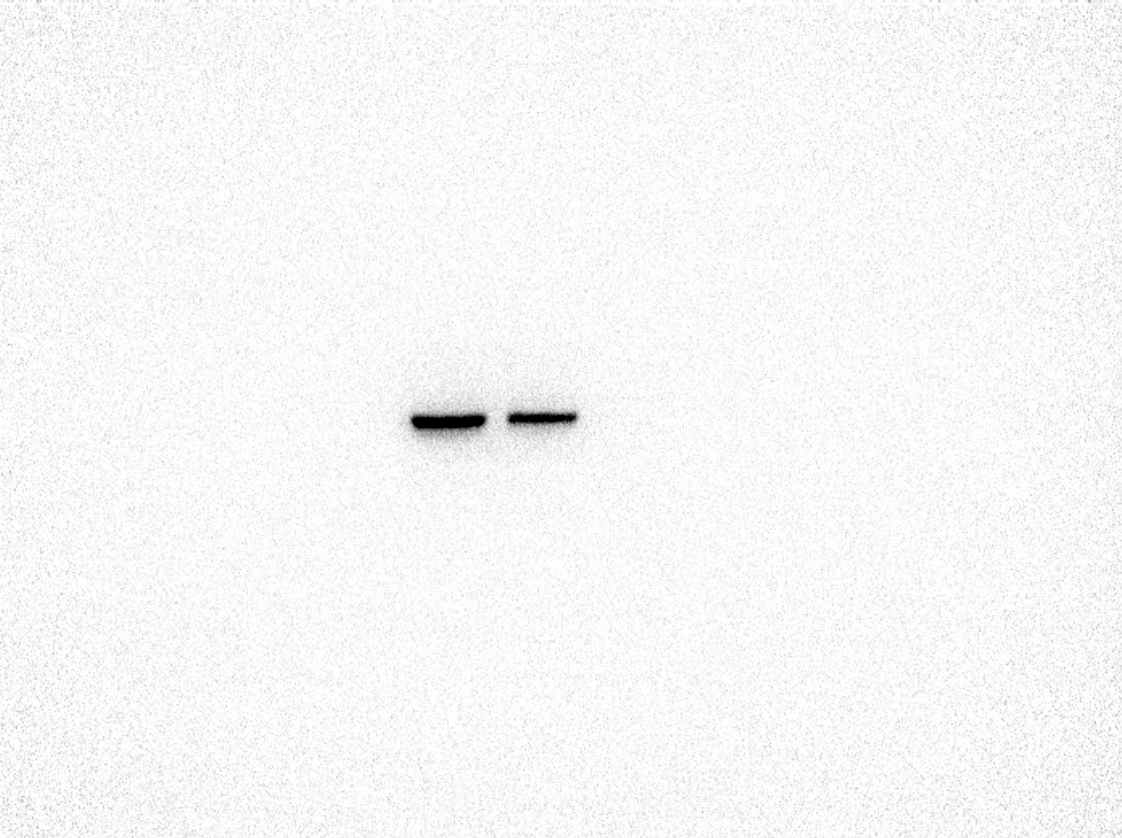

Supplement: Supplementary file 9 [file DataSheet_9.zip › NF-κB/Fig.3.3.tif]

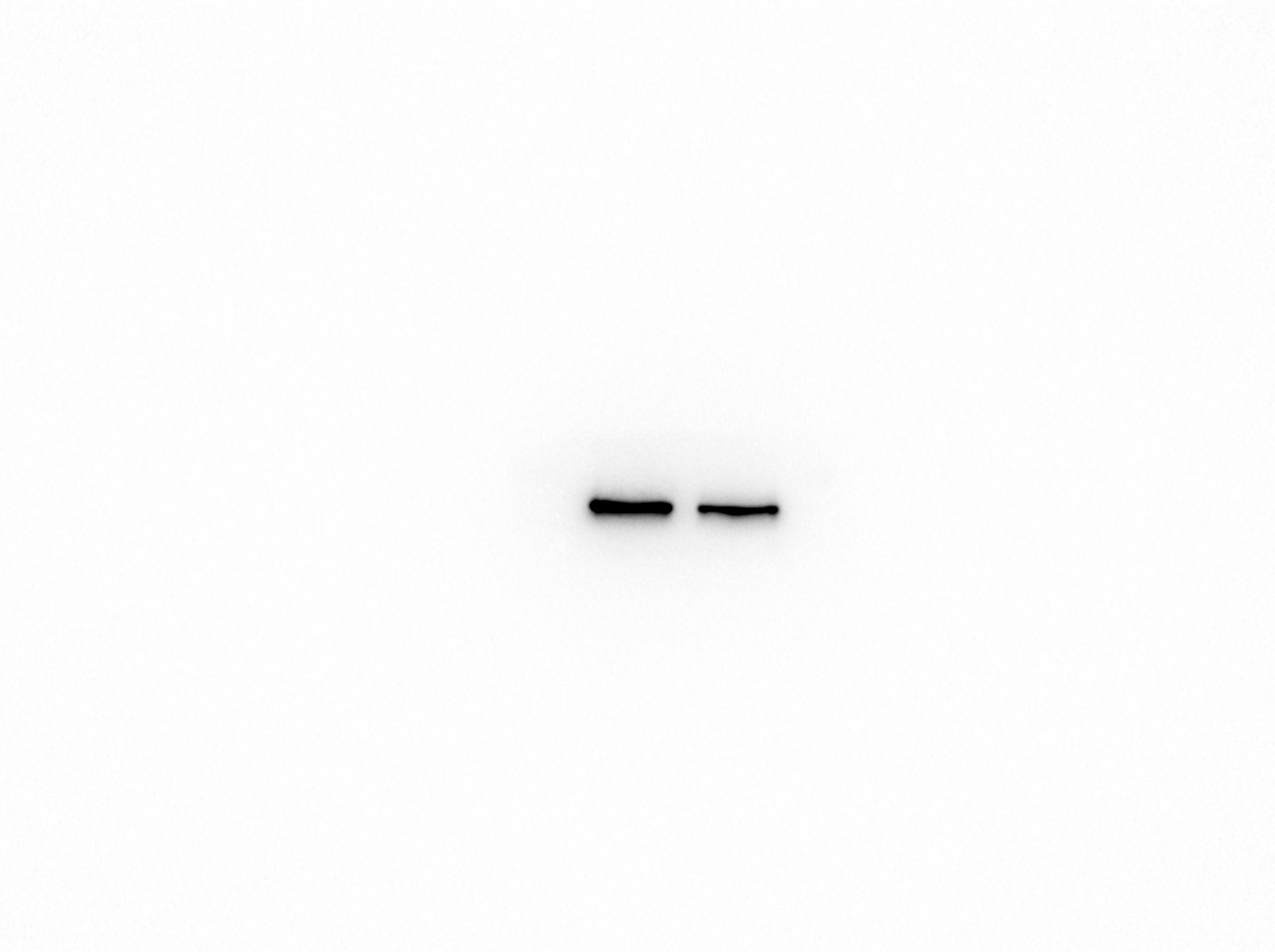

Supplement: Supplementary file 9 [file DataSheet_9.zip › NF-κB/Fig.3.4.tif]

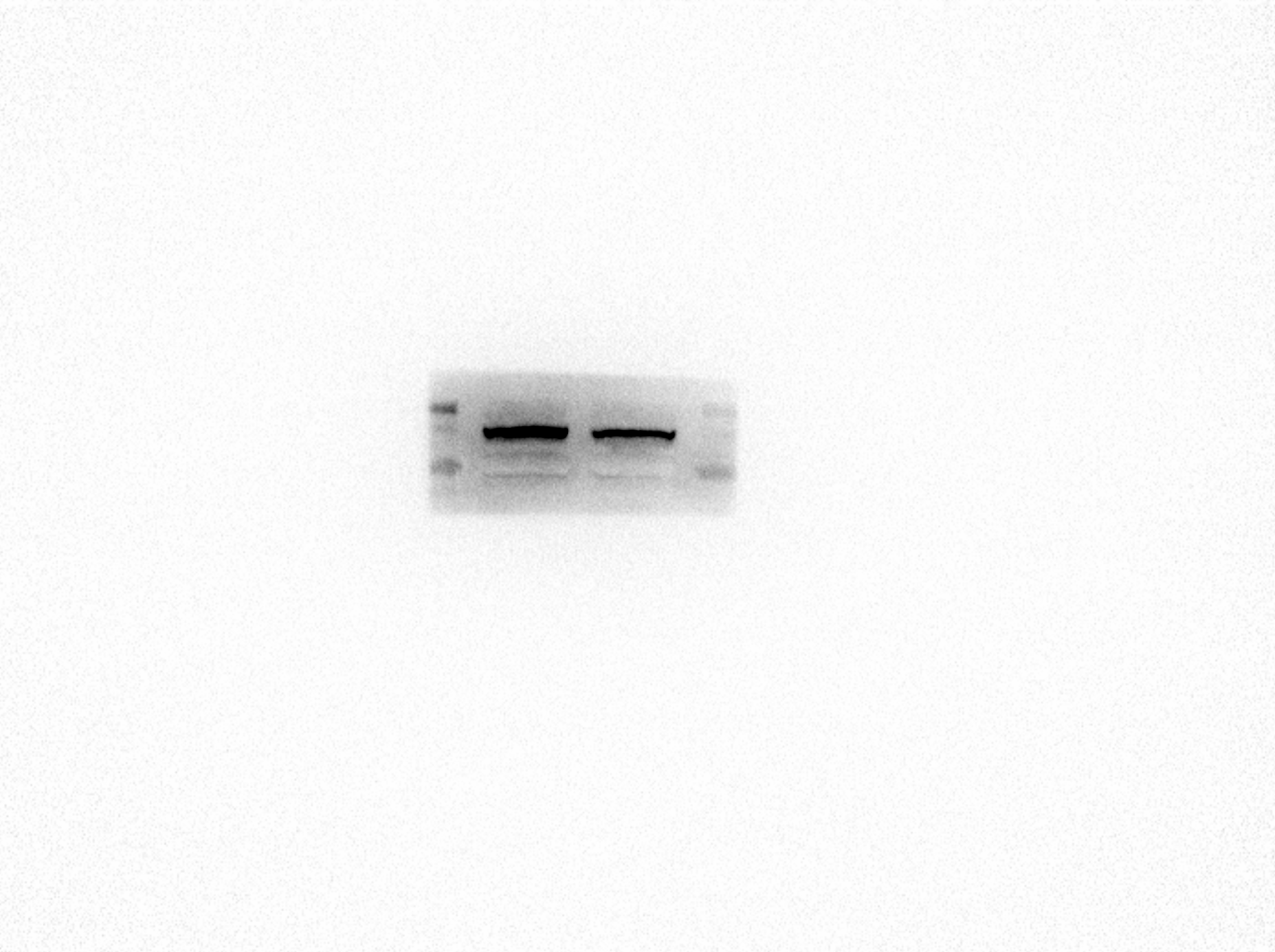

Supplement: Supplementary file 9 [file DataSheet_9.zip › NF-κB/Fig.5.1.tif]

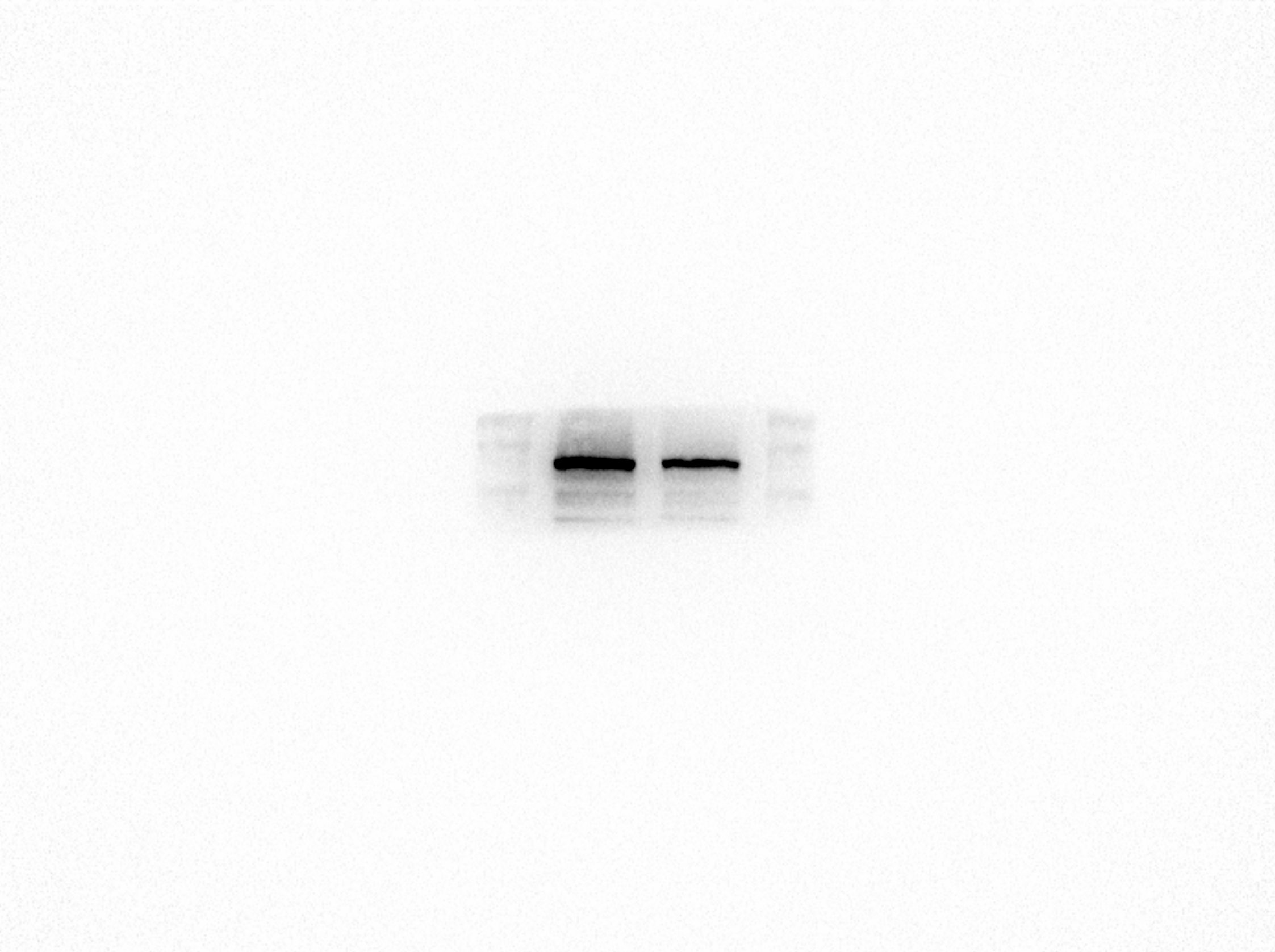

Supplement: Supplementary file 9 [file DataSheet_9.zip › NF-κB/Fig.5.2.tif]

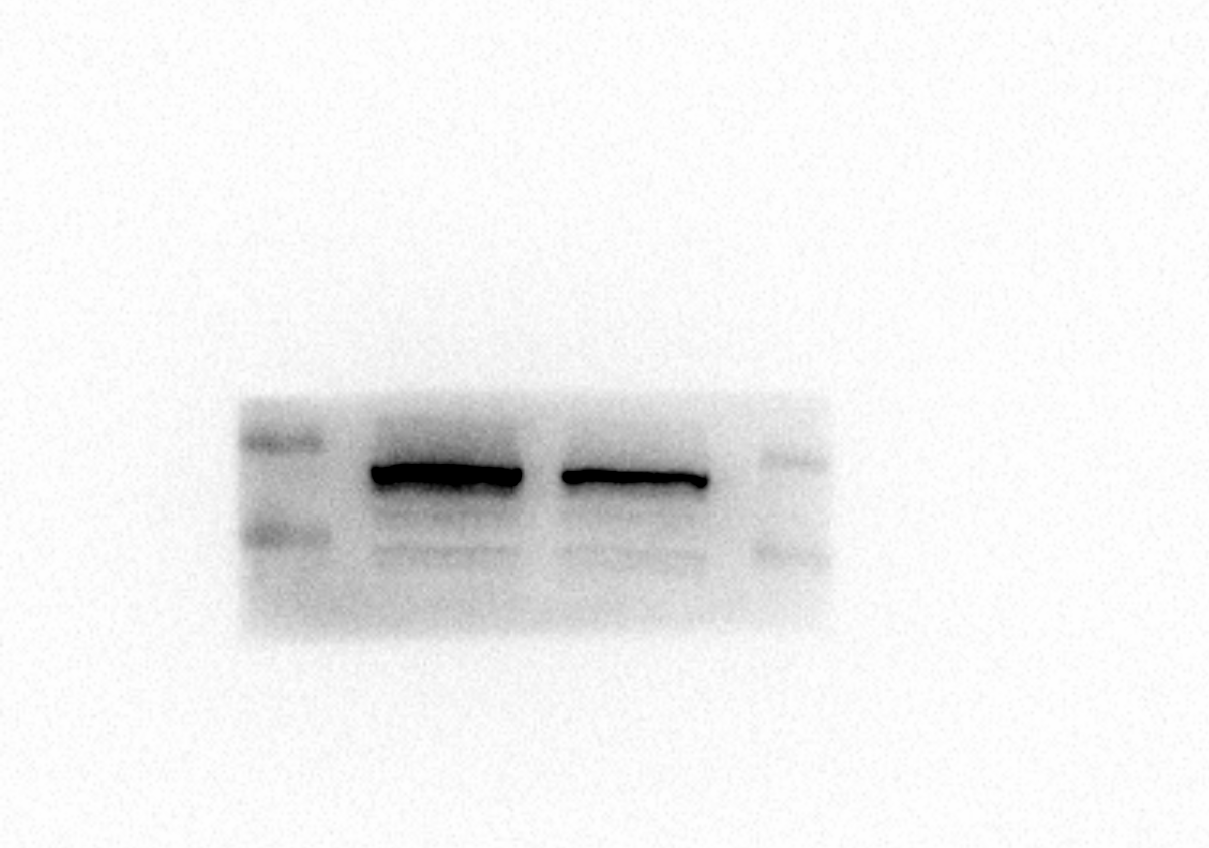

Supplement: Supplementary file 9 [file DataSheet_9.zip › NF-κB/Fig.5.3.tif]

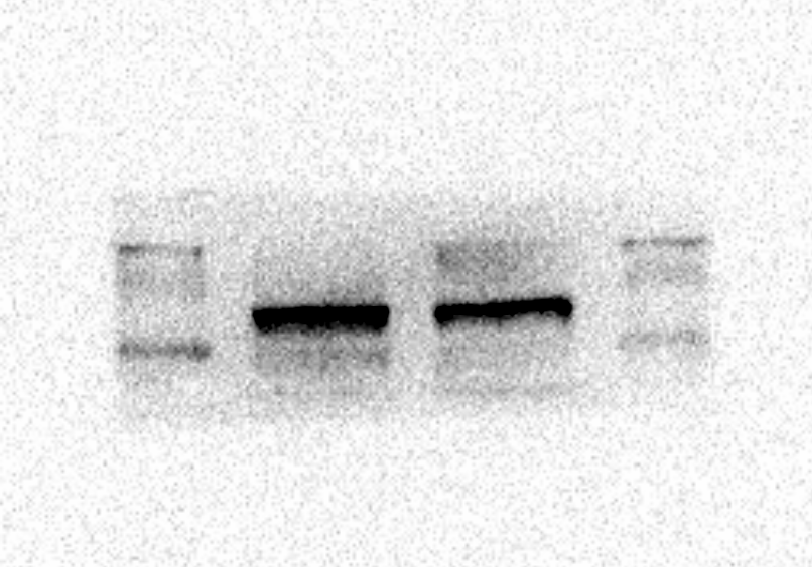

Supplement: Supplementary file 9 [file DataSheet_9.zip › NF-κB/Fig.5.4.tif]

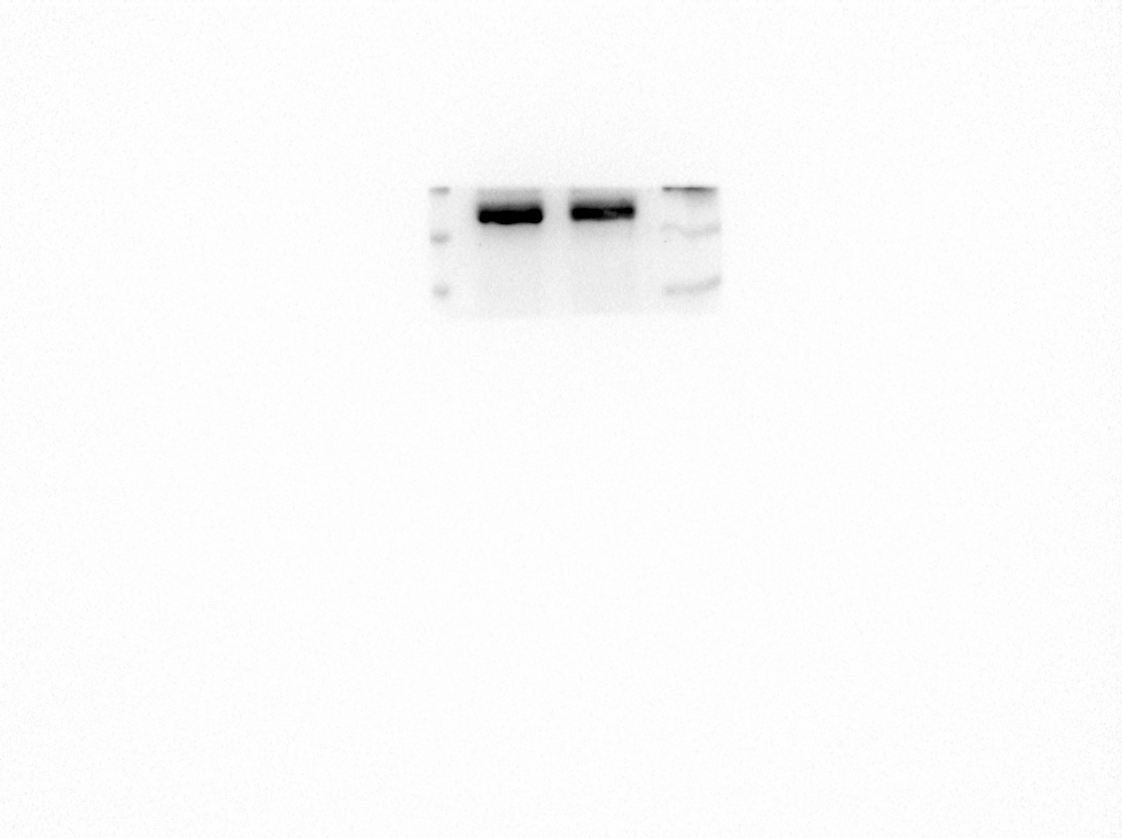

Supplement: Supplementary file 9 [file DataSheet_9.zip › NF-κB/Fig.7.1.tif]

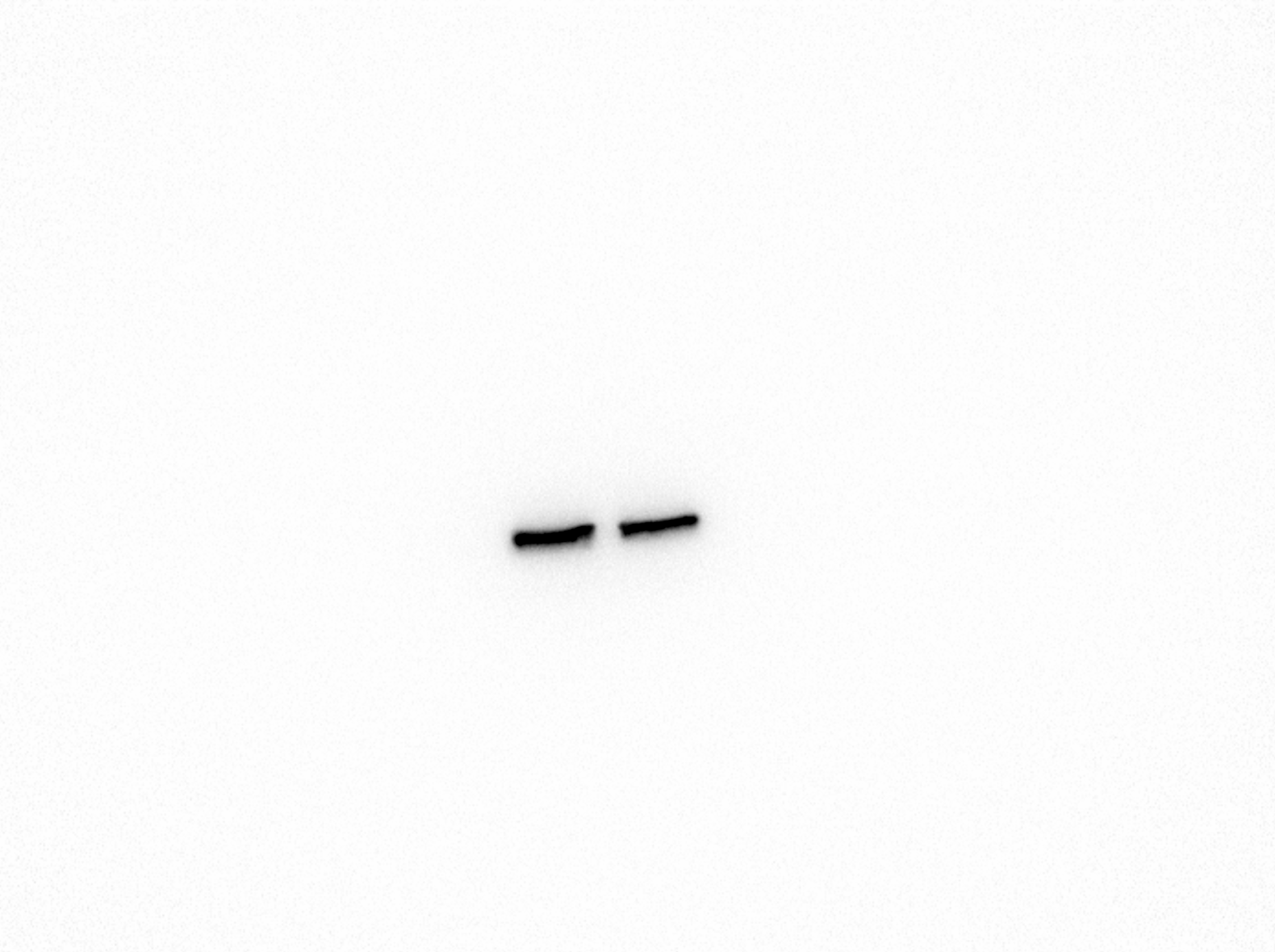

Supplement: Supplementary file 9 [file DataSheet_9.zip › NF-κB/Fig.7.2.tif]

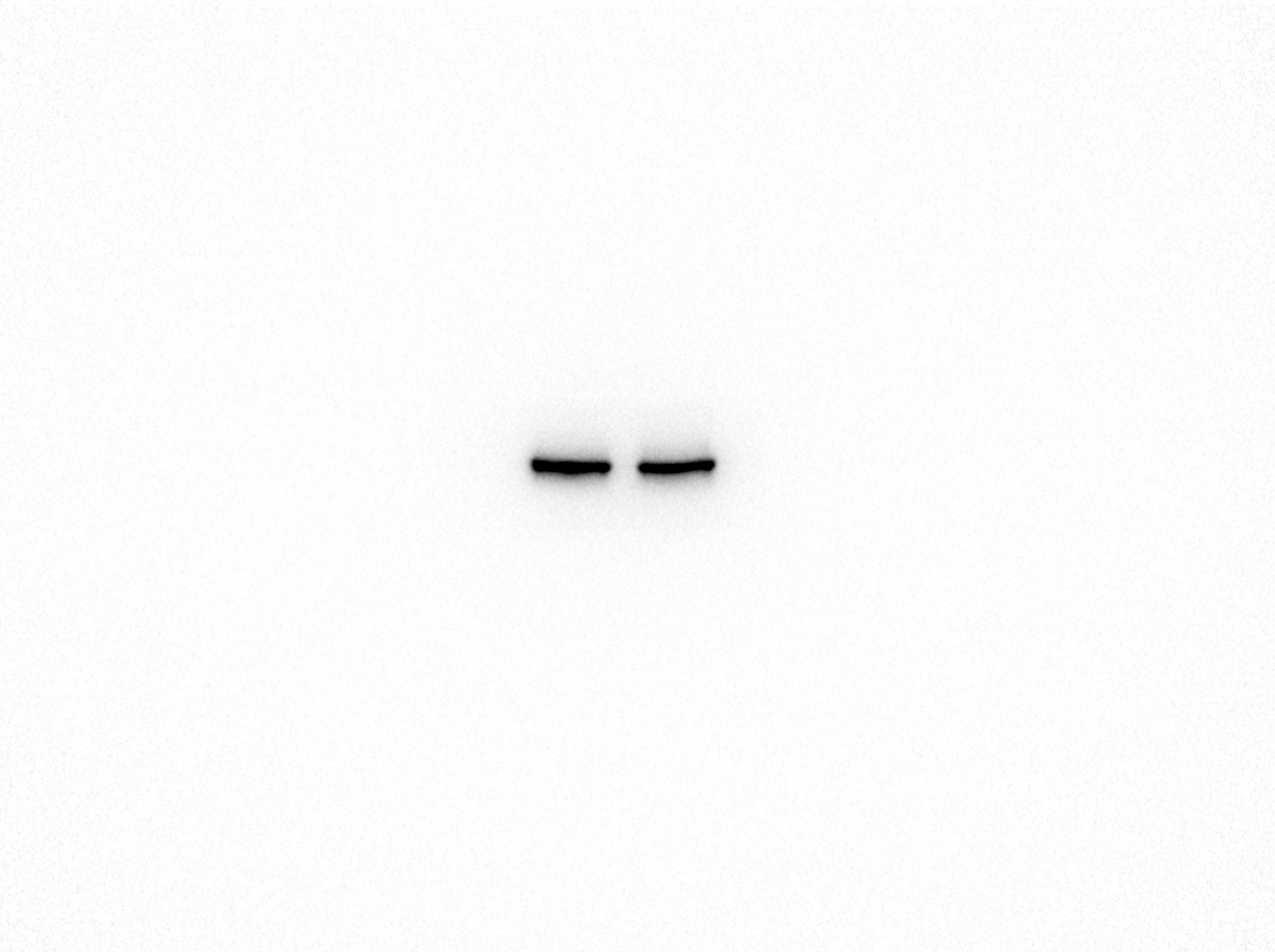

Supplement: Supplementary file 9 [file DataSheet_9.zip › NF-κB/Fig.7.3.tif]

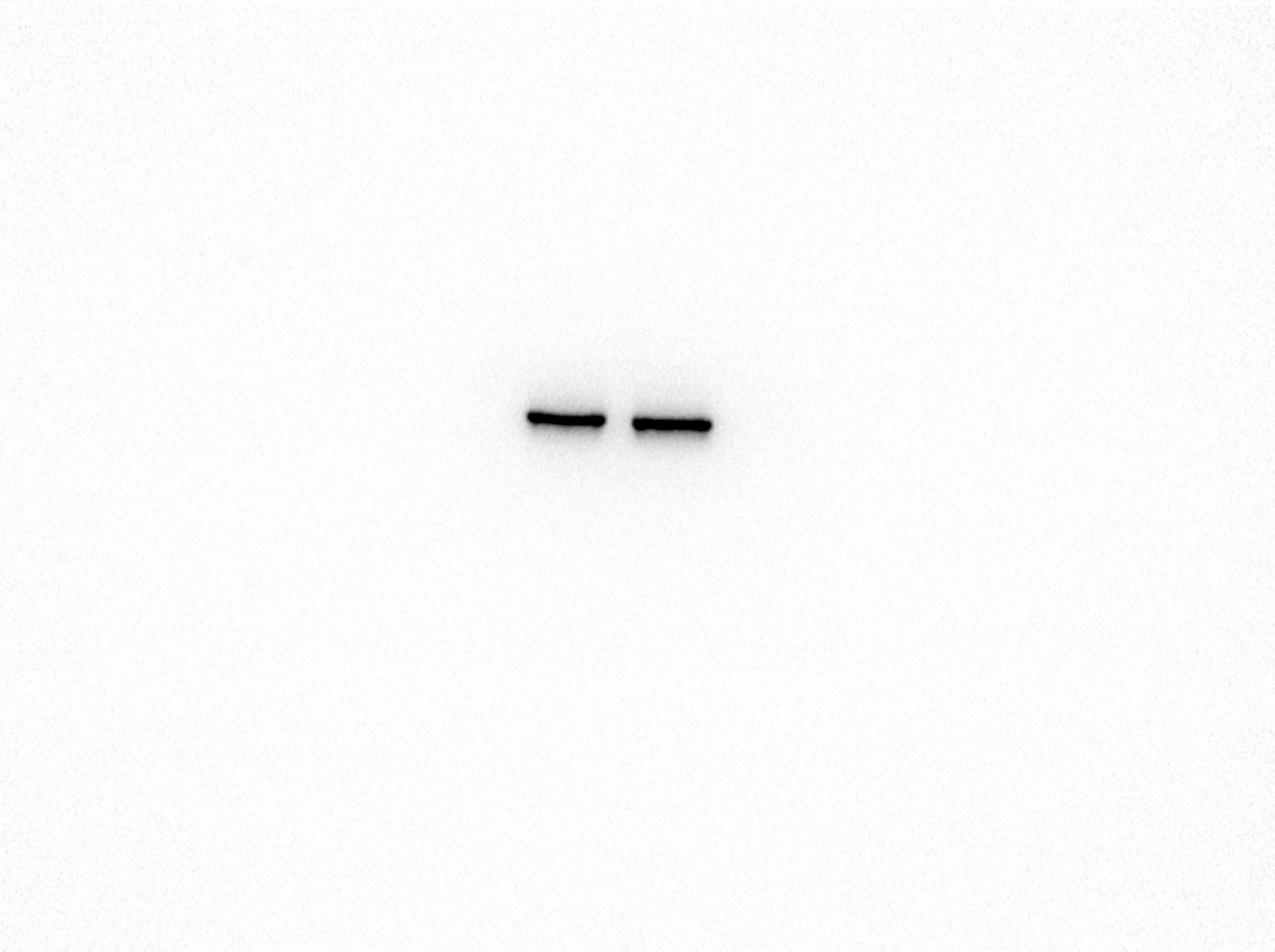

Supplement: Supplementary file 9 [file DataSheet_9.zip › NF-κB/Fig.7.4.tif]

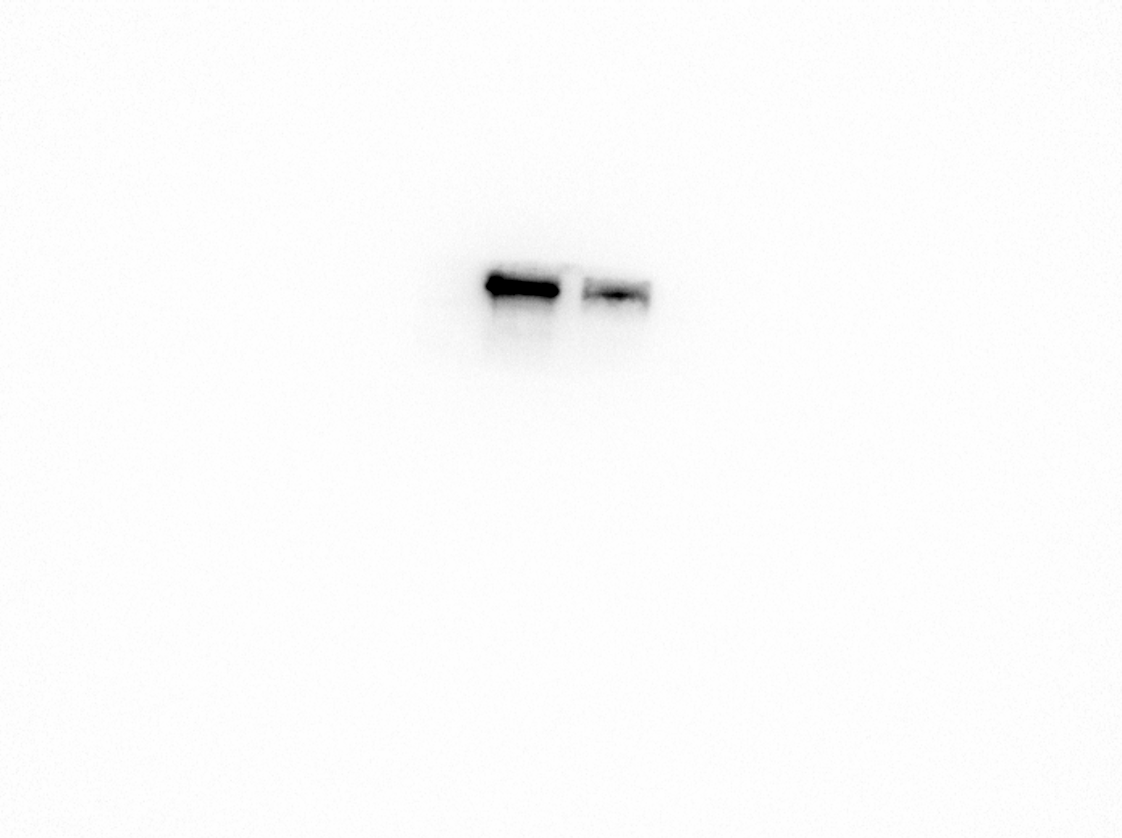

Supplement: Supplementary file 10 [file DataSheet_10.zip › TLR-4/Fig.3.1.tif]

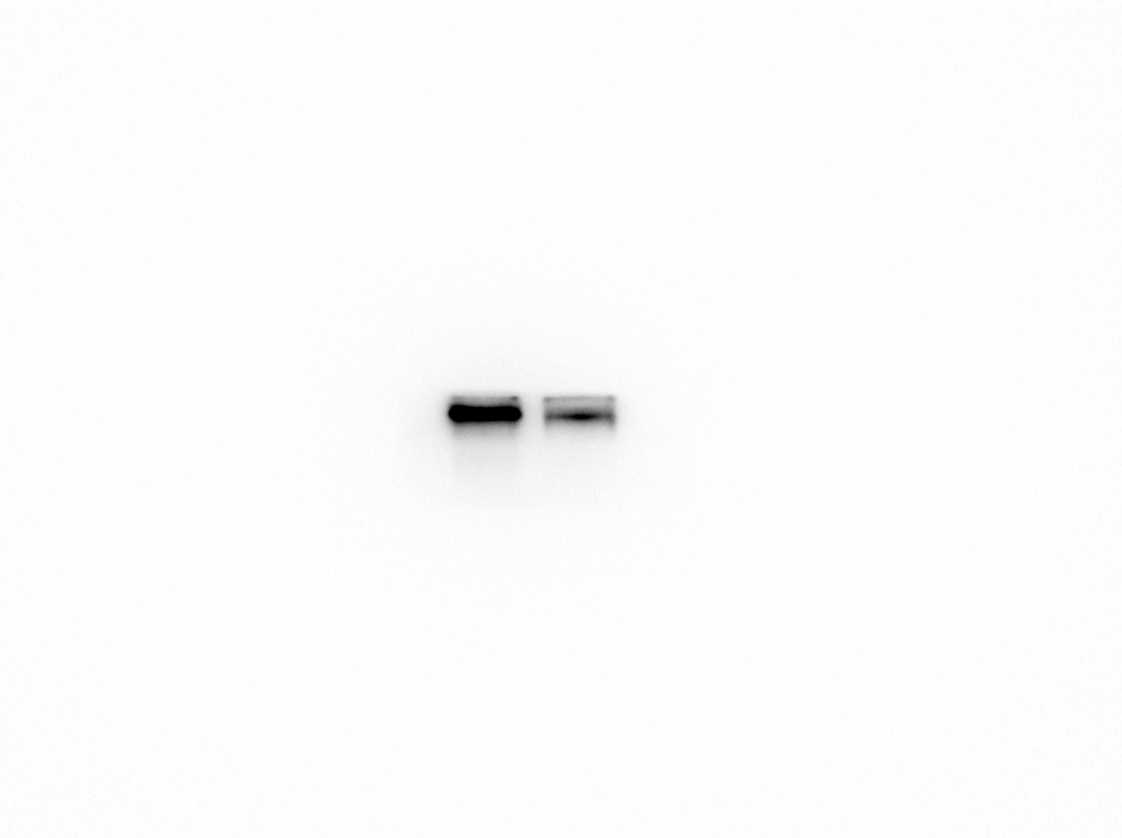

Supplement: Supplementary file 10 [file DataSheet_10.zip › TLR-4/Fig.3.2.tif]

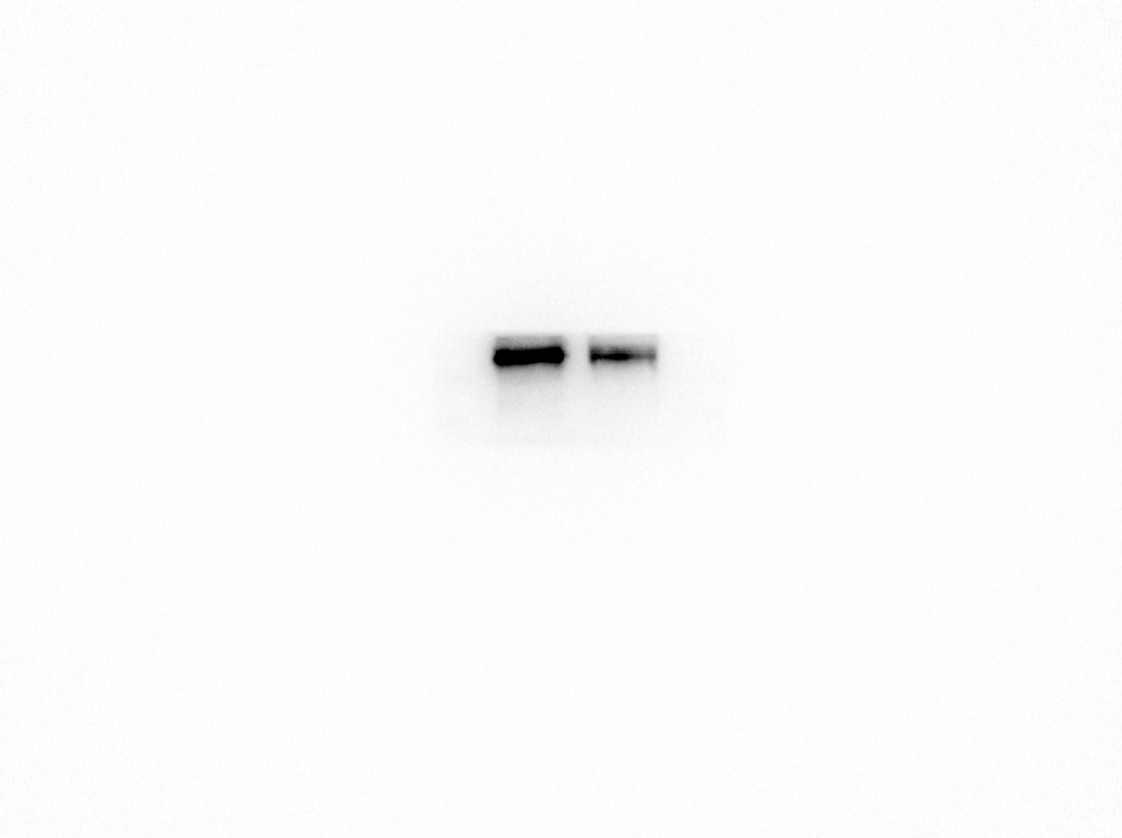

Supplement: Supplementary file 10 [file DataSheet_10.zip › TLR-4/Fig.3.3.tif]

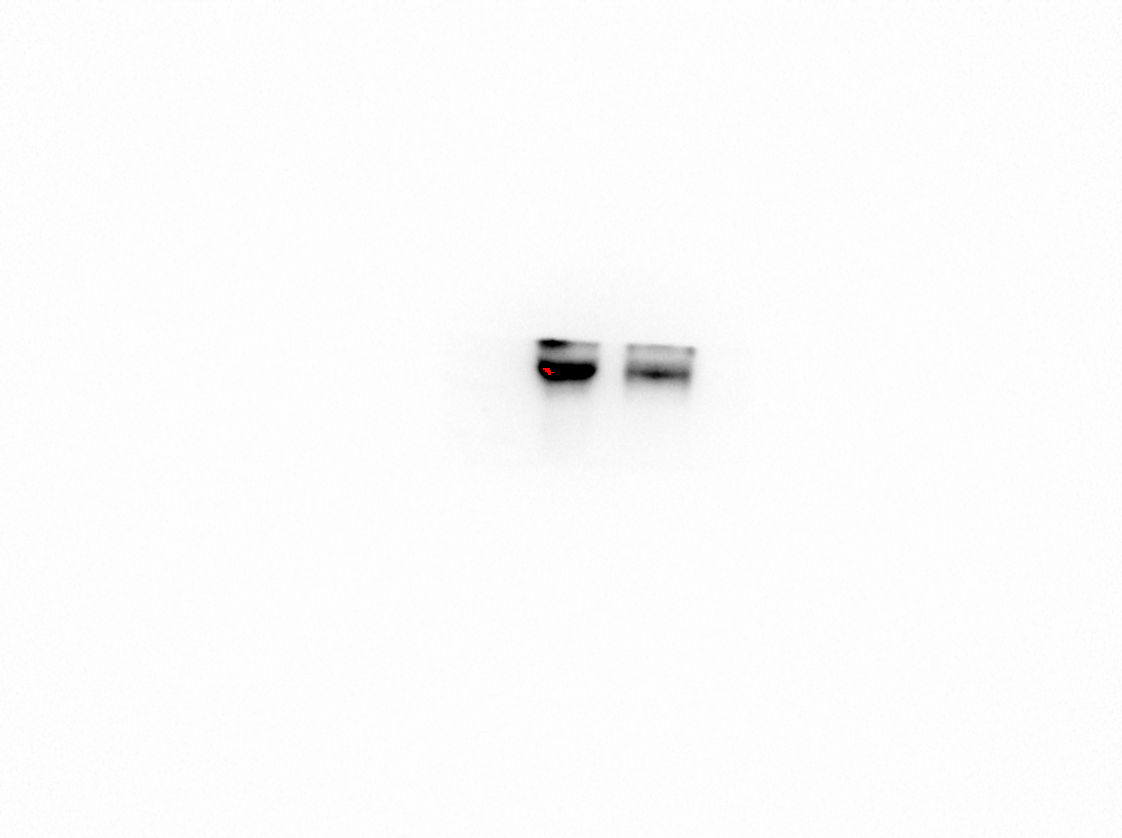

Supplement: Supplementary file 10 [file DataSheet_10.zip › TLR-4/Fig.3.4.tif]

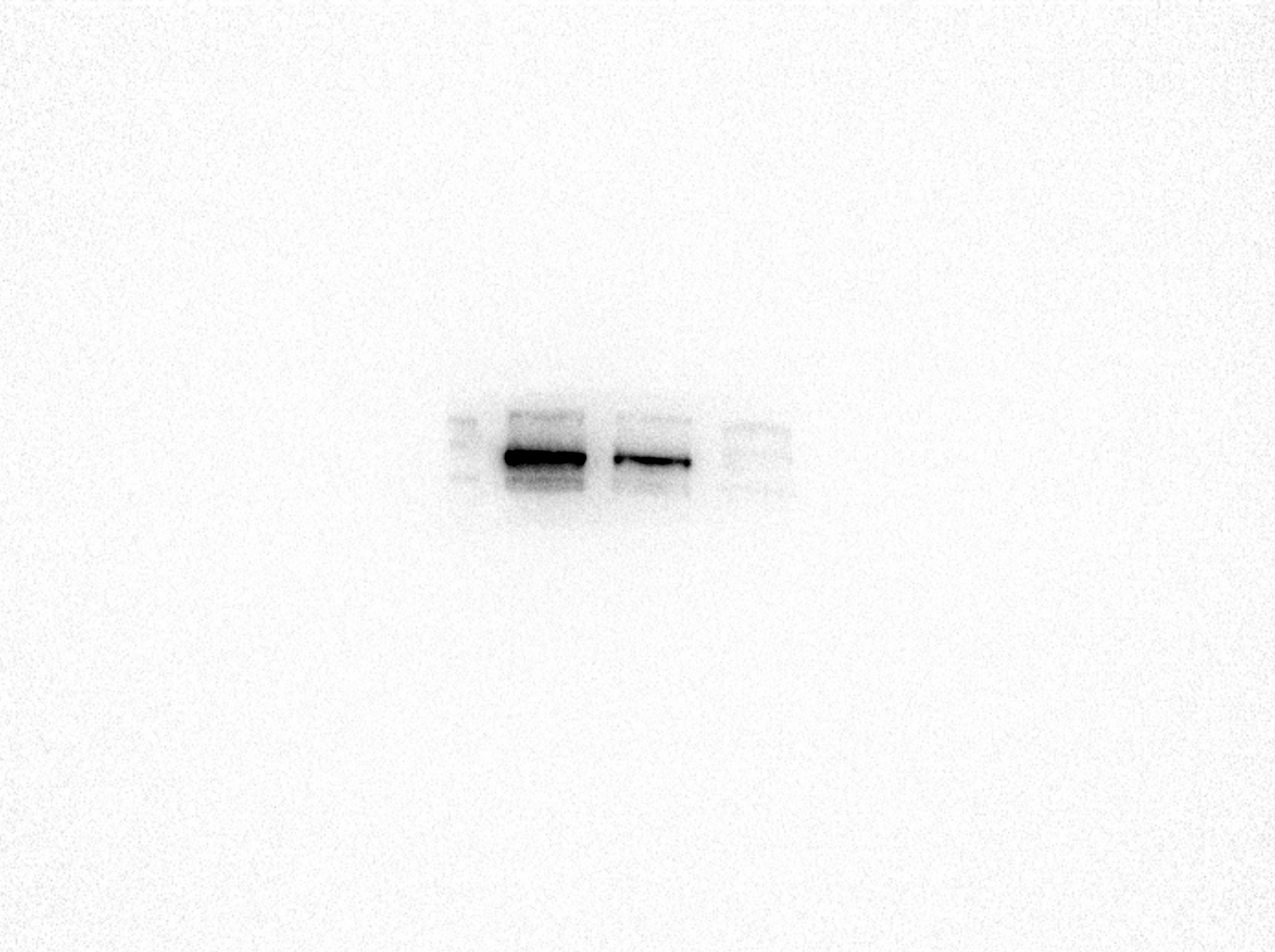

Supplement: Supplementary file 10 [file DataSheet_10.zip › TLR-4/Fig.4.2.tif]

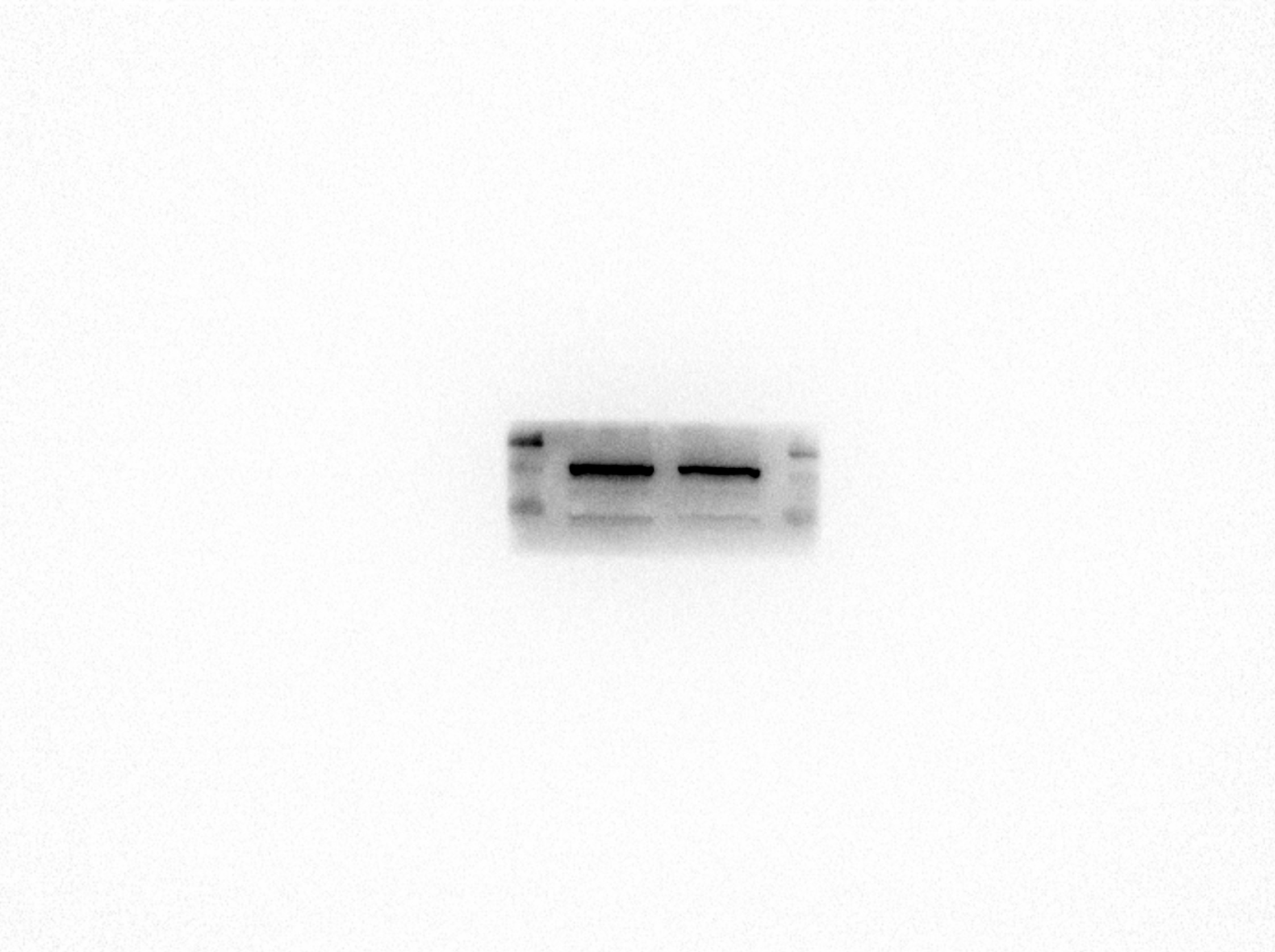

Supplement: Supplementary file 10 [file DataSheet_10.zip › TLR-4/Fig.4.3.tif]

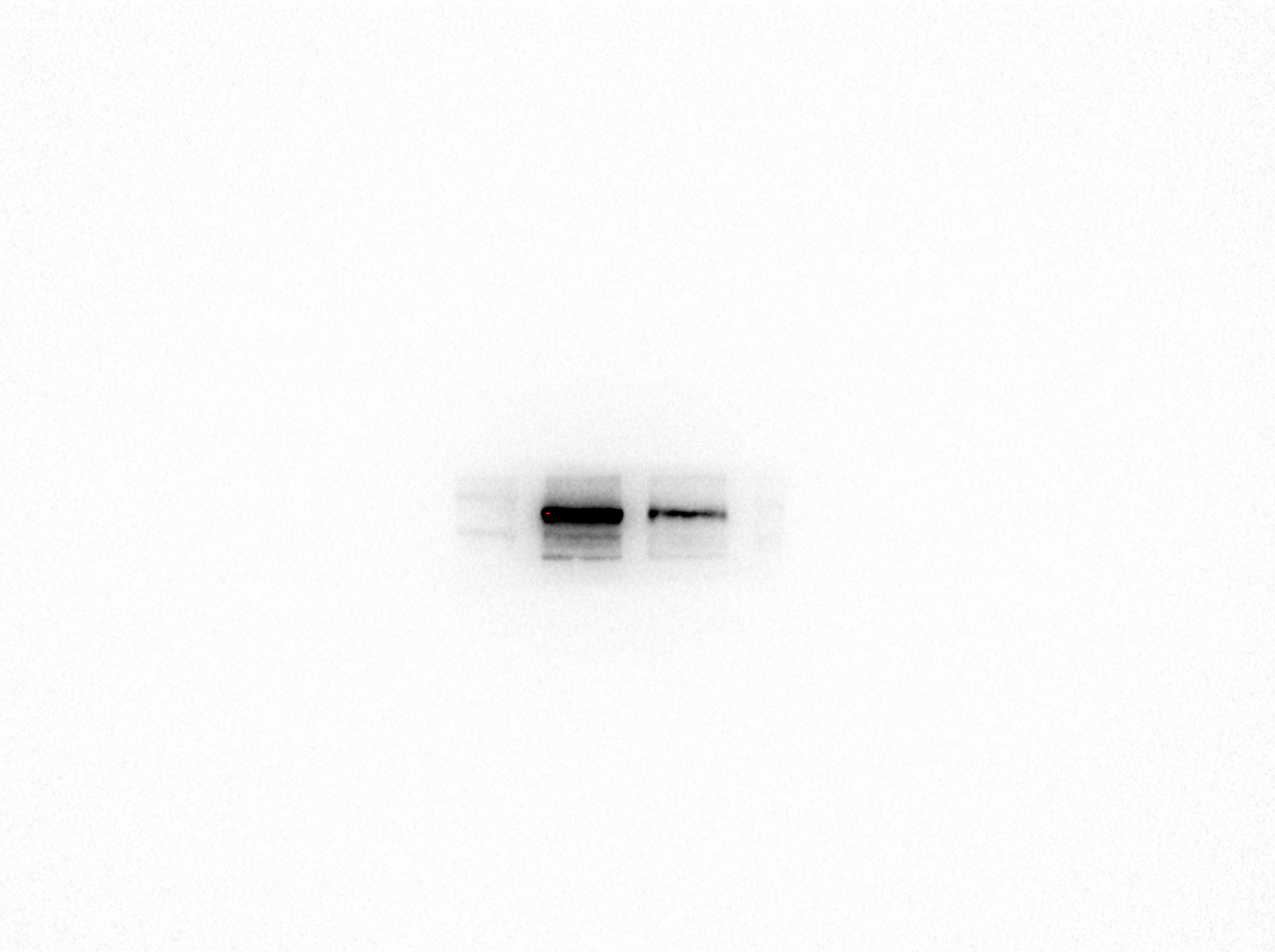

Supplement: Supplementary file 10 [file DataSheet_10.zip › TLR-4/Fig.5.1.Tif]

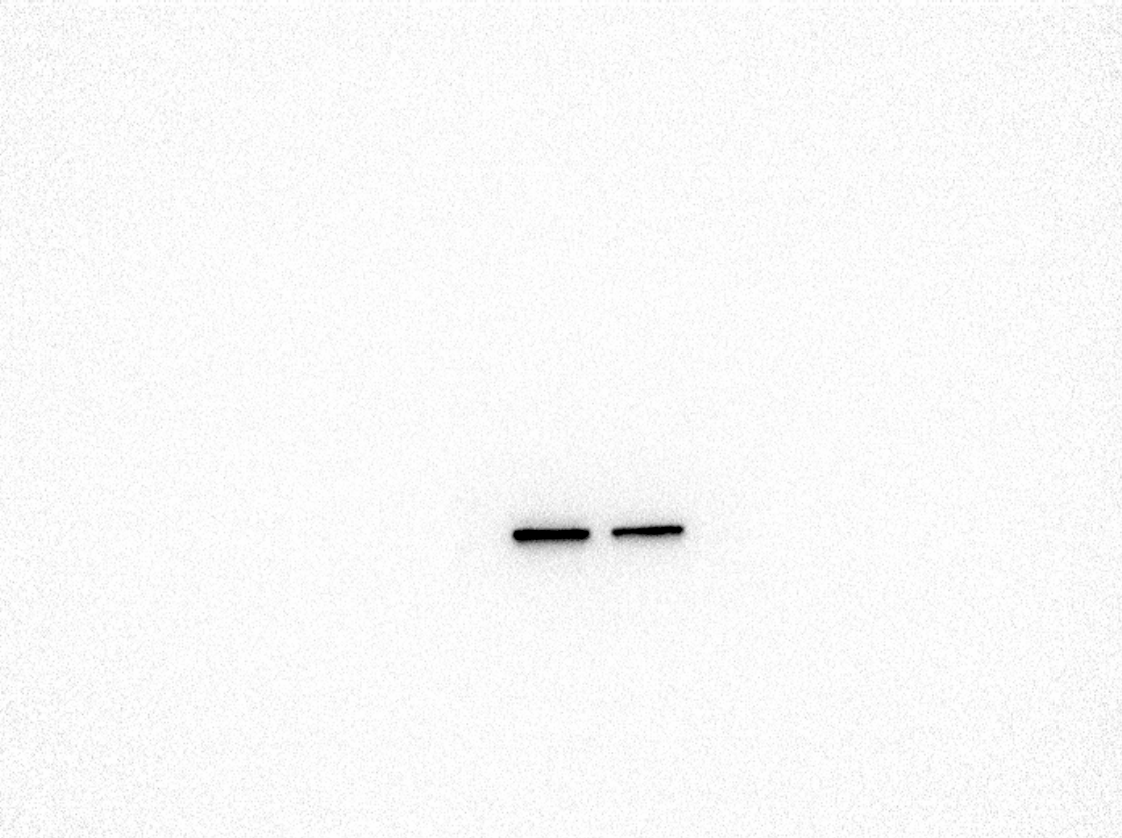

Supplement: Supplementary file 10 [file DataSheet_10.zip › TLR-4/Fig.5.2.tif]

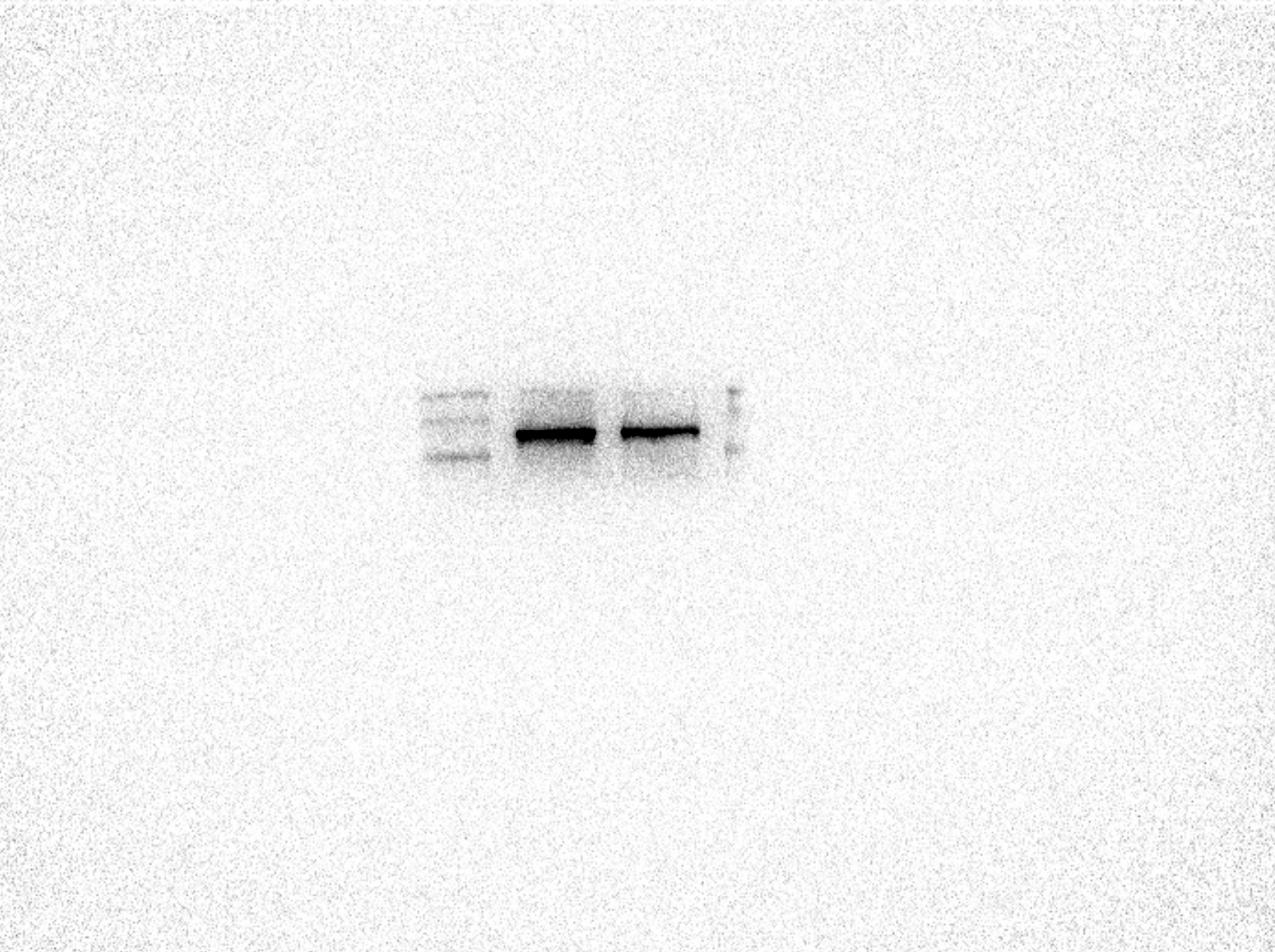

Supplement: Supplementary file 10 [file DataSheet_10.zip › TLR-4/Fig.5.3.tif]

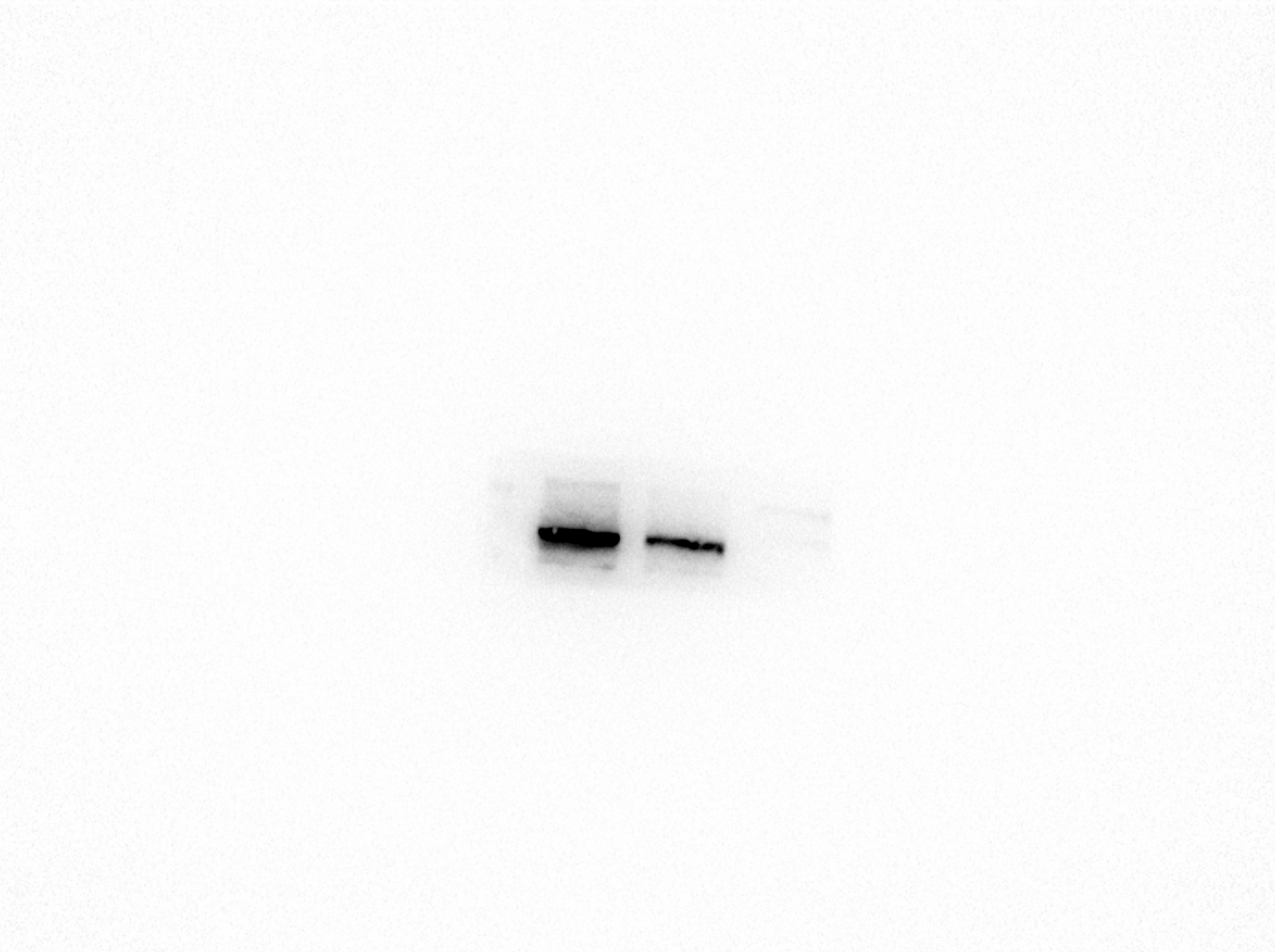

Supplement: Supplementary file 10 [file DataSheet_10.zip › TLR-4/Fig.5.4.tif]

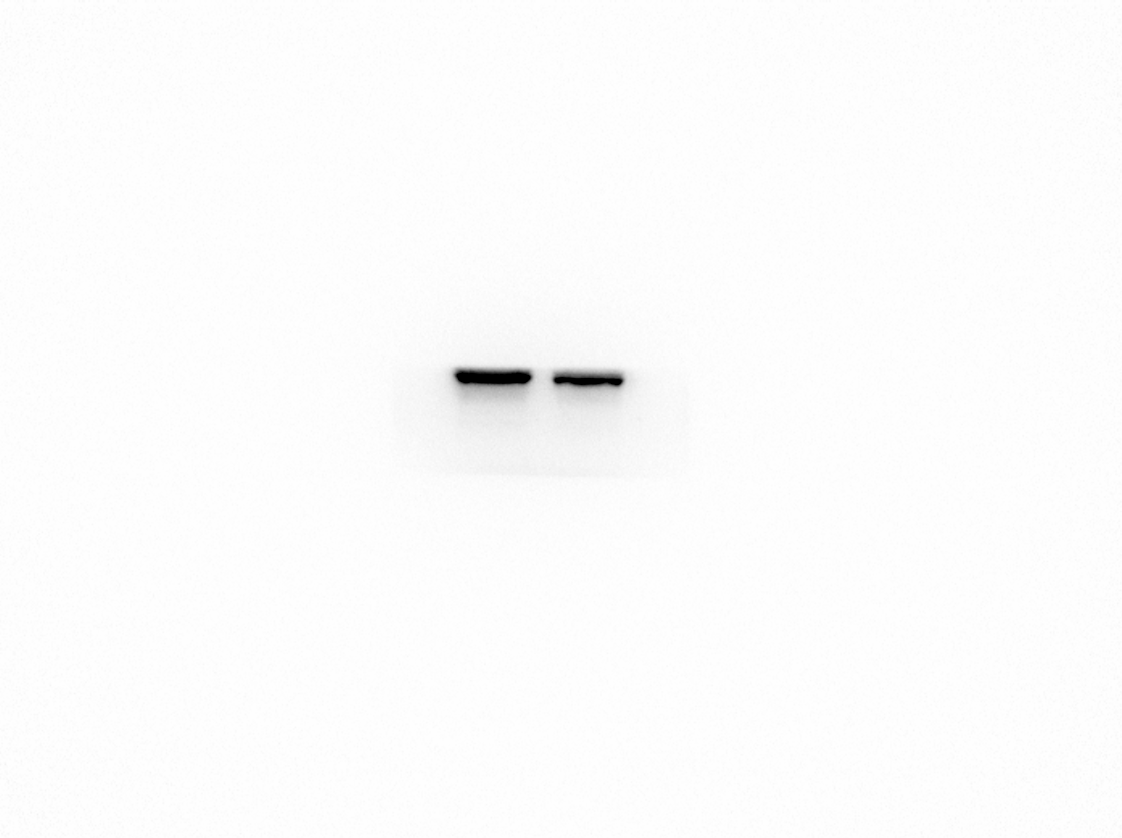

Supplement: Supplementary file 10 [file DataSheet_10.zip › TLR-4/Fig.7.2.tif]

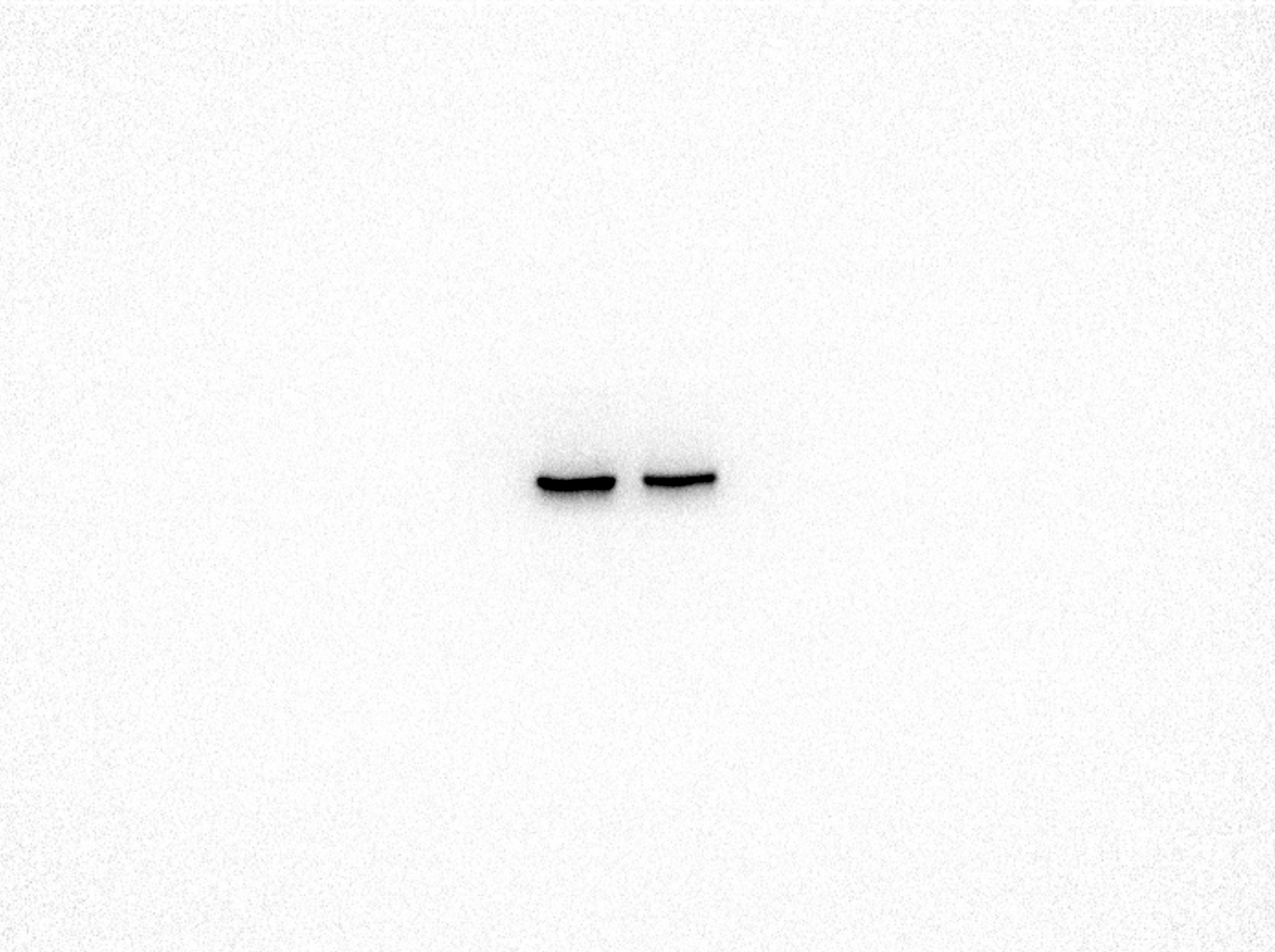

Supplement: Supplementary file 10 [file DataSheet_10.zip › TLR-4/Fig.7.3.tif]

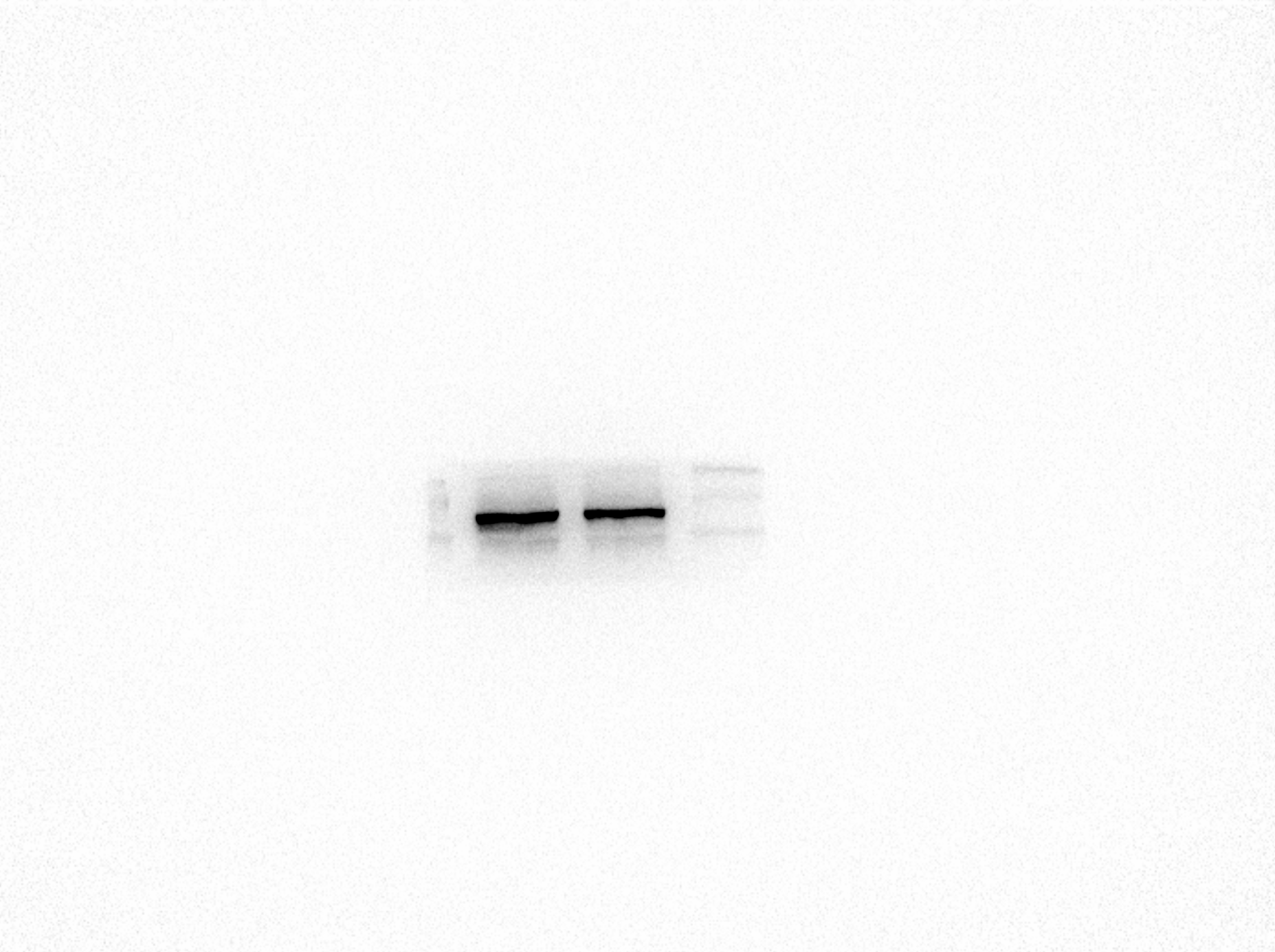

Supplement: Supplementary file 10 [file DataSheet_10.zip › TLR-4/Fig.7.4.tif]
